# Nonlinear distance measures under the framework of Pythagorean fuzzy sets with applications in problems of pattern recognition, medical diagnosis, and COVID-19 medicine selection

**DOI:** 10.1186/s43088-023-00375-8

**Published:** 2023-04-24

**Authors:** Palash Dutta, Gourangajit Borah, Brindaban Gohain, Rituparna Chutia

**Affiliations:** 1 Department of Mathematics, Dibrugarh University, Dibrugarh, Assam 786004 India; 2 Department of Mathematics, Cotton University, Guwahati, Assam 781001 India

**Keywords:** Chordal distance, Non-Archimedean norm, Pythagorean fuzzy set, Pattern recognition, Medical diagnosis

## Abstract

**Background:**

The concept of Pythagorean fuzzy sets (PFSs) is an utmost valuable mathematical framework, which handles the ambiguity generally arising in decision-making problems. Three parameters, namely membership degree, non-membership degree, and indeterminate (hesitancy) degree, characterize a PFS, where the sum of the square of each of the parameters equals one. PFSs have the unique ability to handle indeterminate or inconsistent information at ease, and which demonstrates its wider scope of applicability over intuitionistic fuzzy sets.

**Results:**

In the present article, we opt to define two nonlinear distances, namely generalized chordal distance and non-Archimedean chordal distance for PFSs. Most of the established measures possess linearity, and we cannot incorporate them to approximate the nonlinear nature of information as it might lead to counter-intuitive results. Moreover, the concept of non-Archimedean normed space theory plays a significant role in numerous research domains. The proficiency of our proposed measures to overcome the impediments of the existing measures is demonstrated utilizing twelve different sets of fuzzy numbers, supported by a diligent comparative analysis. Numerical examples of pattern recognition and medical diagnosis have been considered where we depict the validity and applicability of our newly constructed distances. In addition, we also demonstrate a problem of suitable medicine selection for COVID-19 so that the transmission rate of the prevailing viral pandemic could be minimized and more lives could be saved.

**Conclusions:**

Although the issues concerning the COVID-19 pandemic are very much challenging, yet it is the current need of the hour to save the human race. Furthermore, the justifiable structure of our proposed distances and also their feasible nature suggest that their applications are not only limited to some specific research domains, but decision-makers from other spheres as well shall hugely benefit from them and possibly come up with some further extensions of the ideas.

## Background

Although our classical concept of set theory is capable of answering or representing most of the scenarios, there also exist certain situations when the ideas of two-valued logic (set theory) are not sufficient enough to completely describe the phenomenon and to handle the uncertainty which normally exists in almost all real-life phenomenon. Such inefficiency of the two-valued logic is due to the fact that they are completely unable to represent the uncertain information, and they can only convey about the occurrence or non-occurrence of an event but nothing more than that. Thus, in order to overcome those lacunas, Zadeh in 1965 [1] developed the concept of fuzzy sets. Since then, fuzzy sets have seen numerous applications in various fields to handle the uncertainty inherent in them. There is no denying the fact that almost every kind of information or knowledge which is available in the real-life setting is in a way vague, or imprecise in nature.

However, certain extensions of fuzzy sets were also proposed according to the need to represent the uncertain information suitably, which are intuitionistic fuzzy sets (IFSs) [2], interval-valued fuzzy sets [3], fuzzy multisets [4], etc. A single membership function that assumes values between 0 and 1 characterizes a fuzzy set. But in a real-life setting, there also arises a need to represent its non-membership degree too, and we cannot merely say that the non-membership degree is given by 1 minus membership degree. Hence, in order to give more meaningful insight, it was Atanassov [2] who generalized the idea of fuzzy sets and proposed the new variant of fuzzy sets known as IFSs, which are characterized by both membership and non-membership degrees, and according to Atanassov, IFSs should be characterized by an added degree, known as the degree of indeterminacy or non-determinacy, which is defined as 1 minus membership degree minus non-membership degree. For an IFS $P = \left( {\mu_{P} \left( x \right),\nu_{P} \left( x \right)} \right)$ in $X$, the inequality $\mu_{P} \left( x \right) + \nu_{P} \left( x \right) \le 1$ must be satisfied and the degree of indeterminacy ($\pi_{P}$) is $\pi_{P} \left( x \right) = 1 - \mu_{P} \left( x \right) - \nu_{P} \left( x \right)$. Later, it was Yager [5], who initially pondered that the condition for IFSs, $\mu + \nu \le 1$ could not only be limited to this and it could be further extended.

Hence, in 2013, Yager and Abbasov [6] and Yager [5] substituted the constraint condition of $\mu + \nu \le 1$ by $\mu^{2} + \nu^{2} \le 1$ and proposed a new class of fuzzy sets by the name of Pythagorean fuzzy sets (PFSs). In other words, the membership values of PFSs are ordered pairs of the form $\left( {\mu ,\nu } \right)$ which satisfy the inequality of $\mu^{2} + \nu^{2} \le 1$. Clearly, PFSs are extensions of IFSs since the space of intuitionistic membership grades is inclusive under the space of Pythagorean membership grades. Also, according to Yager and Abbasov [6] there exists some relationship between complex numbers and Pythagorean membership degrees, and it was established that Pythagorean degrees are necessarily a subclass of complex numbers and are referred to as $\left( {\prod - i} \right)$ numbers. PFSs have more powerful applicability and are more reliable than IFSs, as far as dealing with the uncertainty-led daily life situations are concerned, since there are certain scenarios where IFSs are not suitable to be used, but PFSs could be applied. Say, for instance, to represent the belongingness of an element to a set, a certain element has membership degree value, $\mu = 0.7$ and the corresponding non-membership degree is $\nu = 0.7$. Clearly, $\mu + \nu = 0.7 + 0.6 = 1.3 > 1$, and therefore, IFSs could not be used for violating its constraint condition. Whereas we can use PFSs since the constraint condition $\mu^{2} + \nu^{2} = 0.49 + 0.36 = 0.85 \le 1$ is fulfilled. Afterward, PFSs received much more recognition and concern from researchers all around the globe. Yager and Abbasov [6] solved multicriteria decision-making (MCDM) problems based on aggregation operators on PFSs. Recently, several prominent works on PFSs can be noted in the literature. For instance, Yager [7] introduced the Pythagorean membership grades into problems of MCDM. Zhang and Xu [8] extended the method for TOPSIS (Technique for Order Preference by Similarity to an Ideal Solution) to solve decision-making problems with PFSs. Peng and Yang [9] developed certain significant results for PFSs. Moreover, they also proposed the Choquet integral for PFSs in 2016 [10]. Later, Ren et al. [11] elaborated the TODIM (an acronym in Portuguese of interactive and multicriteria decision making) approach for solving MCDM problems with Pythagorean fuzzy preference information. Garg [12] made use of the Einstein operations to devise a novel generalized aggregation operator for Pythagorean fuzzy numbers (PFNs). Thereafter, Garg [13] also devised a new accuracy function for solving MCDM problems, provided the uncertain information is represented by interval-valued Pythagorean fuzzy sets. Zhang [14] tackled group decision-making problems with the help of a new similarity measure for PFSs. At that era, a relatively newer concept of complex PFSs was introduced by Dick et al. [15]. Peng et al. [16] discussed about information measures in the Pythagorean fuzzy setting and mentioned some of their applications. Then, it was Zeng [17], who introduced OWA (ordered weighted averaging) approach into Pythagorean fuzzy group decision-making problems. Thereafter, Garg [18] introduced the concept of geometric aggregation operators into tackling MCDM problems. Qin et al. [19] considered generalized weighted distance measures for PFSs. Later, a regret theory and prospect theory-based score function and a novel distance measure for PFSs were investigated by Peng and Dai [20]. Thereafter, Biswas and Sarkar [21] put forward a point operator-based similarity measure for solving group decision-making problems involving PFSs. Wei and Wei [22] proposed several cosine function-based similarity measures for PFSs. Li and Mao [23] gave some new similarity and distance measures for PFSs overcoming the previous discrepancies [24] and demonstrating several multifarious applications. Xiao and Ding [25] proposed a divergence measure for PFSs that aids in medical diagnosis procedures. Adabitabar et al. [26] formulated a fruitful similarity measure for PFSs, which is efficient and robust enough to handle different real-life scenarios.

Distance measure acts as a research hotspot in fuzzy set theory owing to its immense applications in the field of machine learning, medical diagnosis, pattern recognition, signal processing, and many more. In this regard, several fruitful distance measures for PFSs were proposed by Li and Zeng [27]; Chen [28]; Ejegwa [29]; Hussian and Yang [30]; Baccour and Alimi [32]; Yang and Chiclana [33]; Wang and Xin [34]; Jin et al. [35]; Song et al. [36] and so on. Ejegwa and Awolola [37] tackled pattern recognition problems via some novel distance measures for PFSs. Sarkar and Biswas [38] devised a novel distance measure that can be applied to complex problems of transportation management. Mahanta and Panda [39] proposed a PFS-based distance measure and showed its application in the face-mask selection problem and few others.

Although several researchers and practitioners have provided some very fruitful versions of their own, the question of defining a universal or global distance to measure the distance between any two sets particularly in fuzzy set theory is still left an untouched problem. And as such to date, there has not been any such established metric (or distance) to measure the distance between two PFSs to the best of the author’s knowledge. From our acquired knowledge, we know that in order to construct a distance (or metric), we can either achieve it via a topology (based on topology theory) or via a norm (based on normed space theory). Most of the established distances like Euclidean distance, Minkowski distance, Pearson correlation distance are all linear in nature, and they hold the Archimedean property. But there arise certain situations where we deal with the nonlinear nature of information, and approximating them via linear distances may lead to inappropriate results. Due to this existing inadequacy, in the present article, we devise two notions of nonlinear distances for PFSs, namely generalized chordal distance and non-Archimedean chordal distance, where the core idea for constructing them came from complex analysis and non-Archimedean normed space theory, respectively. Researchers from the field of physics and mathematics background know about the crucial importance of non-Archimedean normed space theory, as they have widespread applications in numerous research domains, particularly in clustering algorithms. Later, it has been established that the newly constructed distances are more coherent and they can overcome the inadequacies of the existing distance measures, and further they apply to various decision-making instances.

The rest of the paper is structured as follows: in Sect. 2, we review some useful mathematical definitions and also provide a firm mathematical framework that will be necessitated to understand the newly proposed notions revealed in the subsequent sections. In Sect. 3, we list down some of the existing distance measures for PFSs, and then, we provide the definitions for our newly constructed nonlinear distances. Section 4 demonstrates some of the impediments of the existing measures, and we show how our proposed measures are efficient, feasible, and worthy of due consideration over those measures. In Sect. 5, we establish the applicability and rationality of our proposed distance measures by illustrating few numerical scenarios. For instance, problems of pattern recognition, medical diagnosis, and COVID-19 medicine selection are discussed in detail. Also, we conduct an in-depth sensitivity analysis for our newly constructed distance measures. Finally, some concluding remarks fill up Sect. 6.

## Methods and basic preliminaries

In this section, we discuss some of the basic concepts and ideas related to IFS and PFS. Moreover, a firm mathematical background is provided on few other concepts, which will be necessitated in our study.

### IFS and PFS

#### Definition 1

(Atanassov’s IFSs) [2] Suppose we consider any universe of discourse $U$(say), then according to Atanassov, a set $P$ can be called an IFS when it has the form.

1 $$P = \left\{ {\left( {x,\mu_{P} \left( x \right),\nu_{P} \left( x \right)} \right)|x \in U} \right\}$$

where the degree of belongingness (membership) and degree of non-belongingness (non-membership) of the element $x \in U$ to the set $P$ are indicated by functions $\mu_{P} :U \to [0,1]$ and $\nu_{P} :U \to [0,1]$, respectively. In addition to that, the condition of $0 \le \mu_{P} \left( x \right) \le 1$, $0 \le \nu_{P} \left( x \right) \le 1$ and $0 \le \mu_{P} \left( x \right) + \nu_{P} \left( x \right) \le 1$ must be satisfied.

Atanassov added one more degree which he called as the degree of hesitation of $x$ to $P$, and defined it as, $\pi_{P} \left( x \right) = 1 - \mu_{P} \left( x \right) - \nu_{P} \left( x \right)$.

#### Definition 2

(Yager’s PFSs) [5] Yager came up with the idea of PFSs as a solution to some exceptional cases or situations, when the sum of membership degree and non-membership degree exceeds 1 and as a result IFS theory fails to be applied.

According to Yager, a PFS $Q$ in a finite universe $U$ should be defined as

2 $$Q = \left\{ {\left( {x,\mu_{Q} \left( x \right),\nu_{Q} \left( x \right)} \right)|x \in U} \right\}$$

where the functions, $\mu_{Q} :U \to [0,1]$ and $\nu_{Q} :U \to [0,1]$, respectively, denote the membership and non-membership degree of the element $x \in Q$, provided the condition of $0 \le \mu_{Q}^{2} \left( x \right) + \nu_{Q}^{2} \left( x \right) \le 1$ is satisfied.

Here, the degree of indeterminacy (hesitation) takes the form given by

3 $$\pi_{P} \left( x \right) = \sqrt {1 - \mu_{Q}^{2} \left( x \right) - \nu_{Q}^{2} \left( x \right)}$$

We may also refer to a Pythagorean fuzzy number (PFN) as $Q = \left( {\mu_{Q} \left( x \right),\nu_{Q} \left( x \right)} \right)$ for convenience (Garg [40]).

#### Definition 3

(Some operations on PFSs) [6, 7] Hereby, we define some basic operations involving PFSs. For that purpose, suppose we consider the collection of all PFSs defined on a universe $U$, as $PFS\left( U \right)$, then for any two PFSs, $P = \left( {\mu_{P} \left( x \right),\nu_{P} \left( x \right)} \right)$ and $Q = \left( {\mu_{Q} \left( x \right),\nu_{Q} \left( x \right)} \right)$, the following properties hold:

(i) *Inclusion* Any PFS $P$ is said to be a subset of another PFS $Q$, that is, $P \subseteq Q$ if and only if for all $x \in U$, we have $\mu_{P} \left( x \right) \le \mu_{Q} \left( x \right)$, and $\nu_{P} \left( x \right) \ge \nu_{Q} \left( x \right)$;
(ii) *Equality* Any two PFSs $P$ and $Q$ are said to be equal, that is, $P = Q$ if and only if $\forall x \in U$,$\mu_{P} \left( x \right) = \mu_{Q} \left( x \right)$, and $\nu_{P} \left( x \right) = \nu_{Q} \left( x \right)$;

(iii) *Complement* For any PFS $P$ considered, its complement is defined by
  $$P^{C} = \left\{ {\nu_{P} \left( x \right),\mu_{P} \left( x \right)} \right\},\forall x \in U.$$

(iv) *Intersection* The intersection between two PFSs $P$ and $Q$ can be defined as,
  $$P \cap Q = \left\{ {\min \left( {\mu_{P} \left( x \right),\mu_{Q} \left( x \right)} \right),\max \left( {\mu_{P} \left( x \right),\mu_{Q} \left( x \right)} \right)} \right\},\;\forall x \in U;$$

(xxii) *Union* Similarly, the union operation between two PFSs $P$ and $Q$ is defined as,
  $$P \cup Q = \left\{ {\max \left( {\mu_{P} \left( x \right),\mu_{Q} \left( x \right)} \right),\min \left( {\mu_{P} \left( x \right),\mu_{Q} \left( x \right)} \right)} \right\},\;\forall x \in U.$$

### Comparison between IFS and PFS

The differences that exist between IFS and PFS can be grouped into two major factors:

#### Constraint condition

First major difference, which is notable, is that of the different constraint conditions, as $\mu_{P} \left( x \right),\nu_{P} \left( x \right) \in [0,1]$ for a set $P$ and an element $x \in P$, the constraint condition for IFSs is $\mu_{P} \left( x \right) + \nu_{P} \left( x \right) \le 1$, whereas for PFSs, it takes the form $\mu_{P}^{2} \left( x \right) + \nu_{P}^{2} \left( x \right) \le 1$. Accordingly, the degree of hesitation for IFSs is $\pi_{P} \left( x \right) = 1 - \mu_{P} \left( x \right) - \nu_{P} \left( x \right)$, whereas the same degree in case of PFSs is $\pi_{P} \left( x \right) = \sqrt {1 - \mu_{P}^{2} \left( x \right) - \nu_{P}^{2} \left( x \right)}$. Moreover, as PFSs are a generalization of IFSs, the space of intuitionistic membership grades is smaller than that of Pythagorean membership grades. That is, a PFS is necessarily an IFS, but the reverse is not true. Furthermore, PFSs cannot only handle the situations when the degree of membership and non-membership degree is greater than 1, where IFS theory fails, but in addition to that, it is also capable of handling the indeterminate information, which normally exists in real-life scenarios.

#### Complement operator

The second difference lies in the definition of the complement operator for IFN, say $P = \left( {\mu_{P} ,\nu_{P} } \right)$ and PFN, say $Q = \left( {\mu_{Q} ,\nu_{Q} } \right)$.

Precisely, for any $$\alpha \in U$$, the complement operator for IFN, $$P^{C} = \left( {\nu_{P} ,\mu_{P} } \right)$$ is proposed as per the Sugeno class of components, $$C\left( \alpha \right) = \frac{{\left( {1 - \alpha } \right)}}{{\left( {1 + \lambda \alpha } \right)}}\,\,\left( {\lambda \in \left( { - 1,\infty } \right)} \right)$$ when $$\lambda = 0\,\,\left( {i.e.\,C\left( \alpha \right) = 1 - \alpha } \right)$$, whereas the complement operator for PFN, $$Q^{C} = \left( {\nu_{Q} ,\mu_{Q} } \right)$$ is defined according to Yager class of components, $$C\left( \alpha \right) = \left( {1 - \alpha^{\sigma } } \right)^{{{\raise0.7ex\hbox{$1$} \!\mathord{\left/ {\vphantom {1 \sigma }}\right.\kern-0pt} \!\lower0.7ex\hbox{$\sigma $}}}} \,\,\left( {\sigma \in \left( {0,\infty } \right)} \right)$$ when $$\sigma = 2\,\,\left( {i.e.\,C\left( \alpha \right) = \sqrt {1 - \alpha^{2} } } \right)$$.

### Chordal distance and non-Archimedean chordal distance

In this segment, some prerequisite concepts necessary to have a proper visualization of the aforementioned terms are presented. Very briefly, we define what is a norm, distance (or metric), generalized chordal distance, non-Archimedean norm, non-Archimedean valuation, and finally non-Archimedean chordal distance.

#### Definition 4

(Norm) [41] For any non-empty set $A$, a norm is defined as a mapping ${\rm N}:A \to {\mathbb{R}}^{ + }$, such that it satisfies the conditions mentioned below,

(i) ${\rm N}\left( x \right) = 0$ iff $x = 0$, for any $x \in A$;
(ii) $${\rm N}\left( {xy} \right) = {\rm N}\left( x \right){\rm N}\left( y \right)$$, for $$x,y \in A$$;
(iii) ${\rm N}\left( {x + y} \right) \le {\rm N}\left( x \right) + {\rm N}\left( y \right)$, for $x,y \in A$, which is popularly known as the triangle inequality.

Here, ${\mathbb{R}}^{ + }$ denotes the extended real plane, $i.e.\,\,{\mathbb{R}}^{ + } = [0,\infty )$.

#### Remark 1

An example of a very widely used norm is the standard $l_{p}$ norm, which has the form.

$$\left\| x \right\|_{p} = \left( {\sum\limits_{i = 1}^{n} {\left| {x_{i} } \right|^{p} } } \right)^{{{\raise0.7ex\hbox{$1$} \!\mathord{\left/ {\vphantom {1 p}}\right.\kern-0pt} \!\lower0.7ex\hbox{$p$}}}}$$, where for $$p = 1$$ ($$l_{1}$$ norm) we obtain the taxicab norm or Manhattan distance, and $$p = 2$$ ($$l_{2}$$ norm) refers to the Euclidean norm. For $$p = \infty$$ or $$l_{\infty }$$ norm, we obtain the infinity or maximum norm.

#### Definition 5

(Distance or metric) [41] By distance or metric, we mean a mapping ‘$d$’ on a set $A$, such that, $d:A \times A \to {\mathbb{R}}^{ + }$, and which satisfies the conditions,

(i) $$d\left( {x,y} \right) \ge 0$$ for $$x,y \in A$$;
(ii) $d\left( {x,y} \right) = 0$ if and only if $x = y$;
(iii) $$d\left( {x,y} \right) = d\left( {y,x} \right)$$;
(iv) $$d\left( {x,z} \right) \le d\left( {x,y} \right) + d\left( {y,z} \right)$$, for $$x,y,z \in A$$.

#### Remark 2

The distance and norm are interrelated by the condition,

4 $$d\left( {x,y} \right) = {\rm N}\left( {x - y} \right).$$

#### Definition 6

(Generalized chordal distance) [41] The generalized chordal metric (distance) denoted by ‘$D_{{{\text{Chd}}}}$’ and defined as a mapping $D_{{{\text{Chd}}}} :A \times A \to [0,\infty )$ such that, for $x,y \in A$ (non-empty set), we have,

5 $$D_{{{\text{Chd}}}} \left( {x,y} \right) = \frac{{\left\| {x - y} \right\|}}{{\sqrt {1 + \left\| x \right\|^{2} } \sqrt {1 + \left\| y \right\|^{2} } }}$$

where $\left\| {\,.\,} \right\|$ denotes a norm or an absolute value function.

#### Definition 7

(non-Archimedean norm) [41] As per the definition of a norm already discussed, when we replace the third condition of triangular inequality by the condition, ${\rm N}\left( {a + b} \right) \le \max \left\{ {{\rm N}\left( a \right),{\rm N}\left( b \right)} \right\}$, for $a,b \in A$ (known as the ultrametric inequality), what we obtain is called a non-Archimedean norm.

#### Definition 8

(non-Archimedean valuation) [41] For a function or mapping to be termed as a non-Archimedean valuation, we must have $\left| {\,.\,} \right|:A \to {\mathbb{R}}^{ + }$ such that the following properties hold,

(i) $$\left| x \right| = 0$$ iff $$x = 0$$, for $$x \in A$$;
(ii) $$\left| {xy} \right| = \left| x \right|\left| y \right|$$, for $$x,y \in A$$;
(iii) $$\left| {x + y} \right| \le \max \left\{ {\left| x \right|,\left| y \right|} \right\}$$, for $$x,y \in A$$;
(iv) $\exists \,\,x \in A$ such that $\left| x \right| = 0,1$. (Non-triviality condition)

#### Definition 9

(non-Archimedean chordal distance) [41] The non-Archimedean chordal distance denoted by ‘$D_{{{\text{nAChd}}}}^{\lambda }$’ and defined as a mapping $D_{{{\text{nAChd}}}}^{\lambda } :A \times A \to [0,\infty )$ such that, for $x,y \in A$(non-empty set) and $\lambda \in [0,1]$, we have.

6 $$D_{{{\text{nAChd}}}}^{\lambda } \left( {x,y} \right) = \frac{{\left| {x - y} \right|}}{{\max \left\{ {\lambda ,\left| x \right|} \right\}\max \left\{ {\lambda ,\left| y \right|} \right\}}}$$

where $\left| {\,.\,} \right|$ denotes a non-Archimedean valuation or an absolute value function.

## New types of distance measures induced by existing measures

As a fruitful means of differentiating between two objects, a variety of distance or dissimilarity measures has been frequently used which are presented below. We then define our proposed distance measures and illustrate some of their desirable properties.

### Existing measures

Let us review some of the widely applied distance measures pertinent to PFSs. So, for any two PFSs $P = \left( {\mu_{P} ,\nu_{P} } \right)$ and $Q = \left( {\mu_{Q} ,\nu_{Q} } \right)$, defined in the finite universe say $U = \left\{ {x_{1} ,x_{2} ,...,x_{n} } \right\}$, we have.

(i) Hamming distance measure ($D_{H}$) [31]
  7 $$D_{H} \left( {P,Q} \right) = \frac{1}{2}\sum\limits_{i = 1}^{n} {\left( {\left| {\mu_{P}^{2} \left( {x_{i} } \right) - \mu_{Q}^{2} \left( {x_{i} } \right)} \right| + \left| {\nu_{P}^{2} \left( {x_{i} } \right) - \nu_{Q}^{2} \left( {x_{i} } \right)} \right| + \left| {\pi_{P}^{2} \left( {x_{i} } \right) - \pi_{Q}^{2} \left( {x_{i} } \right)} \right|} \right)}$$
(ii) Euclidean distance measure ($D_{E}$) [31]
  8 $$D_{E} \left( {P,Q} \right) = \left( {\frac{1}{2}\sum\limits_{i = 1}^{n} {\left( {\mu_{P}^{2} \left( {x_{i} } \right) - \mu_{Q}^{2} \left( {x_{i} } \right)} \right)^{2} + \left( {\nu_{P}^{2} \left( {x_{i} } \right) - \nu_{Q}^{2} \left( {x_{i} } \right)} \right)^{2} + \left( {\pi_{P}^{2} \left( {x_{i} } \right) - \pi_{Q}^{2} \left( {x_{i} } \right)} \right)^{2} } } \right)^{\frac{1}{2}}$$
(iii) Normalized Hamming distance measure ($D_{{{\text{nH}}}}$) [31]
  9 $$D_{{{\text{nH}}}} \left( {P,Q} \right) = \frac{1}{2n}\sum\limits_{i = 1}^{n} {\left( {\left| {\mu_{P}^{2} \left( {x_{i} } \right) - \mu_{Q}^{2} \left( {x_{i} } \right)} \right| + \left| {\nu_{P}^{2} \left( {x_{i} } \right) - \nu_{Q}^{2} \left( {x_{i} } \right)} \right| + \left| {\pi_{P}^{2} \left( {x_{i} } \right) - \pi_{Q}^{2} \left( {x_{i} } \right)} \right|} \right)}$$
(iv) Normalized Euclidean distance measure ($D_{nE}$) [31]
  10 $$D_{{{\text{nE}}}} \left( {P,Q} \right) = \left( {\frac{1}{2n}\sum\limits_{i = 1}^{n} {\left( {\mu_{P}^{2} \left( {x_{i} } \right) - \mu_{Q}^{2} \left( {x_{i} } \right)} \right)^{2} + \left( {\nu_{P}^{2} \left( {x_{i} } \right) - \nu_{Q}^{2} \left( {x_{i} } \right)} \right)^{2} + \left( {\pi_{P}^{2} \left( {x_{i} } \right) - \pi_{Q}^{2} \left( {x_{i} } \right)} \right)^{2} } } \right)^{\frac{1}{2}}$$
(v) Baccour and Alimi’s First Squared distance measure ($D_{BA}^{1}$) [32]
  11 $$D_{{{\text{BA}}}}^{1} \left( {P,Q} \right) = \frac{1}{2n}\sum\limits_{i = 1}^{n} {\left( {\left( {\sqrt {\mu_{P} \left( {x_{i} } \right)} - \sqrt {\mu_{Q} \left( {x_{i} } \right)} } \right)^{2} + \left( {\sqrt {\nu_{P} \left( {x_{i} } \right)} - \sqrt {\nu_{Q} \left( {x_{i} } \right)} } \right)^{2} } \right)}$$
(vi) Baccour and Alimi’s second squared distance measure ($D_{BA}^{2}$) [32]
  12 $$D_{{{\text{BA}}}}^{2} \left( {P,Q} \right) = \frac{1}{4n}\sum\limits_{i = 1}^{n} \, \left( {\sqrt {\left| {\mu_{P}^{2} \left( {x_{i} } \right) - \mu_{Q}^{2} \left( {x_{i} } \right)} \right|} + \sqrt {\left| {\nu_{P}^{2} \left( {x_{i} } \right) - \nu_{Q}^{2} \left( {x_{i} } \right)} \right|} } \right)^{2}$$
(vii) Grzegorzewski’s distance measure ($D_{G}$) [3]
  13 $$D_{G} \left( {P,Q} \right) = \frac{1}{n}\sum\limits_{i = 1}^{n} {\max \left\{ {\left| {\mu_{P}^{2} \left( {x_{i} } \right) - \mu_{Q}^{2} \left( {x_{i} } \right)} \right|,\left| {\nu_{P}^{2} \left( {x_{i} } \right) - \nu_{Q}^{2} \left( {x_{i} } \right)} \right|} \right\}}$$
(viii) Yang and Chiclana’s distance measure ($D_{YF}$) [33]
  14 $$D_{{{\text{YF}}}} \left( {P,Q} \right) = \frac{1}{n}\sum\limits_{i = 1}^{n} {\max \left( {\left| {\mu_{P}^{2} \left( {x_{i} } \right) - \mu_{Q}^{2} \left( {x_{i} } \right)} \right|,\left| {\nu_{P}^{2} \left( {x_{i} } \right) - \nu_{Q}^{2} \left( {x_{i} } \right)} \right|,\left| {\pi_{P}^{2} \left( {x_{i} } \right) - \pi_{Q}^{2} \left( {x_{i} } \right)} \right|} \right)}$$
(ix) Wang and Xin’s distance measure ($D_{WX}$) [34]
  15 $$D_{{{\text{WX}}}} \left( {P,Q} \right) = \frac{1}{n}\sum\limits_{i = 1}^{n} \, \left[ {\frac{{\left| {\mu_{P}^{2} \left( {x_{i} } \right) - \mu_{Q}^{2} \left( {x_{i} } \right)} \right| + \left| {\nu_{P}^{2} \left( {x_{i} } \right) - \nu_{Q}^{2} \left( {x_{i} } \right)} \right|}}{4} + \frac{{\max \left( {\left| {\mu_{P}^{2} \left( {x_{i} } \right) - \mu_{Q}^{2} \left( {x_{i} } \right)} \right|,\left| {\nu_{P}^{2} \left( {x_{i} } \right) - \nu_{Q}^{2} \left( {x_{i} } \right)} \right|} \right)}}{2}} \right]$$
(x) Jin et al.’s distance measure ($D_{{{\text{JHP}}}}$) [35]
  16 $$D_{{{\text{JHP}}}} \left( {P,Q} \right) = \frac{1}{4n}\sum\limits_{i = 1}^{n} \, \left( \begin{gathered} \left| {\mu_{P}^{2} \left( {x_{i} } \right) - \mu_{Q}^{2} \left( {x_{i} } \right)} \right| + \left| {\nu_{P}^{2} \left( {x_{i} } \right) - \nu_{Q}^{2} \left( {x_{i} } \right)} \right| + \left| {\pi_{P}^{2} \left( {x_{i} } \right) - \pi_{Q}^{2} \left( {x_{i} } \right)} \right| \hfill \\ + 2\max \left\{ {\left| {\mu_{P}^{2} \left( {x_{i} } \right) - \mu_{Q}^{2} \left( {x_{i} } \right)} \right|,\left| {\nu_{P}^{2} \left( {x_{i} } \right) - \nu_{Q}^{2} \left( {x_{i} } \right)} \right|,\left| {\pi_{P}^{2} \left( {x_{i} } \right) - \pi_{Q}^{2} \left( {x_{i} } \right)} \right|} \right\} \hfill \\ \end{gathered} \right)$$
(xi) Song et al.’s distance measure ($D_{{{\text{SF}}}}$) [36]
  17 $$D_{{{\text{SF}}}} \left( {P,Q} \right) = 1 - \frac{1}{3n}\sum\limits_{i = 1}^{n} \, \left( \begin{gathered} 2\sqrt {\mu_{P} \left( {x_{i} } \right)\mu_{Q} \left( {x_{i} } \right)} + 2\sqrt {\nu_{P} \left( {x_{i} } \right)\nu_{Q} \left( {x_{i} } \right)} + \sqrt {\pi_{P} \left( {x_{i} } \right)\pi_{Q} \left( {x_{i} } \right)} \hfill \\ + \sqrt {\left( {1 - \mu_{P} \left( {x_{i} } \right)} \right)\left( {1 - \mu_{Q} \left( {x_{i} } \right)} \right)} + \sqrt {\left( {1 - \nu_{P} \left( {x_{i} } \right)} \right)\left( {1 - \nu_{Q} \left( {x_{i} } \right)} \right)} \hfill \\ \end{gathered} \right)$$
(xii) Ren et al.’s distance measure ($D_{RX}$) [11]
  18 $$D_{{{\text{RX}}}} \left( {P,Q} \right) = \left[ {\frac{1}{2n}\sum\limits_{i = 1}^{n} {\left\{ {\left( {\mu_{P}^{2} \left( {x_{i} } \right) - \mu_{Q}^{2} \left( {x_{i} } \right)} \right)^{2} + \left( {\nu_{P}^{2} \left( {x_{i} } \right) - \nu_{Q}^{2} \left( {x_{i} } \right)} \right)^{2} + \left( {\pi_{P}^{2} \left( {x_{i} } \right) - \pi_{Q}^{2} \left( {x_{i} } \right)} \right)^{2} } \right\}} } \right]^{\frac{1}{2}}$$
(xiii) Peng et al.’s distance measure ($D_{{{\text{PY}}}}$) [16]
  19 $$D_{{{\text{PY}}}} \left( {P,Q} \right) = \frac{1}{4n}\sum\limits_{i = 1}^{n} {\left( {\left| {\mu_{P}^{2} \left( {x_{i} } \right) - \mu_{Q}^{2} \left( {x_{i} } \right)} \right|^{2} + \left| {\nu_{P}^{2} \left( {x_{i} } \right) - \nu_{Q}^{2} \left( {x_{i} } \right)} \right|^{2} + \left| {\pi_{P}^{2} \left( {x_{i} } \right) - \pi_{Q}^{2} \left( {x_{i} } \right)} \right|^{2} } \right)}$$
(xiv) Ejegwa and Awolola’s distance measure ($D_{{{\text{EA}}}}$) [37]
  20 $$D_{{{\text{EA}}}} \left( {P,Q} \right) = \frac{1}{4n}\sum\limits_{i = 1}^{n} {\left\{ \begin{gathered} \left| {\mu_{P} \left( {x_{i} } \right) - \mu_{Q} \left( {x_{i} } \right)} \right| + \left| {\mu_{P} \left( {x_{i} } \right) - \nu_{P} \left( {x_{i} } \right)} \right| - \left| {\mu_{Q} \left( {x_{i} } \right) - \nu_{Q} \left( {x_{i} } \right)} \right| \hfill \\ \quad + \left| {\mu_{P} \left( {x_{i} } \right) - \pi_{P} \left( {x_{i} } \right)} \right| - \left| {\mu_{Q} \left( {x_{i} } \right) - \pi_{Q} \left( {x_{i} } \right)} \right| \hfill \\ \end{gathered} \right\}}$$
(xv) Sarkar and Biswas’s distance measure ($D_{{{\text{SB}}}}$) [38]
  21 $$D_{{{\text{SB}}}} \left( {P,Q} \right) = \frac{1}{3n}\sum\limits_{i = 1}^{n} {\left\{ \begin{gathered} \left| {\mu_{P}^{2} \left( {x_{i} } \right) - \mu_{Q}^{2} \left( {x_{i} } \right)} \right| + \left| {\nu_{P}^{2} \left( {x_{i} } \right) - \nu_{Q}^{2} \left( {x_{i} } \right)} \right| + \left| {\pi_{P}^{2} \left( {x_{i} } \right) - \pi_{Q}^{2} \left( {x_{i} } \right)} \right| \hfill \\ \quad + \left| {\max \left\{ {\mu_{P}^{2} \left( {x_{i} } \right),\nu_{Q}^{2} \left( {x_{i} } \right)} \right\} - \max \left\{ {\mu_{Q}^{2} \left( {x_{i} } \right),\nu_{P}^{2} \left( {x_{i} } \right)} \right\}} \right| \hfill \\ \end{gathered} \right\}}$$
(xvi) Mahanta and Panda’s distance measure ($D_{{{\text{MP}}}}$) [39]
  22 $$D_{{{\text{MP}}}} \left( {P,Q} \right) = \frac{1}{n}\sum\limits_{i = 1}^{n} {\frac{{\left| {\mu_{P}^{2} \left( {x_{i} } \right) - \mu_{Q}^{2} \left( {x_{i} } \right)} \right| + \left| {\nu_{P}^{2} \left( {x_{i} } \right) - \nu_{Q}^{2} \left( {x_{i} } \right)} \right|}}{{\mu_{P}^{2} \left( {x_{i} } \right) + \mu_{Q}^{2} \left( {x_{i} } \right) + \nu_{P}^{2} \left( {x_{i} } \right) + \nu_{Q}^{2} \left( {x_{i} } \right)}}}$$

#### Remark 3

It is noteworthy that the distance measures (i)–(iv) may also be referred to as Euclidean-like distance measures.

### Newly proposed distance measures

Based on the concepts discussed in Sect. 2.2, we are motivated to define two nonlinear distances for PFSs, as given below.

#### Generalized chordal distance for PFSs

For any two PFSs, $P = \left( {\mu_{P} \left( {x_{i} } \right),\nu_{P} \left( {x_{i} } \right)} \right)$ and $Q = \left( {\mu_{Q} \left( {x_{i} } \right),\nu_{Q} \left( {x_{i} } \right)} \right)$ defined in a finite universe, $U = \left\{ {x_{1} ,x_{2} ,...,x_{n} } \right\}$, the generalized chordal distance in PFSs is defined as

23 $$D_{{{\text{Chd}},p}} \left( {P,Q} \right) = \left( {\frac{1}{{2^{{\left( {1 - \tfrac{p}{2}} \right)}} n}}\sum\limits_{i = 1}^{n} {\left[ {\left\{ {D_{{{\text{Chd}}}} \left( {\mu_{P} \left( {x_{i} } \right),\mu_{Q} \left( {x_{i} } \right)} \right)} \right\}^{p} + \left\{ {D_{{{\text{Chd}}}} \left( {\nu_{P} \left( {x_{i} } \right),\nu_{Q} \left( {x_{i} } \right)} \right)} \right\}^{p} } \right]} } \right)^{{{\raise0.7ex\hbox{$1$} \!\mathord{\left/ {\vphantom {1 p}}\right.\kern-0pt} \!\lower0.7ex\hbox{$p$}}}}$$

To establish the validity and reasonability of Eq. (23), we state the following theorems.

##### Theorem 1

For any finite universe, $U = \left\{ {x_{1} ,x_{2} ,...,x_{n} } \right\}$, the proposed distance $D_{Chd,p} \left( {P,Q} \right)$ between PFSs $P$ and $Q$ must satisfy the following conditions:

**(C-1)** $$0 \le D_{{{\text{Chd}},p}} \left( {P,Q} \right) \le 1$$; (*Boundedness*)

**(C-2)** $$D_{{{\text{Chd}},p}} \left( {P,Q} \right) = 0 \Leftrightarrow P = Q$$; (*Separability*)

**(C-3)** $$D_{{{\text{Chd}},p}} \left( {P,Q} \right) = D_{{{\text{Chd}},p}} \left( {Q,P} \right)$$; (*Symmetricity*).

##### Proof

(**C-1**) We have the generalized chordal distance between PFSs $P$ and $Q$ is defined as.

$$D_{{{\text{Chd}},p}} \left( {P,Q} \right) = \left( {\frac{1}{{2^{{\left( {1 - \tfrac{p}{2}} \right)}} n}}\sum\limits_{i = 1}^{n} {\left[ {\left\{ {D_{{{\text{Chd}}}} \left( {\mu_{P} \left( {x_{i} } \right),\mu_{Q} \left( {x_{i} } \right)} \right)} \right\}^{p} + \left\{ {D_{{{\text{Chd}}}} \left( {\nu_{P} \left( {x_{i} } \right),\nu_{Q} \left( {x_{i} } \right)} \right)} \right\}^{p} } \right]} } \right)^{{{\raise0.7ex\hbox{$1$} \!\mathord{\left/ {\vphantom {1 p}}\right.\kern-0pt} \!\lower0.7ex\hbox{$p$}}}}$$

or, it can be written in more simplified form as,

$$D_{{{\text{Chd}},p}} \left( {P,Q} \right) = \left( {\frac{1}{{2^{{\left( {1 - \tfrac{p}{2}} \right)}} n}}\sum\limits_{i = 1}^{n} {\left[ {\left\{ {\frac{{\left\| {\mu_{P} \left( {x_{i} } \right) - \mu_{Q} \left( {x_{i} } \right)} \right\|}}{{\sqrt {1 + \left\| {\mu_{P} \left( {x_{i} } \right)} \right\|^{2} } \sqrt {1 + \left\| {\mu_{Q} \left( {x_{i} } \right)} \right\|^{2} } }}} \right\}^{p} + \left\{ {\frac{{\left\| {\nu_{P} \left( {x_{i} } \right) - \nu_{Q} \left( {x_{i} } \right)} \right\|}}{{\sqrt {1 + \left\| {\nu_{P} \left( {x_{i} } \right)} \right\|^{2} } \sqrt {1 + \left\| {\nu_{Q} \left( {x_{i} } \right)} \right\|^{2} } }}} \right\}^{p} } \right]} } \right)^{{{\raise0.7ex\hbox{$1$} \!\mathord{\left/ {\vphantom {1 p}}\right.\kern-0pt} \!\lower0.7ex\hbox{$p$}}}}$$

24 $${\text{Now}},\;{\text{since}}\;0 \le \mu_{P} \left( {x_{i} } \right),\mu_{Q} \left( {x_{i} } \right) \le 1,\;{\text{therefore}}\;0 \le \left\| {\mu_{P} \left( {x_{i} } \right) - \mu_{Q} \left( {x_{i} } \right)} \right\| \le 1.$$

However, $\sqrt {1 + \left\| {\mu_{P} \left( {x_{i} } \right)} \right\|^{2} } \ge 1$ and $\sqrt {1 + \left\| {\mu_{Q} \left( {x_{i} } \right)} \right\|^{2} } \ge 1$, as $\mu_{P} \left( {x_{i} } \right),\mu_{Q} \left( {x_{i} } \right) \ge 0$.

This implies that,

25 $$0 \le \frac{1}{{\sqrt {1 + \left\| {\mu_{P} \left( {x_{i} } \right)} \right\|^{2} } }} \le 1\quad {\text{and}},\quad 0 \le \frac{1}{{\sqrt {1 + \left\| {\mu_{Q} \left( {x_{i} } \right)} \right\|^{2} } }} \le 1$$

Combining Eqs. (24) and (25), we get,

26 $$\begin{aligned} 0 & \le \frac{{\left\| {\mu_{P} \left( {x_{i} } \right) - \mu_{Q} \left( {x_{i} } \right)} \right\|}}{{\sqrt {1 + \left\| {\mu_{P} \left( {x_{i} } \right)} \right\|^{2} } \sqrt {1 + \left\| {\mu_{Q} \left( {x_{i} } \right)} \right\|^{2} } }} \le 1 \\ & \Rightarrow \left( {\frac{{\left\| {\mu_{P} \left( {x_{i} } \right) - \mu_{Q} \left( {x_{i} } \right)} \right\|}}{{\sqrt {1 + \left\| {\mu_{P} \left( {x_{i} } \right)} \right\|^{2} } \sqrt {1 + \left\| {\mu_{Q} \left( {x_{i} } \right)} \right\|^{2} } }}} \right)^{p} \le 1\;({\text{for}}\;{\text{any}}\;{\text{value}}\;{\text{of}}\;p, \;{\text{the}}\;{\text{value}}\;{\text{is}}\;{\text{further}}\;{\text{smaller}} \\ \end{aligned}$$

By the same argument we have,

27 $$\left( {\frac{{\left\| {\nu_{P} \left( {x_{i} } \right) - \nu_{Q} \left( {x_{i} } \right)} \right\|}}{{\sqrt {1 + \left\| {\nu_{P} \left( {x_{i} } \right)} \right\|^{2} } \sqrt {1 + \left\| {\nu_{Q} \left( {x_{i} } \right)} \right\|^{2} } }}} \right)^{p} \le 1$$

We observe that, in each of the fractions, the numerator part is much smaller than the denominator part, which ultimately results in a value of the fraction being smaller than 1(one). Consequently, taking any positive power ($p$) of such an output will further reduce its value.

Therefore, from Eqs. (26) and (27), we can conclude that,

$$\begin{aligned} 0 & \le \left( {\frac{1}{{2^{{\left( {1 - \tfrac{p}{2}} \right)}} n}}\,\,\sum\limits_{i = 1}^{n} {\left[ {\left\{ {\frac{{\left\| {\mu_{P} \left( {x_{i} } \right) - \mu_{Q} \left( {x_{i} } \right)} \right\|}}{{\sqrt {1 + \left\| {\mu_{P} \left( {x_{i} } \right)} \right\|^{2} } \sqrt {1 + \left\| {\mu_{Q} \left( {x_{i} } \right)} \right\|^{2} } }}} \right\}^{p} + \left\{ {\frac{{\left\| {\nu_{P} \left( {x_{i} } \right) - \nu_{Q} \left( {x_{i} } \right)} \right\|}}{{\sqrt {1 + \left\| {\nu_{P} \left( {x_{i} } \right)} \right\|^{2} } \sqrt {1 + \left\| {\nu_{Q} \left( {x_{i} } \right)} \right\|^{2} } }}} \right\}^{p} } \right]} } \right)^{{{\raise0.7ex\hbox{$1$} \!\mathord{\left/ {\vphantom {1 p}}\right.\kern-0pt} \!\lower0.7ex\hbox{$p$}}}} \le 1 \\ & \Rightarrow 0 \le D_{Chd,p} \left( {P,Q} \right) \le 1. \\ \end{aligned}$$

(**C-2**) When $P = Q$, we have $\mu_{P} \left( {x_{i} } \right) = \mu_{Q} \left( {x_{i} } \right),\nu_{P} \left( {x_{i} } \right) = \nu_{Q} \left( {x_{i} } \right),\,\forall x_{i} \in U$.

Therefore, we have, $D_{{{\text{Chd}}}} \left( {\mu_{P} \left( {x_{i} } \right),\mu_{Q} \left( {x_{i} } \right)} \right) = 0$, $D_{{{\text{Chd}}}} \left( {\nu_{P} \left( {x_{i} } \right),\nu_{Q} \left( {x_{i} } \right)} \right) = 0$, and hence, $D_{{{\text{Chd}},p}} \left( {P,Q} \right) = 0$.

Conversely, when $D_{{{\text{Chd}},p}} \left( {P,Q} \right) = 0$, we have

$$\begin{aligned} & \left( {\frac{1}{{2^{{\left( {1 - \tfrac{p}{2}} \right)}} n}}\sum\limits_{i = 1}^{n} {\left[ {\left\{ {D_{{{\text{Chd}}}} \left( {\mu_{P} \left( {x_{i} } \right),\mu_{Q} \left( {x_{i} } \right)} \right)} \right\}^{p} + \left\{ {D_{{{\text{Chd}}}} \left( {\nu_{P} \left( {x_{i} } \right),\nu_{Q} \left( {x_{i} } \right)} \right)} \right\}^{p} } \right]} } \right)^{{{\raise0.7ex\hbox{$1$} \!\mathord{\left/ {\vphantom {1 p}}\right.\kern-0pt} \!\lower0.7ex\hbox{$p$}}}} = 0 \\ & \Rightarrow D_{{{\text{Chd}}}} \left( {\mu_{P} \left( {x_{i} } \right),\mu_{Q} \left( {x_{i} } \right)} \right) = 0;\;D_{{{\text{Chd}}}} \left( {\nu_{P} \left( {x_{i} } \right),\nu_{Q} \left( {x_{i} } \right)} \right) = 0 \\ & \Rightarrow \mu_{P} \left( {x_{i} } \right) = \mu_{Q} \left( {x_{i} } \right);\;\nu_{P} \left( {x_{i} } \right) = \nu_{Q} \left( {x_{i} } \right),\;\forall x_{i} \in U \\ & \Rightarrow P = Q \\ \end{aligned}$$

Therefore, condition (**C-2**) holds.

(**C-3**) The symmetric property is very much trivial.

We now state and prove an important lemma, which will be utilized in proving the subsequent theorem that follows.

##### Lemma 1

If $$0 \le p_{1} \le p_{2} \le p_{3} \le 1$$, then $$\left| {p_{1} - p_{3} } \right|\sqrt {1 + p_{2}^{2} } \ge \left| {p_{1} - p_{2} } \right|\sqrt {1 + p_{3}^{2} }$$.

##### Proof

Given $0 \le p_{1} \le p_{2} \le p_{3} \le 1$, then we evaluate.

$$\begin{aligned} \left| {p_{1} - p_{3} } \right|\sqrt {1 + p_{2} ^{2} } - \left| {p_{1} - p_{2} } \right|\sqrt {1 + p_{3} ^{2} } & = \left( {p_{3} - p_{1} } \right)\sqrt {1 + p_{2} ^{2} } - \left( {p_{2} - p_{1} } \right)\sqrt {1 + p_{3} ^{2} } \\ & = \left( {p_{3} \sqrt {1 + p_{2} ^{2} } - p_{2} \sqrt {1 + p_{3} ^{2} } } \right) + p_{1} \left( {\sqrt {1 + p_{3} ^{2} } - \sqrt {1 + p_{2} ^{2} } } \right) \\ & = \left[ {\frac{{p_{3} ^{2} \left( {1 + p_{2} ^{2} } \right) - p_{2} ^{2} \left( {1 + p_{3} ^{2} } \right)}}{{p_{3} \sqrt {1 + p_{2} ^{2} } + p_{2} \sqrt {1 + p_{3} ^{2} } }}} \right] + p_{1} \left[ {\frac{{\left( {1 + p_{3} ^{2} } \right) - \left( {1 + p_{2} ^{2} } \right)}}{{\sqrt {1 + p_{3} ^{2} } + \sqrt {1 + p_{2} ^{2} } }}} \right] \\ & = \frac{{\left( {p_{3} - p_{2} } \right)\left( {p_{3} + p_{2} } \right)}}{{p_{3} \sqrt {1 + p_{2} ^{2} } + p_{2} \sqrt {1 + p_{3} ^{2} } }} + \frac{{p_{1} \left( {p_{3} - p_{2} } \right)\left( {p_{3} + p_{2} } \right)}}{{\sqrt {1 + p_{3} ^{2} } + \sqrt {1 + p_{2} ^{2} } }} \\ & = \left( {\frac{1}{{p_{3} \sqrt {1 + p_{2} ^{2} } + p_{2} \sqrt {1 + p_{3} ^{2} } }} + \frac{{p_{1} }}{{\sqrt {1 + p_{3} ^{2} } + \sqrt {1 + p_{2} ^{2} } }}} \right)\left( {p_{3} - p_{2} } \right)\left( {p_{3} + p_{2} } \right) \\ & \ge 0,\;{\text{since}}\;p_{3} \ge p_{2} . \\ \end{aligned}$$

Hence, $$\left| {p_{1} - p_{3} } \right|\sqrt {1 + p_{2}^{2} } \ge \left| {p_{1} - p_{2} } \right|\sqrt {1 + p_{3}^{2} } .$$

##### Corollary 1

If $p_{i} \in {\mathbb{R}} \cap [0,1]$, then $\left| {p_{i} - p_{j} } \right| = \left\| {p_{i} - p_{j} } \right\|$ and also, $\left| {p_{i} } \right|^{2} = \left\| {p_{i} } \right\|^{2} = p_{i}^{2}$. So, the relation $\left\| {p_{1} - p_{3} } \right\|\sqrt {1 + \left\| {p_{2} } \right\|^{2} } \ge \left\| {p_{1} - p_{2} } \right\|\sqrt {1 + \left\| {p_{3} } \right\|^{2} }$ also holds.

##### Theorem 2

Let $D_{{{\text{Chd}},p}} :{\text{PFS}}\left( U \right) \times {\text{PFS}}\left( U \right) \to {\mathbb{R}}$ be a distance measure between two PFSs $P = \left( {\mu_{P} \left( {x_{i} } \right),\nu_{P} \left( {x_{i} } \right)} \right)$ and $Q = \left( {\mu_{Q} \left( {x_{i} } \right),\nu_{Q} \left( {x_{i} } \right)} \right)$, defined as.

$$D_{{{\text{Chd}},p}} \left( {P,Q} \right) = \left( {\frac{1}{{2^{{\left( {1 - \tfrac{p}{2}} \right)}} n}}\sum\limits_{i = 1}^{n} {\left[ {\left\{ {D_{{{\text{Chd}}}} \left( {\mu_{P} \left( {x_{i} } \right),\mu_{Q} \left( {x_{i} } \right)} \right)} \right\}^{p} + \left\{ {D_{{{\text{Chd}}}} \left( {\nu_{P} \left( {x_{i} } \right),\nu_{Q} \left( {x_{i} } \right)} \right)} \right\}^{p} } \right]} } \right)^{{{\raise0.7ex\hbox{$1$} \!\mathord{\left/ {\vphantom {1 p}}\right.\kern-0pt} \!\lower0.7ex\hbox{$p$}}}}$$

or, $$D_{{{\text{Chd}},p}} \left( {P,Q} \right) = \left( {\frac{1}{{2^{{\left( {1 - \tfrac{p}{2}} \right)}} n}}\sum\limits_{i = 1}^{n} {\left[ {\left\{ {\frac{{\left\| {\mu_{P} \left( {x_{i} } \right) - \mu_{Q} \left( {x_{i} } \right)} \right\|}}{{\sqrt {1 + \left\| {\mu_{P} \left( {x_{i} } \right)} \right\|^{2} } \sqrt {1 + \left\| {\mu_{Q} \left( {x_{i} } \right)} \right\|^{2} } }}} \right\}^{p} + \left\{ {\frac{{\left\| {\nu_{P} \left( {x_{i} } \right) - \nu_{Q} \left( {x_{i} } \right)} \right\|}}{{\sqrt {1 + \left\| {\nu_{P} \left( {x_{i} } \right)} \right\|^{2} } \sqrt {1 + \left\| {\nu_{Q} \left( {x_{i} } \right)} \right\|^{2} } }}} \right\}^{p} } \right]} } \right)^{{{\raise0.7ex\hbox{$1$} \!\mathord{\left/ {\vphantom {1 p}}\right.\kern-0pt} \!\lower0.7ex\hbox{$p$}}}}$$, then it satisfies the following axioms

- **A-1:** If $P \subseteq Q \subseteq R$, then $D_{{{\text{Chd}},p}} \left( {P,Q} \right) \le D_{{{\text{Chd}},p}} \left( {P,R} \right)$ and $D_{{{\text{Chd}},p}} \left( {Q,R} \right) \le D_{{{\text{Chd}},p}} \left( {P,R} \right)$ for $P,Q,R \in {\text{PFS}}\left( U \right)$. (*Containment property*).
- **A-2:** $D_{{{\text{Chd}},p}} \left( {P,P^{C} } \right) = 1,\;\forall \;P \in {\text{PFS}}\left( U \right)$ if and only if $P$ is a crisp set.
- **A-3:** $$D_{{{\text{Chd}},p}} \left( {P,Q} \right) = D_{{{\text{Chd}},p}} \left( {P^{C} ,Q^{C} } \right),\;\forall \;P,Q \in {\text{PFS}}\left( U \right).$$
- **A-4:** $D_{{{\text{Chd}},p}} \left( {P,P^{C} } \right) = 0$ if and only if $\mu_{P} \left( {x_{i} } \right) = \nu_{P} \left( {x_{i} } \right),\;\forall \;x_{i} \in U,\;P \in {\text{PFS}}\left( U \right).$
- **A-5:** $$D_{{{\text{Chd}},p}} \left( {P,Q^{C} } \right) = D_{{{\text{Chd}},p}} \left( {P^{C} ,Q} \right),\;\forall \;P,Q \in {\text{PFS}}\left( U \right).$$

##### Proof

(**A-1**) Let $P = \left( {\mu_{P} \left( {x_{i} } \right),\nu_{P} \left( {x_{i} } \right)} \right)$, $Q = \left( {\mu_{Q} \left( {x_{i} } \right),\nu_{Q} \left( {x_{i} } \right)} \right)$, $R = \left( {\mu_{R} \left( {x_{i} } \right),\nu_{R} \left( {x_{i} } \right)} \right)$, and $P \subseteq Q \subseteq R$. Then $\forall \,x_{i} \in U$ we get,

28 $$0 \le \mu_{P} \left( {x_{i} } \right) \le \mu_{Q} \left( {x_{i} } \right) \le \mu_{R} \left( {x_{i} } \right) \le 1$$

29 $${\text{and}}\;0 \le \nu_{R} \left( {x_{i} } \right) \le \nu_{Q} \left( {x_{i} } \right) \le \nu_{P} \left( {x_{i} } \right) \le 1$$

which implies that, $\forall \,x_{i} \in U$

30 $$\left| {\mu_{R} \left( {x_{i} } \right) - \mu_{P} \left( {x_{i} } \right)} \right| \ge \left| {\mu_{Q} \left( {x_{i} } \right) - \mu_{R} \left( {x_{i} } \right)} \right|$$

31 $$\left| {\nu_{R} \left( {x_{i} } \right) - \nu_{P} \left( {x_{i} } \right)} \right| \ge \left| {\nu_{P} \left( {x_{i} } \right) - \nu_{Q} \left( {x_{i} } \right)} \right|$$

32 $$\frac{1}{{\sqrt {1 + \mu_{P}^{2} \left( {x_{i} } \right)} }} \ge \frac{1}{{\sqrt {1 + \mu_{Q}^{2} \left( {x_{i} } \right)} }} \ge \frac{1}{{\sqrt {1 + \mu_{R}^{2} \left( {x_{i} } \right)} }}$$

33 $$\frac{1}{{\sqrt {1 + \nu_{R}^{2} \left( {x_{i} } \right)} }} \ge \frac{1}{{\sqrt {1 + \nu_{Q}^{2} \left( {x_{i} } \right)} }} \ge \frac{1}{{\sqrt {1 + \nu_{P}^{2} \left( {x_{i} } \right)} }}$$

From Eqs. (32) and (33), we have

34 $$\frac{1}{{\sqrt {1 + \mu_{P}^{2} \left( {x_{i} } \right)} \sqrt {1 + \mu_{R}^{2} \left( {x_{i} } \right)} }} \ge \frac{1}{{\sqrt {1 + \mu_{Q}^{2} \left( {x_{i} } \right)} \sqrt {1 + \mu_{R}^{2} \left( {x_{i} } \right)} }}$$

35 $$\frac{1}{{\sqrt {1 + \nu_{P}^{2} \left( {x_{i} } \right)} \sqrt {1 + \nu_{R}^{2} \left( {x_{i} } \right)} }} \ge \frac{1}{{\sqrt {1 + \nu_{P}^{2} \left( {x_{i} } \right)} \sqrt {1 + \nu_{Q}^{2} \left( {x_{i} } \right)} }}$$

From Eqs. (30) & (34), (31) & (35), we obtain

36 $$\frac{{\left| {\mu_{P} \left( {x_{i} } \right) - \mu_{R} \left( {x_{i} } \right)} \right|}}{{\sqrt {1 + \mu_{P}^{2} \left( {x_{i} } \right)} \sqrt {1 + \mu_{R}^{2} \left( {x_{i} } \right)} }} \ge \frac{{\left| {\mu_{Q} \left( {x_{i} } \right) - \mu_{R} \left( {x_{i} } \right)} \right|}}{{\sqrt {1 + \mu_{Q}^{2} \left( {x_{i} } \right)} \sqrt {1 + \mu_{R}^{2} \left( {x_{i} } \right)} }}$$

37 $${\text{and}}\;\frac{{\left| {\nu_{P} \left( {x_{i} } \right) - \nu_{R} \left( {x_{i} } \right)} \right|}}{{\sqrt {1 + \nu_{P}^{2} \left( {x_{i} } \right)} \sqrt {1 + \nu_{R}^{2} \left( {x_{i} } \right)} }} \ge \frac{{\left| {\nu_{P} \left( {x_{i} } \right) - \nu_{Q} \left( {x_{i} } \right)} \right|}}{{\sqrt {1 + \nu_{P}^{2} \left( {x_{i} } \right)} \sqrt {1 + \nu_{Q}^{2} \left( {x_{i} } \right)} }}$$

Using Corollary 1, Eqs. (36) and (37) can also be written as,

38 $$\frac{{\left\| {\mu_{P} \left( {x_{i} } \right) - \mu_{R} \left( {x_{i} } \right)} \right\|}}{{\sqrt {1 + \left\| {\mu_{P} \left( {x_{i} } \right)} \right\|^{2} } \sqrt {1 + \left\| {\mu_{R} \left( {x_{i} } \right)} \right\|^{2} } }} \ge \frac{{\left\| {\mu_{Q} \left( {x_{i} } \right) - \mu_{R} \left( {x_{i} } \right)} \right\|}}{{\sqrt {1 + \left\| {\mu_{Q} \left( {x_{i} } \right)} \right\|^{2} } \sqrt {1 + \left\| {\mu_{R} \left( {x_{i} } \right)} \right\|^{2} } }}$$

39 $$\frac{{\left\| {\nu_{P} \left( {x_{i} } \right) - \nu_{R} \left( {x_{i} } \right)} \right\|}}{{\sqrt {1 + \left\| {\nu_{P} \left( {x_{i} } \right)} \right\|^{2} } \sqrt {1 + \left\| {\nu_{R} \left( {x_{i} } \right)} \right\|^{2} } }} \ge \frac{{\left\| {\nu_{P} \left( {x_{i} } \right) - \nu_{Q} \left( {x_{i} } \right)} \right\|}}{{\sqrt {1 + \left\| {\nu_{P} \left( {x_{i} } \right)} \right\|^{2} } \sqrt {1 + \left\| {\nu_{Q} \left( {x_{i} } \right)} \right\|^{2} } }}$$

From Eq. (28), using Lemma 1, we get $\forall \,x_{i} \in U$,

$$\left| {\mu_{P} \left( {x_{i} } \right) - \mu_{R} \left( {x_{i} } \right)} \right|\sqrt {1 + \mu_{Q}^{2} \left( {x_{i} } \right)} \ge \left| {\mu_{P} \left( {x_{i} } \right) - \mu_{Q} \left( {x_{i} } \right)} \right|\sqrt {1 + \mu_{R}^{2} \left( {x_{i} } \right)}$$

Dividing both sides by $\sqrt {1 + \mu_{P}^{2} \left( {x_{i} } \right)} \sqrt {1 + \mu_{Q}^{2} \left( {x_{i} } \right)} \sqrt {1 + \mu_{R}^{2} \left( {x_{i} } \right)}$ we get,

40 $$\frac{{\left| {\mu_{P} \left( {x_{i} } \right) - \mu_{R} \left( {x_{i} } \right)} \right|}}{{\sqrt {1 + \mu_{P}^{2} \left( {x_{i} } \right)} \sqrt {1 + \mu_{R}^{2} \left( {x_{i} } \right)} }} \ge \frac{{\left| {\mu_{P} \left( {x_{i} } \right) - \mu_{Q} \left( {x_{i} } \right)} \right|}}{{\sqrt {1 + \mu_{P}^{2} \left( {x_{i} } \right)} \sqrt {1 + \mu_{Q}^{2} \left( {x_{i} } \right)} }}$$

By Corollary 1, Eq. (40) can be written as

41 $$\frac{{\left\| {\mu_{P} \left( {x_{i} } \right) - \mu_{R} \left( {x_{i} } \right)} \right\|}}{{\sqrt {1 + \left\| {\mu_{P} \left( {x_{i} } \right)} \right\|^{2} } \sqrt {1 + \left\| {\mu_{R} \left( {x_{i} } \right)} \right\|^{2} } }} \ge \frac{{\left\| {\mu_{P} \left( {x_{i} } \right) - \mu_{Q} \left( {x_{i} } \right)} \right\|}}{{\sqrt {1 + \left\| {\mu_{P} \left( {x_{i} } \right)} \right\|^{2} } \sqrt {1 + \left\| {\mu_{Q} \left( {x_{i} } \right)} \right\|^{2} } }}$$

From Eq. (29), using Lemma 1, we get $\forall \,x_{i} \in U$,

$$\left| {\nu_{R} \left( {x_{i} } \right) - \nu_{P} \left( {x_{i} } \right)} \right|\sqrt {1 + \nu_{Q}^{2} \left( {x_{i} } \right)} \ge \left| {\nu_{R} \left( {x_{i} } \right) - \nu_{Q} \left( {x_{i} } \right)} \right|\sqrt {1 + \nu_{P}^{2} \left( {x_{i} } \right)}$$

Dividing both sides by $\sqrt {1 + \nu_{P}^{2} \left( {x_{i} } \right)} \sqrt {1 + \nu_{Q}^{2} \left( {x_{i} } \right)} \sqrt {1 + \nu_{R}^{2} \left( {x_{i} } \right)}$ we get,

42 $$\frac{{\left| {\nu_{P} \left( {x_{i} } \right) - \nu_{R} \left( {x_{i} } \right)} \right|}}{{\sqrt {1 + \nu_{P}^{2} \left( {x_{i} } \right)} \sqrt {1 + \nu_{R}^{2} \left( {x_{i} } \right)} }} \ge \frac{{\left| {\nu_{Q} \left( {x_{i} } \right) - \nu_{R} \left( {x_{i} } \right)} \right|}}{{\sqrt {1 + \nu_{Q}^{2} \left( {x_{i} } \right)} \sqrt {1 + \nu_{R}^{2} \left( {x_{i} } \right)} }}$$

By Corollary 1, Eq. (42) can be written as

43 $$\frac{{\left\| {\nu_{P} \left( {x_{i} } \right) - \nu_{R} \left( {x_{i} } \right)} \right\|}}{{\sqrt {1 + \left\| {\nu_{P} \left( {x_{i} } \right)} \right\|^{2} } \sqrt {1 + \left\| {\nu_{R} \left( {x_{i} } \right)} \right\|^{2} } }} \ge \frac{{\left\| {\nu_{Q} \left( {x_{i} } \right) - \nu_{R} \left( {x_{i} } \right)} \right\|}}{{\sqrt {1 + \left\| {\nu_{Q} \left( {x_{i} } \right)} \right\|^{2} } \sqrt {1 + \left\| {\nu_{R} \left( {x_{i} } \right)} \right\|^{2} } }}$$

From Eqs. (38) and (43), we get $D_{{{\text{Chd}},p}} \left( {Q,R} \right) \le D_{{{\text{Chd}},p}} \left( {P,R} \right)$.

From Eqs. (39) and (41), we get $D_{{{\text{Chd}},p}} \left( {P,Q} \right) \le D_{{{\text{Chd}},p}} \left( {P,R} \right)$.

This completes the proof.

(**A-2**) We know that, for given $P = \left( {\mu_{P} \left( {x_{i} } \right),\nu_{P} \left( {x_{i} } \right)} \right)$, $P^{C} = \left( {\nu_{P} \left( {x_{i} } \right),\mu_{P} \left( {x_{i} } \right)} \right)$.

Let $$D_{{{\text{Chd}},p}} \left( {P,P^{C} } \right) = 1$$, then

$$\left( {\frac{1}{{2^{{\left( {1 - \tfrac{p}{2}} \right)}} n}}\,\,\sum\limits_{i = 1}^{n} {\left[ {\left\{ {\frac{{\left\| {\mu_{P} \left( {x_{i} } \right) - \nu_{P} \left( {x_{i} } \right)} \right\|}}{{\sqrt {1 + \left\| {\mu_{P} \left( {x_{i} } \right)} \right\|^{2} } \sqrt {1 + \left\| {\mu_{Q} \left( {x_{i} } \right)} \right\|^{2} } }}} \right\}^{p} + \left\{ {\frac{{\left\| {\nu_{P} \left( {x_{i} } \right) - \mu_{P} \left( {x_{i} } \right)} \right\|}}{{\sqrt {1 + \left\| {\nu_{P} \left( {x_{i} } \right)} \right\|^{2} } \sqrt {1 + \left\| {\nu_{Q} \left( {x_{i} } \right)} \right\|^{2} } }}} \right\}^{p} } \right]} } \right)^{{{\raise0.7ex\hbox{$1$} \!\mathord{\left/ {\vphantom {1 p}}\right.\kern-0pt} \!\lower0.7ex\hbox{$p$}}}} = 1$$

Putting $p = 1,\,\,n = 1$, we have

$$\begin{aligned} & \sum\limits_{i = 1}^{n} {\left[ {2\left( {\frac{{\left\| {\mu_{P} \left( {x_{i} } \right) - \nu_{P} \left( {x_{i} } \right)} \right\|}}{{\sqrt {1 + \left\| {\mu_{P} \left( {x_{i} } \right)} \right\|^{2} } \sqrt {1 + \left\| {\mu_{Q} \left( {x_{i} } \right)} \right\|^{2} } }}} \right)} \right]} = \sqrt 2 \\ & \Leftrightarrow 2\left( {\frac{{\left\| {\mu_{P} \left( {x_{i} } \right) - \nu_{P} \left( {x_{i} } \right)} \right\|}}{{\sqrt {1 + \left\| {\mu_{P} \left( {x_{i} } \right)} \right\|^{2} } \sqrt {1 + \left\| {\mu_{Q} \left( {x_{i} } \right)} \right\|^{2} } }}} \right) = \sqrt 2 \\ & \Leftrightarrow \sqrt 2 \left\| {\mu_{P} \left( {x_{i} } \right) - \nu_{P} \left( {x_{i} } \right)} \right\| = \sqrt {1 + \left\| {\mu_{P} \left( {x_{i} } \right)} \right\|^{2} } \sqrt {1 + \left\| {\mu_{Q} \left( {x_{i} } \right)} \right\|^{2} } \\ \end{aligned}$$

By using Corollary 1, we can write the above equation as

$$\Leftrightarrow \sqrt 2 \left( {\mu_{P} \left( {x_{i} } \right) - \nu_{P} \left( {x_{i} } \right)} \right) = \sqrt {1 + \mu_{P}^{2} \left( {x_{i} } \right)} \sqrt {1 + \mu_{Q}^{2} \left( {x_{i} } \right)}$$

Squaring both sides, we have

$$\begin{aligned} & \Leftrightarrow 2\left( {\mu_{P}^{2} \left( {x_{i} } \right) + \nu_{P}^{2} \left( {x_{i} } \right) - 2\mu_{P} \left( {x_{i} } \right)\nu_{P} \left( {x_{i} } \right)} \right) = 1 + \mu_{P}^{2} \left( {x_{i} } \right) + \nu_{P}^{2} \left( {x_{i} } \right) + \mu_{P}^{2} \left( {x_{i} } \right)\nu_{P}^{2} \left( {x_{i} } \right) \\ & \Leftrightarrow \mu_{P}^{2} \left( {x_{i} } \right) + \nu_{P}^{2} \left( {x_{i} } \right) = 1 + 4\mu_{P} \left( {x_{i} } \right)\nu_{P} \left( {x_{i} } \right) + \mu_{P}^{2} \left( {x_{i} } \right)\nu_{P}^{2} \left( {x_{i} } \right) \\ & \Leftrightarrow \mu_{P}^{2} \left( {x_{i} } \right) + \nu_{P}^{2} \left( {x_{i} } \right) - 2\mu_{P} \left( {x_{i} } \right)\nu_{P} \left( {x_{i} } \right) = 1 + 2\mu_{P} \left( {x_{i} } \right)\nu_{P} \left( {x_{i} } \right) + \mu_{P}^{2} \left( {x_{i} } \right)\nu_{P}^{2} \left( {x_{i} } \right) \\ & \Leftrightarrow \left| {\mu_{P} \left( {x_{i} } \right) - \nu_{P} \left( {x_{i} } \right)} \right|^{2} = \left( {1 + \mu_{P} \left( {x_{i} } \right)\nu_{P} \left( {x_{i} } \right)} \right)^{2} \;\left( {{\text{Using}}\;{\text{Corollary}}\;1} \right) \\ & \Leftrightarrow \left| {\mu_{P} \left( {x_{i} } \right) - \nu_{P} \left( {x_{i} } \right)} \right| = 1 + \mu_{P} \left( {x_{i} } \right)\nu_{P} \left( {x_{i} } \right) \\ \end{aligned}$$

Since $$0 \le \left| {\mu_{P} \left( {x_{i} } \right) - \nu_{P} \left( {x_{i} } \right)} \right| \le 1$$ and $$0 \le \mu_{P} \left( {x_{i} } \right),\nu_{P} \left( {x_{i} } \right) \le 1$$, $$\forall \,x_{i} \in U$$

$$\begin{aligned} & \Leftrightarrow \mu_{P} \left( {x_{i} } \right)\nu_{P} \left( {x_{i} } \right) = 0\;{\text{and}}\;\left| {\mu_{P} \left( {x_{i} } \right) - \nu_{P} \left( {x_{i} } \right)} \right| = 1 \\ & \Leftrightarrow \left( {\mu_{P} \left( {x_{i} } \right) = 1\;{\text{and}}\;\nu_{P} \left( {x_{i} } \right) = 0} \right)\;or\;\left( {\mu_{P} \left( {x_{i} } \right) = 0\;{\text{and}}\;\nu_{P} \left( {x_{i} } \right) = 1} \right) \\ & \Leftrightarrow P = \left( {1,0} \right)\;{\text{or}}\;P = \left( {0,1} \right) \\ & \Leftrightarrow P\;{\text{is}}\;{\text{a}}\;{\text{crisp}}\;{\text{set}}. \\ \end{aligned}$$

(**A-3**) We know that, for given $P = \left( {\mu_{P} \left( {x_{i} } \right),\nu_{P} \left( {x_{i} } \right)} \right)$ and $Q = \left( {\mu_{Q} \left( {x_{i} } \right),\nu_{Q} \left( {x_{i} } \right)} \right)$, we have their complements as $P^{C} = \left( {\nu_{P} \left( {x_{i} } \right),\mu_{P} \left( {x_{i} } \right)} \right)$ and $Q^{C} = \left( {\nu_{Q} \left( {x_{i} } \right),\mu_{Q} \left( {x_{i} } \right)} \right)$.

$$\begin{aligned} D_{{{\text{Chd}},p}} \left( {P,Q} \right) & = \left( {\frac{1}{{2^{{\left( {1 - \tfrac{p}{2}} \right)}} n}}\sum\limits_{i = 1}^{n} {\left[ {\left\{ {\frac{{\left\| {\mu_{P} \left( {x_{i} } \right) - \mu_{Q} \left( {x_{i} } \right)} \right\|}}{{\sqrt {1 + \left\| {\mu_{P} \left( {x_{i} } \right)} \right\|^{2} } \sqrt {1 + \left\| {\mu_{Q} \left( {x_{i} } \right)} \right\|^{2} } }}} \right\}^{p} + \left\{ {\frac{{\left\| {\nu_{P} \left( {x_{i} } \right) - \nu_{Q} \left( {x_{i} } \right)} \right\|}}{{\sqrt {1 + \left\| {\nu_{P} \left( {x_{i} } \right)} \right\|^{2} } \sqrt {1 + \left\| {\nu_{Q} \left( {x_{i} } \right)} \right\|^{2} } }}} \right\}^{p} } \right]} } \right)^{{{\raise0.7ex\hbox{$1$} \!\mathord{\left/ {\vphantom {1 p}}\right.\kern-0pt} \!\lower0.7ex\hbox{$p$}}}} \\ & = \left( {\frac{1}{{2^{{\left( {1 - \tfrac{p}{2}} \right)}} n}}\,\,\sum\limits_{i = 1}^{n} {\left[ {\left\{ {\frac{{\left\| {\nu_{P} \left( {x_{i} } \right) - \nu_{Q} \left( {x_{i} } \right)} \right\|}}{{\sqrt {1 + \left\| {\nu_{P} \left( {x_{i} } \right)} \right\|^{2} } \sqrt {1 + \left\| {\nu_{Q} \left( {x_{i} } \right)} \right\|^{2} } }}} \right\}^{p} + \left\{ {\frac{{\left\| {\mu_{P} \left( {x_{i} } \right) - \mu_{Q} \left( {x_{i} } \right)} \right\|}}{{\sqrt {1 + \left\| {\mu_{P} \left( {x_{i} } \right)} \right\|^{2} } \sqrt {1 + \left\| {\mu_{Q} \left( {x_{i} } \right)} \right\|^{2} } }}} \right\}^{p} } \right]} } \right)^{{{\raise0.7ex\hbox{$1$} \!\mathord{\left/ {\vphantom {1 p}}\right.\kern-0pt} \!\lower0.7ex\hbox{$p$}}}} \\ & = D_{{{\text{Chd}},p}} \left( {P^{C} ,Q^{C} } \right) \\ \end{aligned}$$

This completes the proof.

(**A-4**) Let $$D_{{{\text{Chd}},p}} \left( {P,P^{C} } \right) = 0$$, then $$\forall \,x_{i} \in U$$,

$$\begin{aligned} & \frac{1}{{2^{{\left( {1 - \tfrac{p}{2}} \right)}} n}}\sum\limits_{i = 1}^{n} {\left[ {2\left\{ {\frac{{\left\| {\mu_{P} \left( {x_{i} } \right) - \nu_{P} \left( {x_{i} } \right)} \right\|}}{{\sqrt {1 + \left\| {\mu_{P} \left( {x_{i} } \right)} \right\|^{2} } \sqrt {1 + \left\| {\mu_{Q} \left( {x_{i} } \right)} \right\|^{2} } }}} \right\}^{p} } \right]} = 0 \\ & \Leftrightarrow \left( {\frac{{\left\| {\mu_{P} \left( {x_{i} } \right) - \nu_{P} \left( {x_{i} } \right)} \right\|}}{{\sqrt {1 + \left\| {\mu_{P} \left( {x_{i} } \right)} \right\|^{2} } \sqrt {1 + \left\| {\mu_{Q} \left( {x_{i} } \right)} \right\|^{2} } }}} \right)^{p} = 0,\;\forall \;x_{i} \in U,\;\forall \;p \in {\mathbb{R}}^{ + } - \left\{ 0 \right\} \\ & \Leftrightarrow \left\| {\mu_{P} \left( {x_{i} } \right) - \nu_{P} \left( {x_{i} } \right)} \right\| = 0 \\ & \Leftrightarrow \mu_{P} \left( {x_{i} } \right) = \nu_{P} \left( {x_{i} } \right),\;\forall x_{i} \in U \\ \end{aligned}$$

(**A-5**) For given $P = \left( {\mu_{P} \left( {x_{i} } \right),\nu_{P} \left( {x_{i} } \right)} \right)$ and $Q = \left( {\mu_{Q} \left( {x_{i} } \right),\nu_{Q} \left( {x_{i} } \right)} \right)$, we have their complements as $P^{C} = \left( {\nu_{P} \left( {x_{i} } \right),\mu_{P} \left( {x_{i} } \right)} \right)$ and $Q^{C} = \left( {\nu_{Q} \left( {x_{i} } \right),\mu_{Q} \left( {x_{i} } \right)} \right)$. Thus,

$$\begin{aligned} D_{{{\text{Chd}},p}} \left( {P,Q^{C} } \right) & = \left( {\frac{1}{{2^{{\left( {1 - \tfrac{p}{2}} \right)}} n}}\sum\limits_{i = 1}^{n} {\left[ {\left\{ {\frac{{\left\| {\mu_{P} \left( {x_{i} } \right) - \nu_{Q} \left( {x_{i} } \right)} \right\|}}{{\sqrt {1 + \left\| {\mu_{P} \left( {x_{i} } \right)} \right\|^{2} } \sqrt {1 + \left\| {\nu_{Q} \left( {x_{i} } \right)} \right\|^{2} } }}} \right\}^{p} + \left\{ {\frac{{\left\| {\nu_{P} \left( {x_{i} } \right) - \mu_{Q} \left( {x_{i} } \right)} \right\|}}{{\sqrt {1 + \left\| {\nu_{P} \left( {x_{i} } \right)} \right\|^{2} } \sqrt {1 + \left\| {\mu_{Q} \left( {x_{i} } \right)} \right\|^{2} } }}} \right\}^{p} } \right]} } \right)^{{{\raise0.7ex\hbox{$1$} \!\mathord{\left/ {\vphantom {1 p}}\right.\kern-0pt} \!\lower0.7ex\hbox{$p$}}}} \\ & = \left( {\frac{1}{{2^{{\left( {1 - \tfrac{p}{2}} \right)}} n}}\sum\limits_{i = 1}^{n} {\left[ {\left\{ {\frac{{\left\| {\nu_{P} \left( {x_{i} } \right) - \mu_{Q} \left( {x_{i} } \right)} \right\|}}{{\sqrt {1 + \left\| {\nu_{P} \left( {x_{i} } \right)} \right\|^{2} } \sqrt {1 + \left\| {\mu_{Q} \left( {x_{i} } \right)} \right\|^{2} } }}} \right\}^{p} + \left\{ {\frac{{\left\| {\mu_{P} \left( {x_{i} } \right) - \nu_{Q} \left( {x_{i} } \right)} \right\|}}{{\sqrt {1 + \left\| {\mu_{P} \left( {x_{i} } \right)} \right\|^{2} } \sqrt {1 + \left\| {\nu_{Q} \left( {x_{i} } \right)} \right\|^{2} } }}} \right\}^{p} } \right]} } \right)^{{{\raise0.7ex\hbox{$1$} \!\mathord{\left/ {\vphantom {1 p}}\right.\kern-0pt} \!\lower0.7ex\hbox{$p$}}}} \\ & = D_{{{\text{Chd}},p}} \left( {P^{C} ,Q} \right) \\ \end{aligned}$$

This completes the proof.

Thus, the proofs of various theorems and lemmas as shown above, suggests that our proposed distance function is worthy of being called as a “distance measure”.

**Fig. 1:**
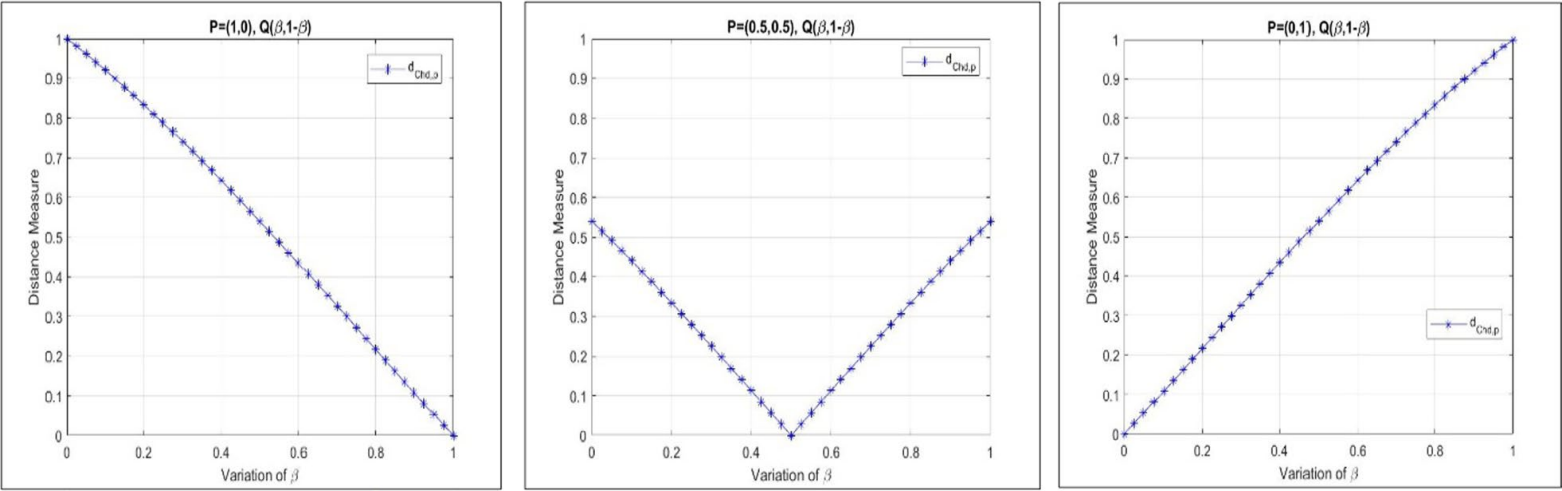
Nonlinear characteristics of the proposed generalized chordal distance measure

The nonlinear nature of our proposed generalized chordal distance for PFSs is demonstrated with the help of the following graphical illustration as shown in Fig. 1.

**Fig. 2:**
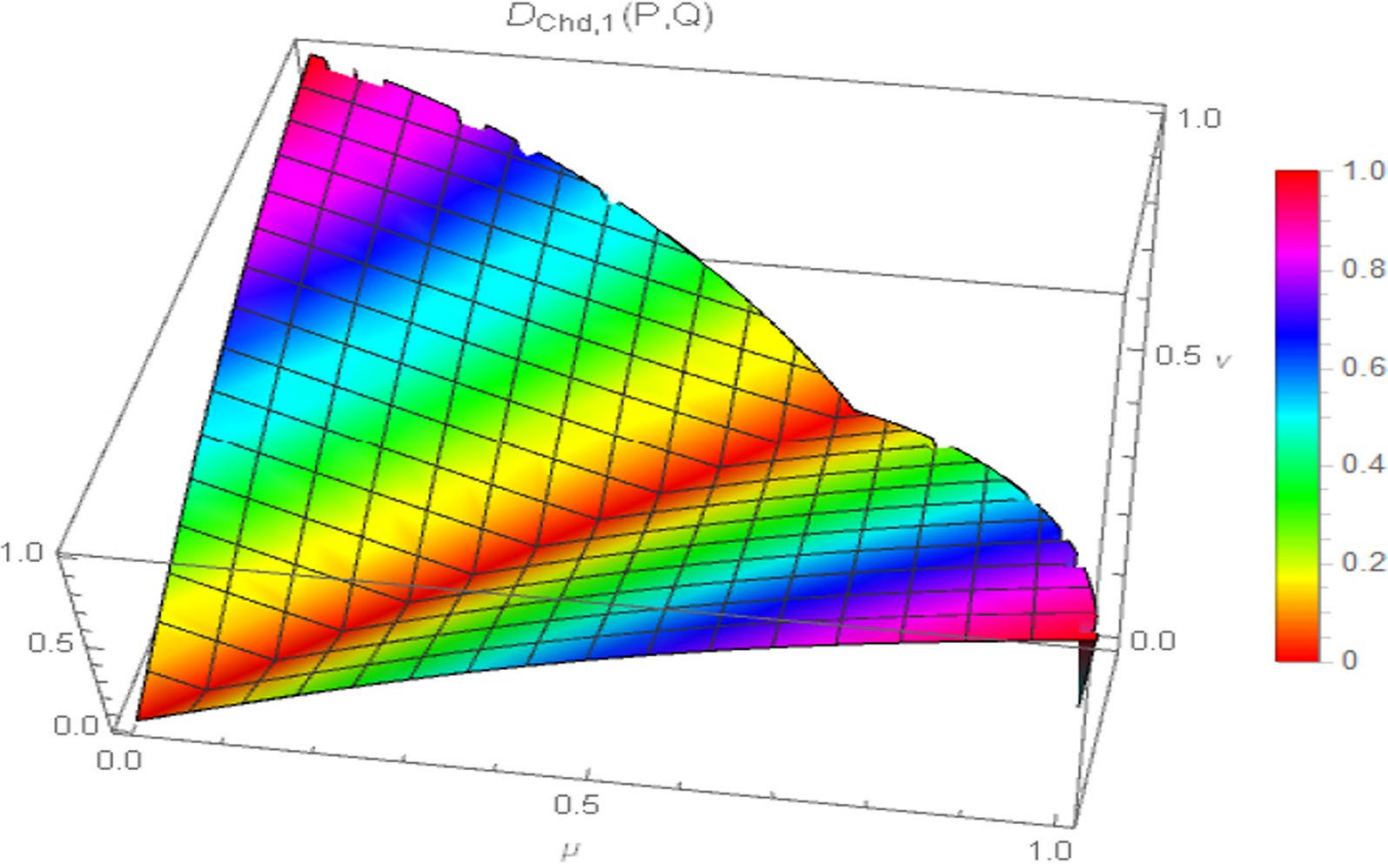
Surface of generalized chordal distance measure for $p = 1$

**Fig. 3:**
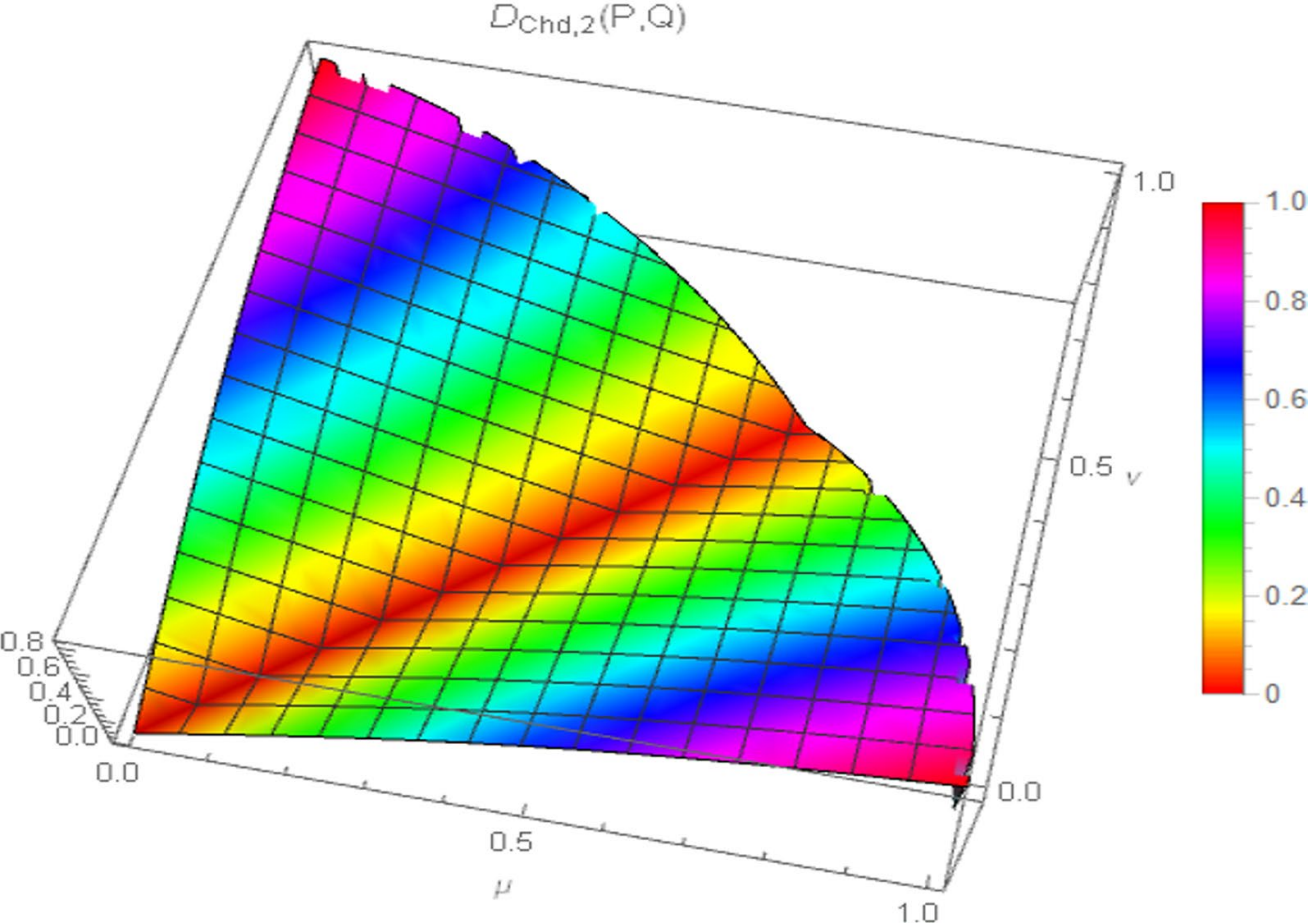
Surface of generalized chordal distance measure for $p = 2$

**Fig. 4:**
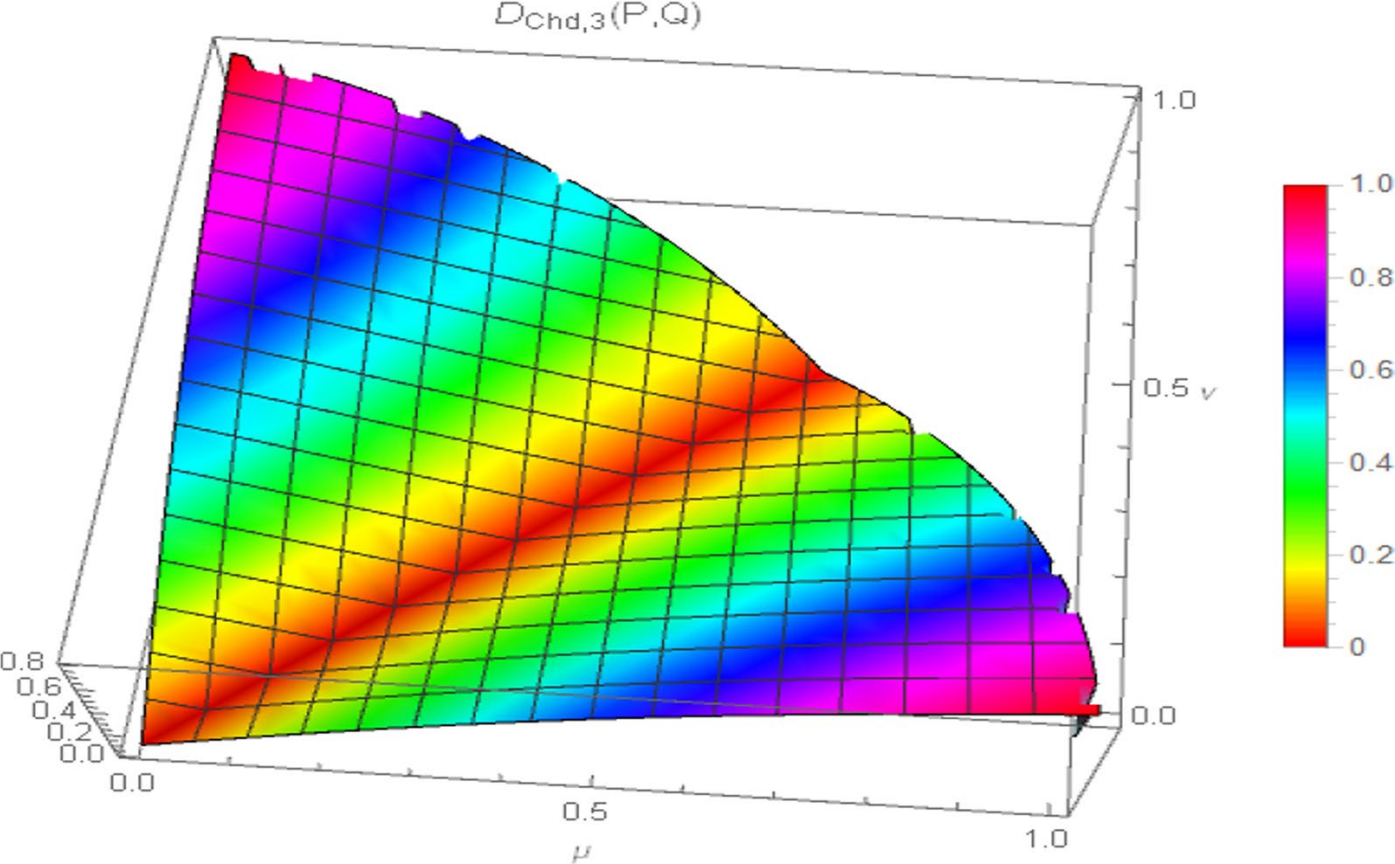
Surface of generalized chordal distance measure for $p = 3$

**Fig. 5:**
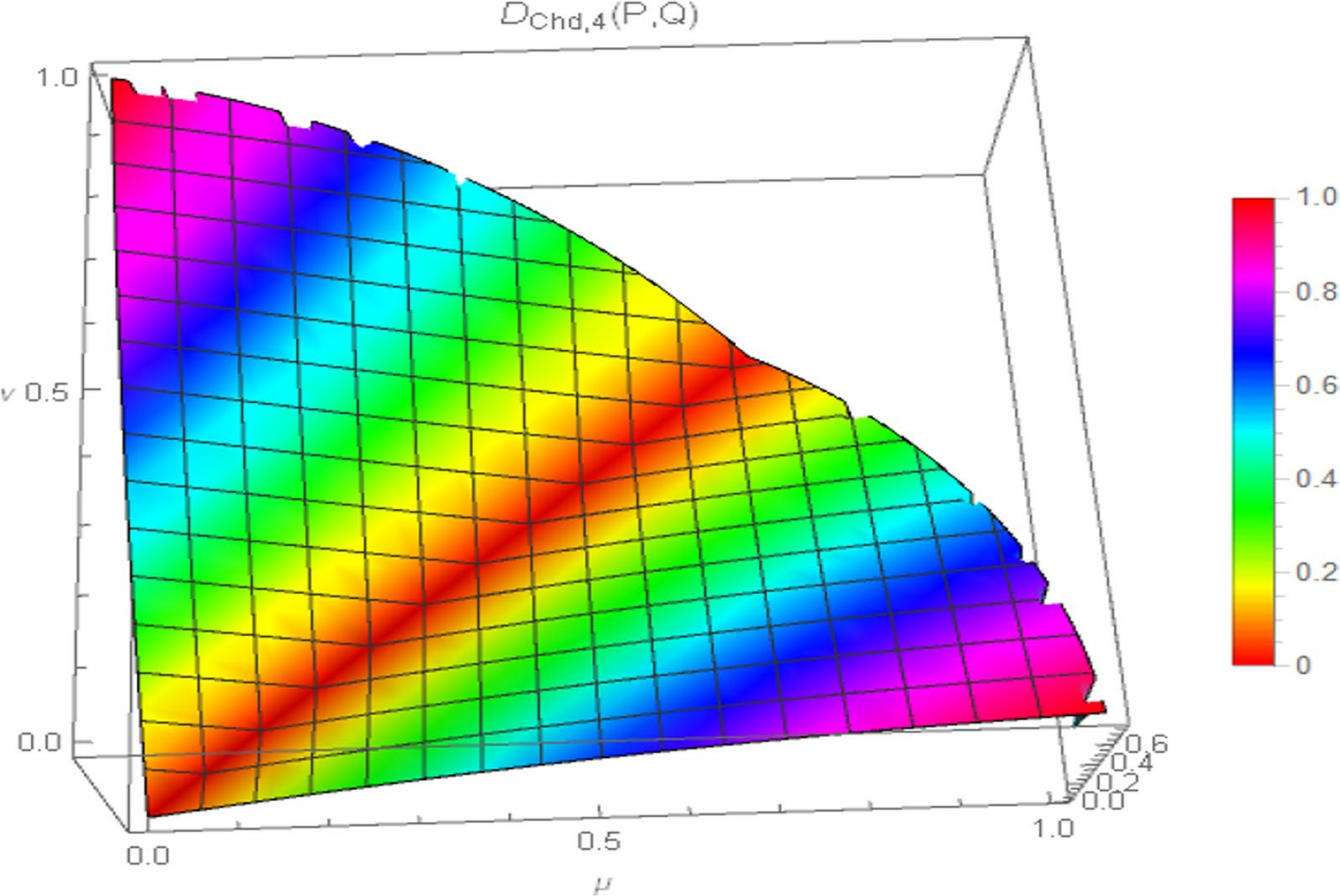
Surface of generalized chordal distance measure for $p = 4$

Moreover, the surface visualization of the generalized chordal distance measure for different values of the input parameter “$p$” ($p = 1,2,3,4$) is depicted in Figs. 2, 3, 4, and 5.

#### Non-Archimedean chordal distance

For any two PFSs, $P = \left( {\mu_{P} \left( {x_{i} } \right),\nu_{P} \left( {x_{i} } \right)} \right)$ and $Q = \left( {\mu_{Q} \left( {x_{i} } \right),\nu_{Q} \left( {x_{i} } \right)} \right)$ defined in the universe of discourse, $U = \left\{ {x_{1} ,x_{2} ,...,x_{n} } \right\}$, the non-Archimedean chordal distance in PFSs is defined as

44 $$D_{{{\text{nAChd}},p}}^{\lambda } \left( {P,Q} \right) = \left( {\sum\limits_{i = 1}^{n} {\left[ {\left\{ {\frac{{D_{{{\text{nAChd}}}}^{\lambda } \left( {\mu_{P} \left( {x_{i} } \right),\mu_{Q} \left( {x_{i} } \right)} \right)}}{20n}} \right\}^{p} + \left\{ {\frac{{D_{{{\text{nAChd}}}}^{\lambda } \left( {\nu_{P} \left( {x_{i} } \right),\nu_{Q} \left( {x_{i} } \right)} \right)}}{20n}} \right\}^{p} } \right]} } \right)^{{{\raise0.7ex\hbox{$1$} \!\mathord{\left/ {\vphantom {1 p}}\right.\kern-0pt} \!\lower0.7ex\hbox{$p$}}}}$$

##### Remark 4

The newly constructed distance $D_{{{\text{nAChd}},p}}^{\lambda }$ satisfies the conditions of a metric, which can be established by adopting the similar procedure used to show $D_{{{\text{Chd}},p}}$ as a distance measure. The proof is not elaborated here to maintain the concise length of the article.

**Fig. 6:**
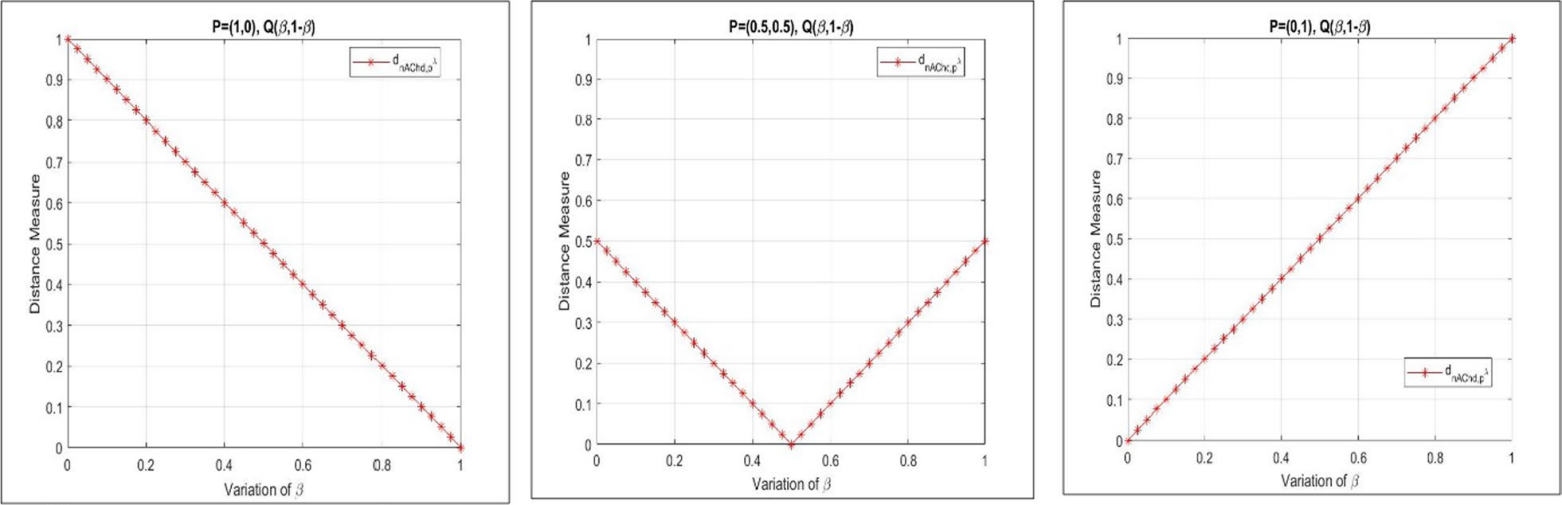
Nonlinear characteristics of the proposed non-Archimedean chordal distance measure

Here, we showcase the nonlinear nature of our proposed non-Archimedean chordal distance for PFSs in Fig. 6 as shown below.

**Fig. 7:**
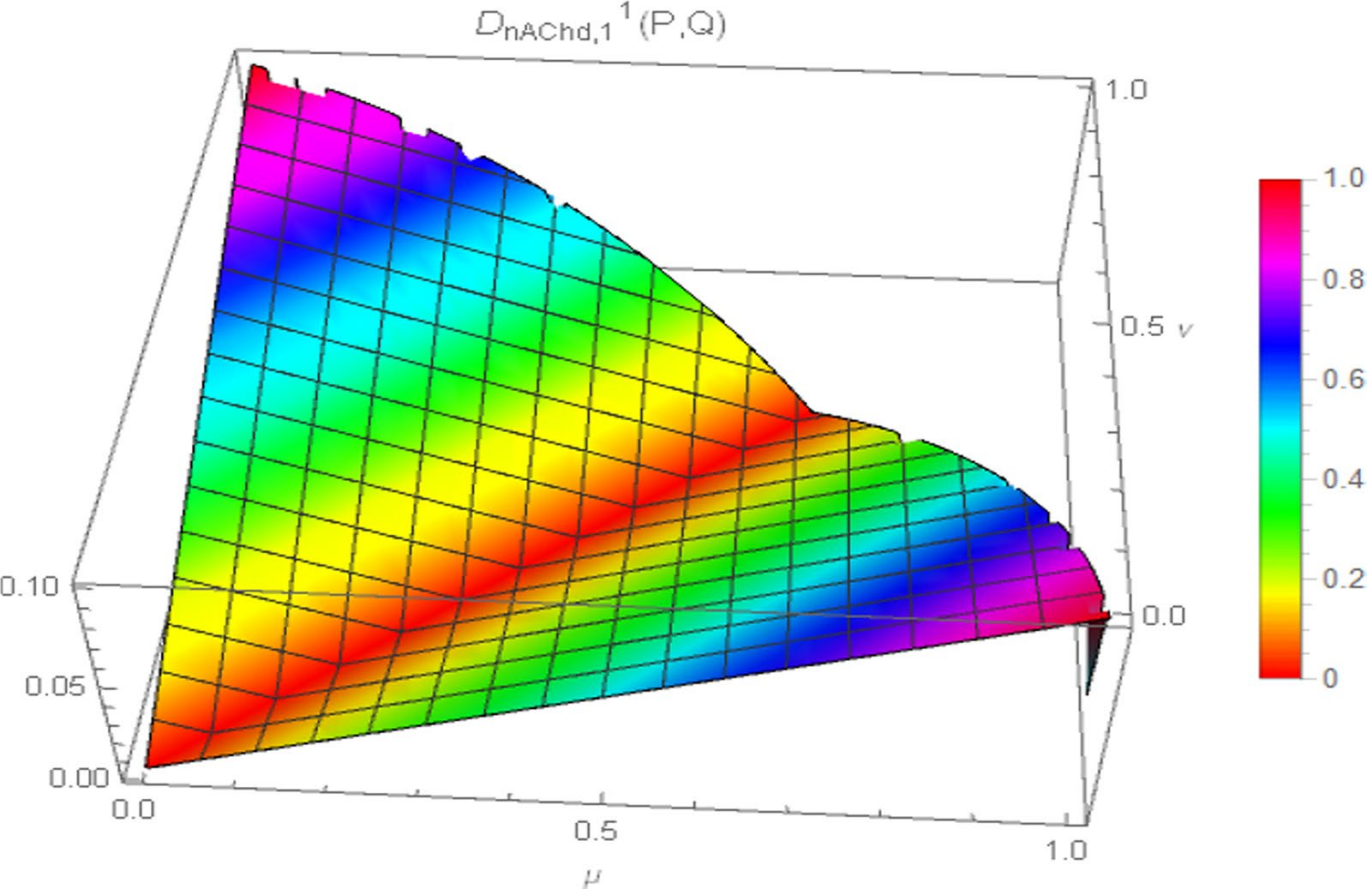
Surface of non-Archimedean chordal distance measure for $\lambda = 1\,;\,p = 1$

**Fig. 8:**
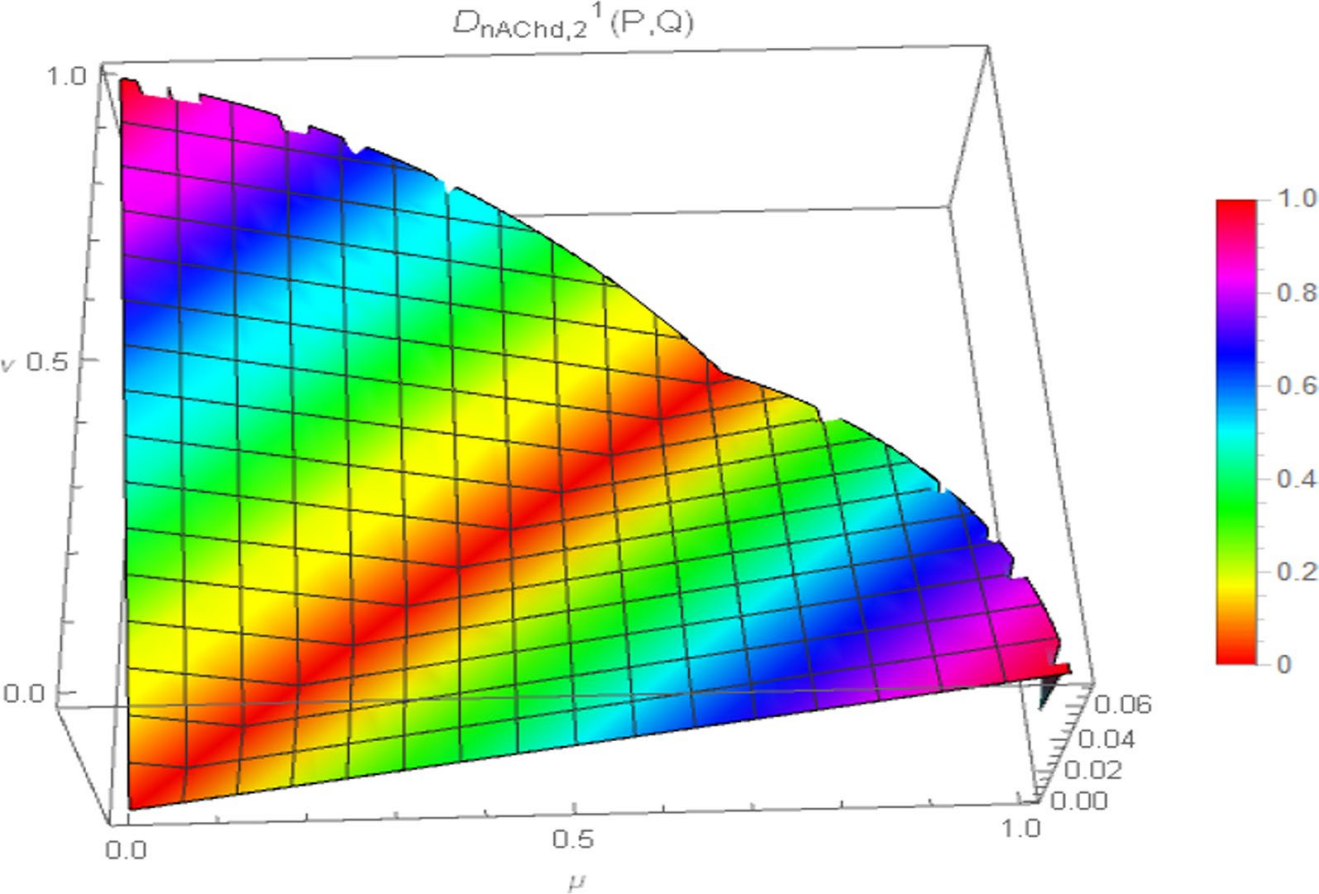
Surface of non-Archimedean chordal distance measure for $\lambda = 1\,;\,p = 2$

**Fig. 9:**
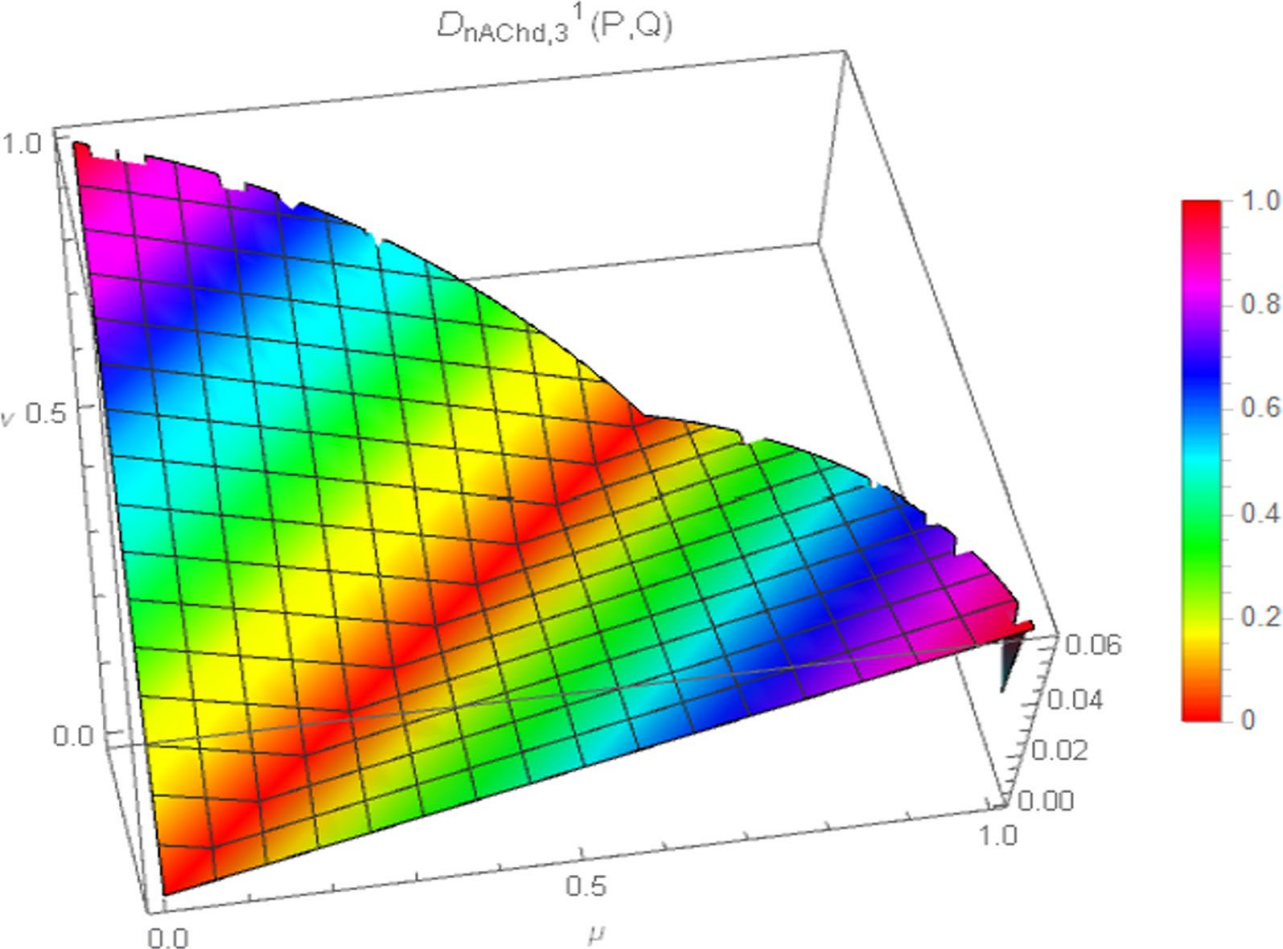
Surface of non-Archimedean chordal distance measure for $\lambda = 1\,;\,p = 3$

**Fig. 10:**
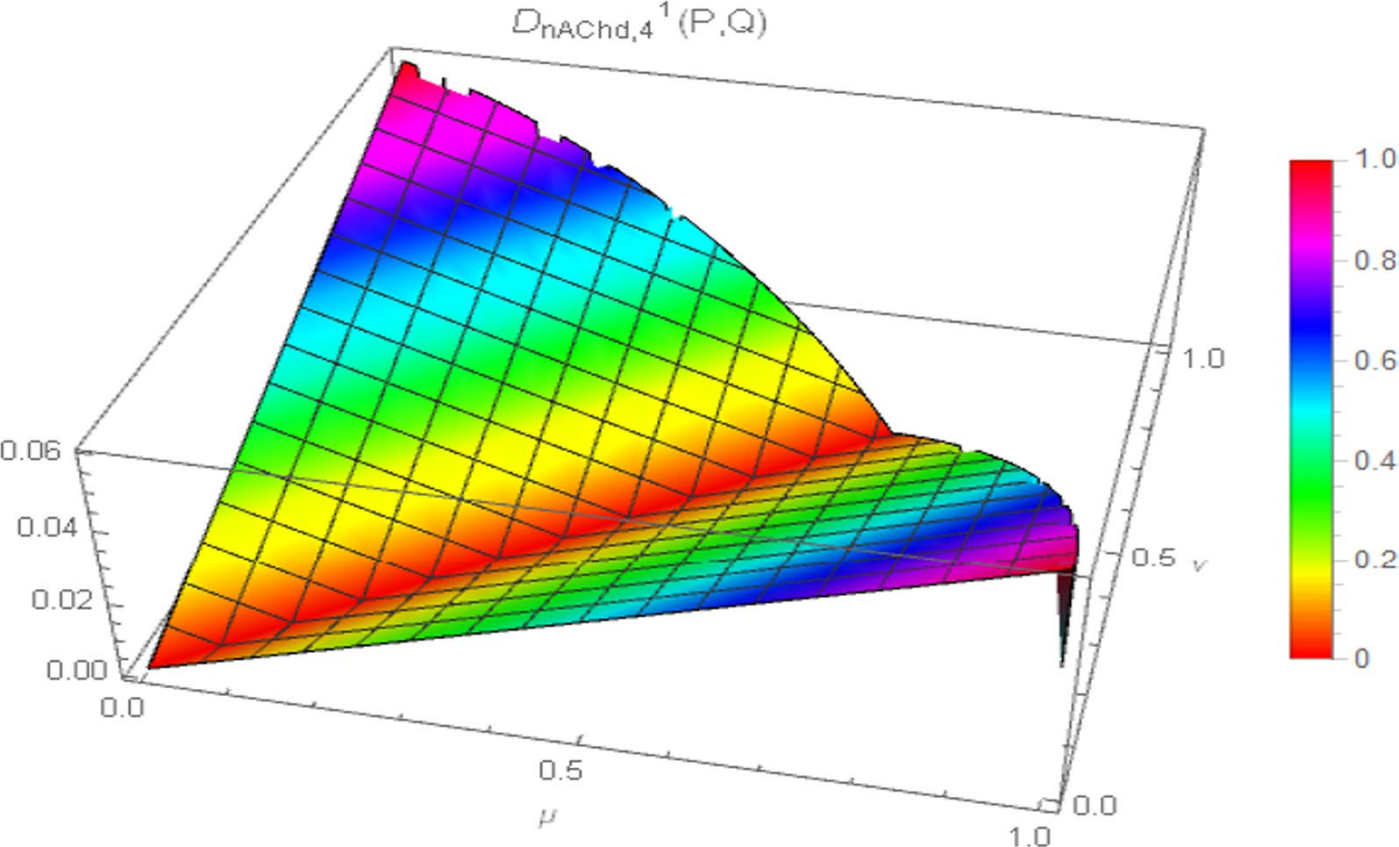
Surface of non-Archimedean chordal distance measure for $\lambda = 1\,;\,p = 4$

Consequently, the 3D plot of the non-Archimedean chordal distance measure for a particular value of $\lambda$, say $\lambda = 1$, and for $p = 1,2,3,4$ are presented in Figs. 7, 8, 9, and 10.

### Propositions

It is found that certain propositions are satisfied by both our newly defined distance measures which are listed below.

#### Proposition 1

For any two PFSs, $P = \left( {1,0} \right)$ and $Q = \left( {0,1} \right)$, we have $D_{Chd,p} \left( {P,Q} \right) = 1$ when $p = 1$; and $D_{{{\text{nAChd}},p}}^{\lambda } \left( {P,Q} \right) = 1$ when $p = 1\,;\,\lambda = 0.1$.

#### Proof

Given $P = \left( {1,0} \right)$ and $Q = \left( {0,1} \right)$. Now, for $p = 1$ the formula for $D_{Chd,1} \left( {P,Q} \right)$ with the given assumptions has the following representation,

45 $$D_{{{\text{Chd}},1}} \left( {P,Q} \right) = \frac{1}{\sqrt 2 } \times \left[ {D_{{{\text{Chd}}}} \left( {\mu_{P} \left( x \right),\mu_{Q} \left( x \right)} \right) + D_{{{\text{Chd}}}} \left( {\nu_{P} \left( x \right),\nu_{Q} \left( x \right)} \right)} \right]$$

and for $p = 1\,;\,\lambda = 0.1$, $D_{{{\text{nAChd}},1}}^{1} \left( {P,Q} \right)$ has the form,

46 $$D_{{{\text{nAChd}},1}}^{0.1} \left( {P,Q} \right) = \left[ {\frac{{D_{{{\text{nAChd}}}}^{0.1} \left( {\mu_{P} \left( x \right),\mu_{Q} \left( x \right)} \right)}}{20} + \frac{{D_{{{\text{nAChd}}}}^{0.1} \left( {\nu_{P} \left( x \right),\nu_{Q} \left( x \right)} \right)}}{20}} \right]$$

Here, $$\mu_{P} \left( x \right) = 1\,,\,\nu_{P} \left( x \right) = 0\,;\,\mu_{Q} \left( x \right) = 0\,,\,\nu_{Q} \left( x \right) = 1$$.

Therefore, we have

$$\begin{aligned} D_{{{\text{Chd}},1}} \left( {P,Q} \right) & = \frac{1}{\sqrt 2 } \times \left[ {D_{{{\text{Chd}}}} \left( {1,0} \right) + D_{{{\text{Chd}}}} \left( {0,1} \right)} \right] \\ \Rightarrow D_{{{\text{Chd}},1}} \left( {\left( {1,0} \right),\left( {0,1} \right)} \right) & = \frac{1}{\sqrt 2 } \times \left[ {\frac{1}{\sqrt 2 \sqrt 1 } + \frac{1}{\sqrt 1 \sqrt 2 }} \right] \\ & = \frac{1}{\sqrt 2 } \times \left[ {\frac{2}{\sqrt 2 }} \right] = \frac{2}{2} = 1 \\ \end{aligned}$$

and, $$\begin{aligned} D_{{{\text{nAChd}},1}}^{0.1} \left( {P,Q} \right) & = \left[ {\frac{{D_{{{\text{nAChd}}}}^{0.1} \left( {1,0} \right)}}{20} + \frac{{D_{{{\text{nAChd}}}}^{0.1} \left( {0,1} \right)}}{20}} \right] \\ \Rightarrow D_{{{\text{nAChd}},1}}^{0.1} \left( {\left( {1,0} \right),\left( {0,1} \right)} \right) & = \left[ {\frac{1}{20} \times \frac{1}{{\max \left\{ {0.1,1} \right\}\max \left\{ {0.1,0} \right\}}} + \frac{1}{20} \times \frac{1}{{\max \left\{ {0.1,0} \right\}\max \left\{ {0.1,1} \right\}}}} \right] \\ & = \frac{1}{20} \times \left[ {\frac{1}{1 \times 0.1} + \frac{1}{0.1 \times 1}} \right] = \frac{1}{20} \times \left[ {10 + 10} \right] = \frac{1}{20} \times 20 = 1 \\ \end{aligned}$$

Hence the result. Noteworthy that, for the pairs $P = \left( {0,1} \right)$ and $Q = \left( {1,0} \right)$, we obtain similar outcomes.

#### Proposition 2

Let us consider two PFSs, $P = \left( {a,b} \right)$ and $Q = \left( {b,a} \right)$, then we have $D_{{{\text{Chd}},p}} \left( {P,Q} \right) = \sqrt 2 \times D_{{{\text{Chd}}}} \left( {a,b} \right)$ when $p = 1$; and $10D_{{{\text{nAChd}},p}}^{\lambda } \left( {P,Q} \right) = {\rm N}(a,b)$ when $p = 1\,;\,\lambda = 1$, for $0 \le a,b < 1$.

#### Proof

Given that,$P = \left( {a,b} \right)$ and $Q = \left( {b,a} \right)$, where $0 \le a,b < 1$. Now, for $p = 1$ we have the formula for $D_{{{\text{Chd}},1}} \left( {P,Q} \right)$ as,

47 $$D_{{{\text{Chd}},1}} \left( {P,Q} \right) = \frac{1}{\sqrt 2 } \times \left[ {D_{{{\text{Chd}}}} \left( {\mu_{P} \left( x \right),\mu_{Q} \left( x \right)} \right) + D_{{{\text{Chd}}}} \left( {\nu_{P} \left( x \right),\nu_{Q} \left( x \right)} \right)} \right]$$

and for $p = 1\,;\,\lambda = 1$, $D_{{{\text{nAChd}},1}}^{1} \left( {P,Q} \right)$ has the following representation,

48 $$D_{{{\text{nAChd}},1}}^{1} \left( {P,Q} \right) = \left[ {\frac{{D_{{{\text{nAChd}}}}^{1} \left( {\mu_{P} \left( x \right),\mu_{Q} \left( x \right)} \right)}}{20} + \frac{{D_{{{\text{nAChd}}}}^{1} \left( {\nu_{P} \left( x \right),\nu_{Q} \left( x \right)} \right)}}{20}} \right]$$

Here, $$\mu_{P} \left( x \right) = a\,,\,\nu_{P} \left( x \right) = b\,;\,\mu_{Q} \left( x \right) = b\,,\,\nu_{Q} \left( x \right) = a$$.

Therefore, we have

$$\begin{aligned} D_{{{\text{Chd}},1}} \left( {P,Q} \right) & = \frac{1}{\sqrt 2 } \times \left[ {D_{{{\text{Chd}}}} \left( {a,b} \right) + D_{{{\text{Chd}}}} \left( {b,a} \right)} \right] \\ \Rightarrow D_{{{\text{Chd}},1}} \left( {\left( {a,b} \right),\left( {b,a} \right)} \right) & = \frac{1}{\sqrt 2 } \times \left[ {\frac{{\left\| {a - b} \right\|}}{{\sqrt {1 + \left\| a \right\|^{2} } \sqrt {1 + \left\| b \right\|^{2} } }} + \frac{{\left\| {b - a} \right\|}}{{\sqrt {1 + \left\| b \right\|^{2} } \sqrt {1 + \left\| a \right\|^{2} } }}} \right] \\ & = \frac{1}{\sqrt 2 } \times \left[ {2 \times \frac{{\left\| {a - b} \right\|}}{{\sqrt {1 + \left\| a \right\|^{2} } \sqrt {1 + \left\| b \right\|^{2} } }}} \right],\;\left( {{\text{since}}\;a,b \ge 0 \Rightarrow \left\| a \right\| = a,\left\| b \right\| = b,\left\| {a - b} \right\| = \left\| {b - a} \right\|} \right) \\ & = \frac{2}{\sqrt 2 } \times \frac{{\left\| {a - b} \right\|}}{{\sqrt {1 + \left\| a \right\|^{2} } \sqrt {1 + \left\| b \right\|^{2} } }} \\ & = \sqrt 2 \times D_{{{\text{Chd}}}} \left( {a,b} \right) \\ \end{aligned}$$

and, $$D_{{{\text{nAChd}},1}}^{1} \left( {P,Q} \right) = \left[ {\frac{{D_{{{\text{nAChd}}}}^{1} \left( {a,b} \right)}}{20} + \frac{{D_{{{\text{nAChd}}}}^{1} \left( {b,a} \right)}}{20}} \right]$$

$$\begin{aligned} \Rightarrow D_{{{\text{nAChd}},1}}^{1} \left( {\left( {a,b} \right),\left( {b,a} \right)} \right) & = \frac{1}{20} \times \left[ {\frac{{\left| {a - b} \right|}}{{\max \left\{ {1,\left| a \right|} \right\}\max \left\{ {1,\left| b \right|} \right\}}} + \frac{{\left| {b - a} \right|}}{{\max \left\{ {1,\left| b \right|} \right\}\max \left\{ {1,\left| a \right|} \right\}}}} \right] \\ & = \frac{1}{20} \times \left[ {2 \times \left| {a - b} \right|} \right],\,\left( {{\text{since}}\;0 \le a,b < 1 \Rightarrow \max \left\{ {1,\left| a \right|} \right\} = \max \left\{ {1,\left| b \right|} \right\} = 1} \right) \\ & = \frac{2}{20} \times {\rm N}\left( {a,b} \right) = \frac{{{\rm N}\left( {a,b} \right)}}{10} \\ \Rightarrow 10D_{{{\text{nAChd}},p}}^{\lambda } \left( {P,Q} \right) & = {\rm N}\left( {a,b} \right) \\ \end{aligned}$$

This completes the proof.

#### Proposition 3

For PFSs, $P = \left( {0,0} \right)$ and $Q = \left( {0,0} \right)$, we have $D_{{{\text{Chd}},p}} \left( {P,Q} \right) = 0$ and $D_{{{\text{nAChd}},p}}^{\lambda } \left( {P,Q} \right) = 0$.

#### Proof

For, $P = \left( {0,0} \right)$ and $Q = \left( {0,0} \right)$, by definition of $D_{{{\text{Chd}},p}} \left( {P,Q} \right)$ and $D_{{{\text{nAChd}},p}}^{\lambda } \left( {P,Q} \right)$ we have,

$$\begin{aligned} D_{{{\text{Chd}},p}} \left( {P,Q} \right) & = \left( {\frac{1}{{2^{{\left( {1 - \tfrac{p}{2}} \right)}} }} \times \left[ {\left\{ {D_{{{\text{Chd}}}} \left( {\mu_{P} \left( {x_{i} } \right),\mu_{Q} \left( {x_{i} } \right)} \right)} \right\}^{p} + \left\{ {D_{{{\text{Chd}}}} \left( {\nu_{P} \left( {x_{i} } \right),\nu_{Q} \left( {x_{i} } \right)} \right)} \right\}^{p} } \right]} \right)^{{{\raise0.7ex\hbox{$1$} \!\mathord{\left/ {\vphantom {1 p}}\right.\kern-0pt} \!\lower0.7ex\hbox{$p$}}}} \\ \Rightarrow D_{{{\text{Chd,}}p}} \left( {\left( {0,0} \right),\left( {0,0} \right)} \right) & = \left( {\frac{1}{{2^{{\left( {1 - \tfrac{p}{2}} \right)}} }} \times \left[ {\left\{ {D_{{{\text{Chd}}}} \left( {\mu_{P} \left( {x_{i} } \right),\mu_{Q} \left( {x_{i} } \right)} \right)} \right\}^{p} + \left\{ {D_{{{\text{Chd}}}} \left( {\nu_{P} \left( {x_{i} } \right),\nu_{Q} \left( {x_{i} } \right)} \right)} \right\}^{p} } \right]} \right)^{{{\raise0.7ex\hbox{$1$} \!\mathord{\left/ {\vphantom {1 p}}\right.\kern-0pt} \!\lower0.7ex\hbox{$p$}}}} \\ & = \left( {\frac{1}{{2^{{\left( {1 - \tfrac{p}{2}} \right)}} }} \times \left[ {\left\{ {D_{{{\text{Chd}}}} \left( {0,0} \right)} \right\}^{p} + \left\{ {D_{{{\text{Chd}}}} \left( {0,0} \right)} \right\}^{p} } \right]} \right)^{{{\raise0.7ex\hbox{$1$} \!\mathord{\left/ {\vphantom {1 p}}\right.\kern-0pt} \!\lower0.7ex\hbox{$p$}}}} \\ & = \left( {\frac{1}{{2^{{\left( {1 - \tfrac{p}{2}} \right)}} }} \times \left[ {\left\{ {\frac{{\left\| {0 - 0} \right\|}}{\sqrt 1 \sqrt 1 }} \right\}^{p} + \left\{ {\frac{{\left\| {0 - 0} \right\|}}{\sqrt 1 \sqrt 1 }} \right\}^{p} } \right]} \right)^{{{\raise0.7ex\hbox{$1$} \!\mathord{\left/ {\vphantom {1 p}}\right.\kern-0pt} \!\lower0.7ex\hbox{$p$}}}} \\ & = \left( {\frac{1}{{2^{{\left( {1 - \tfrac{p}{2}} \right)}} }} \times 0} \right)^{{{\raise0.7ex\hbox{$1$} \!\mathord{\left/ {\vphantom {1 p}}\right.\kern-0pt} \!\lower0.7ex\hbox{$p$}}}} = 0 \\ \end{aligned}$$

and,$$D_{{{\text{nAChd}},p}}^{\lambda } \left( {P,Q} \right) = \left[ {\left\{ {\frac{{D_{{{\text{nAChd}}}}^{\lambda } \left( {\mu_{P} \left( {x_{i} } \right),\mu_{Q} \left( {x_{i} } \right)} \right)}}{20}} \right\}^{p} + \left\{ {\frac{{D_{{{\text{nAChd}}}}^{\lambda } \left( {\nu_{P} \left( {x_{i} } \right),\nu_{Q} \left( {x_{i} } \right)} \right)}}{20}} \right\}^{p} } \right]^{{{\raise0.7ex\hbox{$1$} \!\mathord{\left/ {\vphantom {1 p}}\right.\kern-0pt} \!\lower0.7ex\hbox{$p$}}}}$$

$$\begin{aligned} \Rightarrow D_{{{\text{nAChd}},p}}^{\lambda } \left( {\left( {0,0} \right),\left( {0,0} \right)} \right) & = \left[ {\left\{ {\frac{{D_{{{\text{nAChd}}}}^{\lambda } \left( {0,0} \right)}}{20}} \right\}^{p} + \left\{ {\frac{{D_{{{\text{nAChd}}}}^{\lambda } \left( {0,0} \right)}}{20}} \right\}^{p} } \right]^{{{\raise0.7ex\hbox{$1$} \!\mathord{\left/ {\vphantom {1 p}}\right.\kern-0pt} \!\lower0.7ex\hbox{$p$}}}} \\ & = \left[ {\left\{ {\frac{1}{20} \times \frac{{\left| {0 - 0} \right|}}{{\max \left\{ {\lambda ,0} \right\}\max \left\{ {\lambda ,0} \right\}}}} \right\}^{p} + \left\{ {\frac{1}{20} \times \frac{{\left| {0 - 0} \right|}}{{\max \left\{ {\lambda ,0} \right\}\max \left\{ {\lambda ,0} \right\}}}} \right\}^{p} } \right]^{{{\raise0.7ex\hbox{$1$} \!\mathord{\left/ {\vphantom {1 p}}\right.\kern-0pt} \!\lower0.7ex\hbox{$p$}}}} \\ & = \left( {0 + 0} \right)^{{{\raise0.5ex\hbox{$\scriptstyle 1$} \kern-0.1em/\kern-0.15em \lower0.25ex\hbox{$\scriptstyle p$}}}} = 0 \\ \end{aligned}$$

Thus, the result holds.

#### Proposition 4

For any two PFSs, $P = \left( {a,b} \right)$ and $Q = \left( {c,d} \right)$ in general, we can establish the relation, $D_{{{\text{Chd}},2}} \left( {P,Q} \right) \le D_{{{\text{Chd}},1}} \left( {P,Q} \right)$ and $D_{{{\text{nAChd}},2}}^{1} \left( {P,Q} \right) \le D_{{{\text{nAChd}},1}}^{1} \left( {P,Q} \right)$, provided $\lambda = 1$.

#### Proof

For $P = \left( {a,b} \right)$ and $Q = \left( {c,d} \right)$, by definition of our constructed distances we have,

49 $$\begin{aligned} D_{{{\text{Chd}},1}} \left( {P,Q} \right) & = \frac{1}{\sqrt 2 } \times \left[ {D_{{{\text{Chd}}}} \left( {a,c} \right) + D_{{{\text{Chd}}}} \left( {b,d} \right)} \right] \\ & = \frac{1}{\sqrt 2 } \times \left[ {\left( {\frac{{\left\| {a - c} \right\|}}{{\sqrt {1 + \left\| a \right\|^{2} } \sqrt {1 + \left\| c \right\|^{2} } }}} \right) + \left( {\frac{{\left\| {b - d} \right\|}}{{\sqrt {1 + \left\| b \right\|^{2} } \sqrt {1 + \left\| d \right\|^{2} } }}} \right)} \right] \\ \end{aligned}$$

50 $$\begin{aligned} D_{{{\text{Chd}},2}} \left( {P,Q} \right) & = \left( {\frac{1}{{2^{{\left( {1 - \tfrac{2}{2}} \right)}} }} \times \left[ {\left\{ {D_{{{\text{Chd}}}} \left( {a,c} \right)} \right\}^{2} + \left\{ {D_{{{\text{Chd}}}} \left( {b,d} \right)} \right\}^{2} } \right]} \right)^{\frac{1}{2}} \\ & = \left( {\frac{1}{1} \times \left[ {\left\{ {\frac{{\left\| {a - c} \right\|}}{{\sqrt {1 + \left\| a \right\|^{2} } \sqrt {1 + \left\| c \right\|^{2} } }}} \right\}^{2} + \left\{ {\frac{{\left\| {b - d} \right\|}}{{\sqrt {1 + \left\| b \right\|^{2} } \sqrt {1 + \left\| d \right\|^{2} } }}} \right\}^{2} } \right]} \right)^{\frac{1}{2}} \\ & = \left( {\left[ {\frac{{\left\| {a - c} \right\|^{2} }}{{\left( {1 + \left\| a \right\|^{2} } \right)\left( {1 + \left\| c \right\|^{2} } \right)}} + \frac{{\left\| {b - d} \right\|^{2} }}{{\left( {1 + \left\| b \right\|^{2} } \right)\left( {1 + \left\| d \right\|^{2} } \right)}}} \right]} \right)^{\frac{1}{2}} \\ \end{aligned}$$

Since, $0 \le a,b,c,d \le 1$, so from (49) and (50) we have $\left\| {a - c} \right\|^{2} \le \left\| {a - c} \right\|$ and

$$\begin{aligned} \sqrt {1 + \left\| a \right\|^{2} } \sqrt {1 + \left\| c \right\|^{2} } & \le \left( {\sqrt {1 + \left\| a \right\|^{2} } \sqrt {1 + \left\| c \right\|^{2} } } \right)^{2} = \left( {1 + \left\| a \right\|^{2} } \right)\left( {1 + \left\| c \right\|^{2} } \right) \\ & \Rightarrow \frac{1}{{\left( {1 + \left\| a \right\|^{2} } \right)\left( {1 + \left\| c \right\|^{2} } \right)}} \le \frac{1}{{\sqrt {1 + \left\| a \right\|^{2} } \sqrt {1 + \left\| c \right\|^{2} } }} \\ \end{aligned}$$

Combining both results, we have,

$$\frac{{\left\| {a - c} \right\|^{2} }}{{\left( {1 + \left\| a \right\|^{2} } \right)\left( {1 + \left\| c \right\|^{2} } \right)}} \le \frac{{\left\| {a - c} \right\|}}{{\sqrt {1 + \left\| a \right\|^{2} } \sqrt {1 + \left\| c \right\|^{2} } }}$$, and $$\frac{{\left\| {b - d} \right\|^{2} }}{{\left( {1 + \left\| b \right\|^{2} } \right)\left( {1 + \left\| d \right\|^{2} } \right)}} \le \frac{{\left\| {b - d} \right\|}}{{\sqrt {1 + \left\| b \right\|^{2} } \sqrt {1 + \left\| d \right\|^{2} } }}$$.

Therefore, we can say that $D_{{{\text{Chd}},2}} \left( {P,Q} \right) \le D_{{{\text{Chd}},1}} \left( {P,Q} \right)$.

Similarly, we have

$$\begin{aligned} D_{nAChd,1}^{1} \left( {P,Q} \right) & = \left[ {\frac{{D_{nAChd}^{1} \left( {a,c} \right)}}{20} + \frac{{D_{nAChd}^{1} \left( {b,d} \right)}}{20}} \right] \\ & = \left[ {\left( {\frac{1}{20} \times \frac{{\left| {a - c} \right|}}{{\max \left\{ {1,\left| a \right|} \right\}\max \left\{ {1,\left| c \right|} \right\}}}} \right) + \left( {\frac{1}{20} \times \frac{{\left| {b - d} \right|}}{{\max \left\{ {1,\left| b \right|} \right\}\max \left\{ {1,\left| d \right|} \right\}}}} \right)} \right] \\ & = \frac{1}{20} \times \left[ {\left| {a - c} \right| + \left| {b - d} \right|} \right] \\ \end{aligned}$$

$$\begin{aligned} D_{{{\text{nAChd}},2}}^{1} \left( {P,Q} \right) & = \left( {\left[ {\left\{ {\frac{{D_{{{\text{nAChd}}}}^{1} \left( {a,c} \right)}}{20}} \right\}^{2} + \left\{ {\frac{{D_{{{\text{nAChd}}}}^{1} \left( {b,d} \right)}}{20}} \right\}^{2} } \right]} \right)^{\frac{1}{2}} \\ & = \left( {\frac{1}{400} \times \left[ {\left\{ {\frac{{\left| {a - c} \right|}}{{\max \left\{ {1,\left| a \right|} \right\}\max \left\{ {1,\left| c \right|} \right\}}}} \right\}^{2} + \left\{ {\frac{{\left| {b - d} \right|}}{{\max \left\{ {1,\left| b \right|} \right\}\max \left\{ {1,\left| d \right|} \right\}}}} \right\}^{2} } \right]} \right)^{\frac{1}{2}} \\ & = \left( {\frac{1}{400} \times \left[ {\left| {a - c} \right|^{2} + \left| {b - d} \right|^{2} } \right]} \right)^{\frac{1}{2}} \\ \end{aligned}$$

Now, for the values of $a,b,c,d$ within the interval $[0,1]$, we have $\left| {a - c} \right|^{2} \le \left| {a - c} \right|$ and

$$\begin{aligned} & \quad \frac{1}{20}\left| {a - c} \right|^{2} \le \frac{1}{20}\left| {a - c} \right| \\ & \Rightarrow \frac{1}{400}\left| {a - c} \right|^{2} \le \frac{1}{20}\left| {a - c} \right|^{2} \le \frac{1}{20}\left| {a - c} \right| \\ \end{aligned}$$

and other component-wise similar results.

This shall necessarily imply $D_{{{\text{nAChd}},2}}^{1} \left( {P,Q} \right) \le D_{{{\text{nAChd}},1}}^{1} \left( {P,Q} \right)$. This completes the proof.

## Drawbacks of the existing measures and comparative analysis

Here in this section, we showcase some drawbacks of the existing distance measures and hence establish the efficiency and superiority of our newly proposed measures. To fulfil our objective, we consider 12 (twelve) different profiles of IFSs, which are presented in Table 1. The corresponding evaluated distance measure values under different methods are shown in Table 2.

**Table 1:** Profiles of IFSs

Profiles	Pairs of IFSs
Profile 1	$A = \left( {0.5,0.5} \right),\;B = \left( {0.4,0.55} \right)$
Profile 2	$A = \left( {0.3,0.6} \right),\;B = \left( {0.4,0.55} \right)$
Profile 3	$A = \left( {0.2,0.4} \right),\;B = \left( {0.4,0.45} \right)$
Profile 4	$A = \left( {0.6,0.4} \right),\;B = \left( {0.4,0.45} \right)$
Profile 5	$A = \left( {0.4,0.4} \right),\;B = \left( {0.5,0.5} \right)$
Profile 6	$A = \left( {0.6,0.4} \right),\;B = \left( {0.5,0.5} \right)$
Profile 7	$A = \left( {0.3,0.3} \right),\;B = \left( {0.2,0.2} \right)$
Profile 8	$A = \left( {0.4,0.5} \right),\;B = \left( {0.5,0.4} \right)$
Profile 9	$A = \left( {0.0,0.0} \right),\;B = \left( {1.0,0.0} \right)$
Profile 10	$A = \left( {0.0,0.0} \right),\;B = \left( {0.5,0.5} \right)$
Profile 11	$A = \left( {0.1,0.3} \right),\;B = \left( {0.2,0.4} \right)$
Profile 12	$A = \left( {0.2,0.5} \right),\;B = \left( {0.3,0.7} \right)$

**Table 2:**
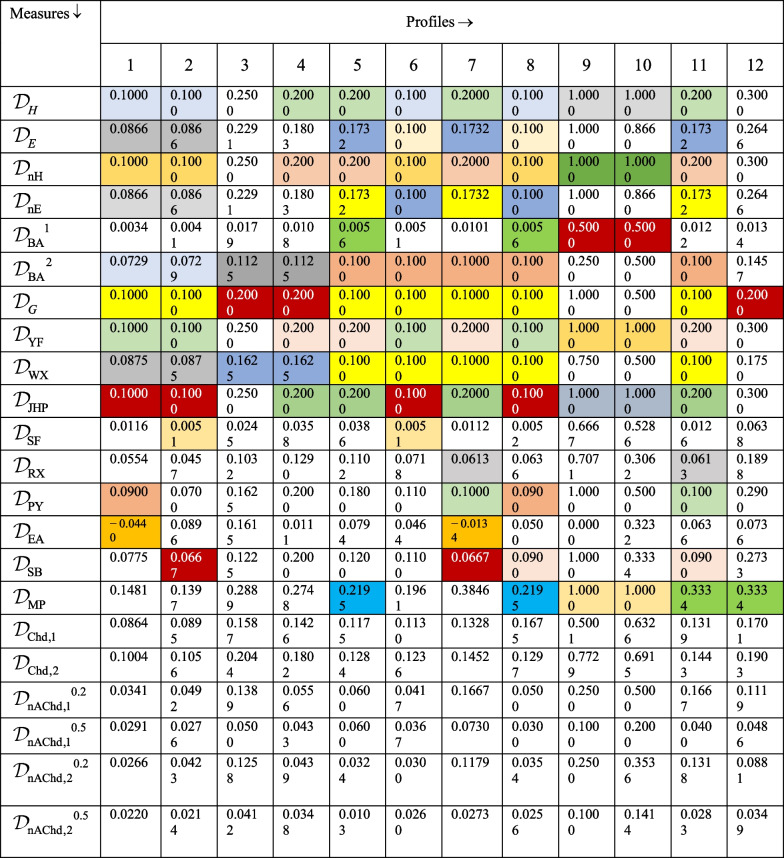
Comparison of the computed distance measure values

*Here in each row, identical color shades are used to depict the same values of distance obtained by the distance measures, for different pairs of profiles.

From the results obtained in Table 2, the inadequacies in case of each of the existing measures are listed down under:

(i) *Hamming distance measure *($D_{H}$) Even though each of the 12 profiles considered is non-identical, yet the measure $D_{H}$ obtains identical distance measure value of- 0.1000 for profiles 1, 2, 6, 8; 0.2000 for profiles 4, 5, 7, 11; 1.0000 for profiles 9, 10.
(ii) *Euclidean distance measure* ($D_{E}$) $D_{E}$ obtains identical distance values as, 0.0866 for profiles 1, 2; 0.1732 for profiles 5, 7, 11; 0.1000 for profiles 6, 8.
(iii) *Normalized Hamming distance measure* ($D_{{{\text{nH}}}}$) $D_{{{\text{nH}}}}$ obtains distance value of 0.1000 for profiles 1, 2, 6, 8; 0.2000 for 4, 5, 7, 11; 1.0000 for profiles 9, 10.
(iv) *Normalized Euclidean distance measure* ($D_{{{\text{nE}}}}$) $D_{{{\text{nE}}}}$ fails to obtain dissimilar distance values for different profiles and ends up obtaining- 0.0866 for profiles 1, 2; 0.1732 for 5, 7, 11; 0.1000 for profiles 6, 8.
(v) *Baccour & Alimi’s First Squared distance measure* ($D_{{{\text{BA}}}}^{1}$) $D_{{{\text{BA}}}}^{1}$ yet again obtains identical distance values for non-similar profiles 5, 8; and 9, 10.
(vi) *Baccour & Alimi’s second squared distance measure* ($D_{{{\text{BA}}}}^{2}$) $D_{{{\text{BA}}}}^{2}$ yields distance values, 0.0729 for profiles 1, 2; 0.1125 for profiles 3, 4; 0.1000 for profiles 5, 6, 7, 8, 11; for non-similar profiles of fuzzy numbers.
(vii) *Grzegorzewski’s distance measure* ($D_{G}$) $D_{G}$ attains same distance measure value of 0.1000 for profiles 1, 2, 5, 6, 7, 8, 11 and 0.2000 for profiles 4, 5, 12, for each of the dissimilar profiles.
(viii) *Yang & Chiclana’s distance measure* ($D_{{{\text{YF}}}}$) $D_{{{\text{YF}}}}$ fails to obtain unique values of similarity for each of the non-similar profiles and ends up obtaining values as, 0.1000 for profiles 1, 2, 6, 8; 0.2000 for 4, 5, 7; 1.0000 for profiles 9, 10.
(ix) *Wang & Xin’s distance measure* ($D_{{{\text{WX}}}}$) Like most others as discussed, $D_{{{\text{WX}}}}$ attains identical distance measure values of 0.0875 for profiles 1, 2; 0.1625 for profiles 3, 4; 0.1000 for profiles 5, 6, 7, 8, 11.
(x) *Jin *et al*.’s distance measure* ($D_{{{\text{JHP}}}}$) $D_{{{\text{JHP}}}}$ obtains 0.1000 for 1st, 2nd, 6th, 8th profiles; 0.2000 for 4th, 5th, 7th, 11th profiles; 1.0000 for 9th, 10th profiles, which is counter-intuitive as the profiles considered are non-identical.
(xi) *Song *et al*.’s distance measure* ($D_{{{\text{SF}}}}$) For the profiles 2nd and 6th, $D_{{{\text{SF}}}}$ obtains identical distance values, which is quite illogical since the profiles are chosen differently.
(xii) *Ren *et al*.’s distance measure* ($D_{{{\text{RX}}}}$) The distance measure $D_{{{\text{RX}}}}$ obtains identical values of similarity for non-similar pairs of PFSs in profiles 7 and 11.
(xiii) *Peng *et al*.’s distance measure* ($D_{{{\text{PY}}}}$) Likewise, for pairs of profiles (1,8) and (7,11), the similarity value results obtained with measure $D_{{{\text{PY}}}}$ are identical, which is counter-intuitive.
(xiv) *Ejegwa & Awolola distance measure* ($D_{{{\text{EA}}}}$) However, non-acceptable distance measure results are obtained with $D_{{{\text{EA}}}}$ distance measure for profiles 1 and 7. Clearly evident from Table 2 that, $D_{{{\text{EA}}}}$ determines negative values of distance for these profiles which is absurd.
(xv) *Sarkar & Biswas distance measure* ($D_{{{\text{SB}}}}$) The inefficacy of $D_{{{\text{SB}}}}$ distance measure is also showcased for pairs of profiles (2,7) and (8,11), owing to its evaluation of identical distance value for different pairs of PFSs in them.
(xvi) *Mahanta & Panda distance measure* ($D_{{{\text{MP}}}}$) The distance measure by Mahanta & Panda, $D_{{{\text{MP}}}}$ could not distinguish between pairs of profiles (5,8), (9,10) and (11,12), which indicates a flaw in the structural formulation of their measure.
(xvii) *Proposed measures* ($D_{{{\text{Chd}},p}}$ *and* $D_{{{\text{nAChd}},p}}^{\lambda }$) On careful observation of Table 2, we find that for our proposed measures whether be it $D_{{{\text{Chd}},p}}$ or $D_{{{\text{nAChd}},p}}^{\lambda }$, neither of them attains identical values of distances. This is logical and at par with human intuition that, since we have considered different profiles of fuzzy numbers, so their respective evaluated distance measure values should be non-identical as well. Even though, most of the existing distance measures failed to obtain logical outcomes, but our proposed measures proved to be successful and effective. Hence, our proposed measures are more efficient, more common-sensical, feasible and are worthy of due consideration.

## Practical applications

In this section, we demonstrate the applicability and effectiveness of our proposed measures by illustrating the procedure for multicriteria decision-making, when problems from the field of pattern recognition and medical decision-making are considered.

### Pattern recognition

#### Description

Pattern recognition refers to the process of recognition or identification of patterns and regularities in a data sample. The arena of pattern recognition owes its origins in engineering and statistics, and it has become one of the widely researched areas with applications in machine learning, image analysis, signal processing, etc. In decision-making problems, the idea of pattern recognition usually consists of a certain set of known patterns and an unknown pattern, which we need to identify or classify into the known patterns. Briefly speaking, the known pattern having the maximum similarity or least dissimilarity may be identified as the most deserving pattern. Hereby, we illustrate a numerical scenario below, to give a vivid visualization of the process.

#### Assumptions

Suppose we consider three known patterns $P_{i} \,\left( {i = 1,2,3} \right)$, where the preference information for the patterns is represented by PFSs in a universe of discourse $U = \left\{ {x_{1} ,x_{2} ,x_{3} } \right\}$ as given below.

$$\begin{aligned} P_{1} & = \left\{ {\left( {x_{1} ,1.0,0.0} \right),\;\left( {x_{2} ,0.6,0.2} \right),\;\left( {x_{3} ,0.4,0.3} \right)} \right\} \\ P_{2} & = \left\{ {\left( {x_{1} ,0.7,0.1} \right),\;\left( {x_{2} ,1.0,0.0} \right),\;\left( {x_{3} ,0.2,0.6} \right)} \right\} \\ P_{3} & = \left\{ {\left( {x_{1} ,0.8,0.1} \right),\;\left( {x_{2} ,0.5,0.1} \right),\;\left( {x_{3} ,0.9,0.0} \right)} \right\} \\ \end{aligned}$$

Now, we assume that we have an unknown pattern $Q$, which needs to be recognized or classified. Let $Q \in PFSs\left( U \right)$ and $Q = \left\{ {\left( {x_{1} ,0.6,0.2} \right),\;\left( {x_{2} ,0.9,0.1} \right),\;\left( {x_{3} ,0.1,0.7} \right)} \right\}$.

#### Objective

Our aim is to classify or characterize the unknown pattern $Q$ into one of the known patterns $P_{1}$, $P_{2}$ or $P_{3}$. Based on the least difference or maximum similarity, we can obtain our required suitable pattern.

#### Results

After having evaluated the distance values between the set of patterns $\left( {P_{1} ,Q} \right)$, $\left( {P_{2} ,Q} \right)$ and $\left( {P_{3} ,Q} \right)$ we have obtained the following results.

By generalized chordal distance,

$$\begin{gathered} D_{{{\text{Chd}},1}} \left( {P_{1} ,Q} \right) = 0.5101;\;D_{{{\text{Chd}},1}} \left( {P_{2} ,Q} \right) = 0.2469;\;D_{{{\text{Chd}},1}} \left( {P_{3} ,Q} \right) = 0.5172 \hfill \\ D_{{{\text{Chd}},2}} \left( {P_{1} ,Q} \right) = 0.8527;\;D_{{{\text{Chd}},2}} \left( {P_{2} ,Q} \right) = 0.5334;\;D_{{{\text{Chd}},2}} \left( {P_{3} ,Q} \right) = 0.8649 \hfill \\ \end{gathered}$$

By non-Archimedean chordal distance,

$$\begin{aligned} D_{{{\text{nAChd}},1}}^{0.2} \left( {P_{1} ,Q} \right) = & 0.4343;\;D_{{{\text{nAChd}},1}}^{0.2} \left( {P_{2} ,Q} \right) = 0.2302;\;D_{{{\text{nAChd}},1}}^{0.2} \left( {P_{3} ,Q} \right) = 0.5023 \\ D_{{{\text{nAChd}},1}}^{0.6} \left( {P_{1} ,Q} \right) = & 0.1624;\;D_{{{\text{nAChd}},1}}^{0.6} \left( {P_{2} ,Q} \right) = 0.0667;\;D_{{{\text{nAChd}},1}}^{0.6} \left( {P_{3} ,Q} \right) = 0.1703 \\ D_{{{\text{nAChd}},2}}^{0.2} \left( {P_{1} ,Q} \right) = & 0.5001;\;D_{{{\text{nAChd}},2}}^{0.2} \left( {P_{2} ,Q} \right) = 0.3426;\;D_{{{\text{nAChd}},2}}^{0.2} \left( {P_{3} ,Q} \right) = 0.5324 \\ D_{{{\text{nAChd}},2}}^{0.6} \left( {P_{1} ,Q} \right) = & 0.1628;\;D_{{{\text{nAChd}},2}}^{0.6} \left( {P_{2} ,Q} \right) = 0.1042;\;D_{{{\text{nAChd}},2}}^{0.6} \left( {P_{3} ,Q} \right) = 0.1718. \\ \end{aligned}$$

#### Discussion

Therefore, based on the distance measure values obtained, the pattern $P_{2}$ is the most suitable pattern, which can be characterized with the pattern $Q$ (unknown). Furthermore, a comparison table showing the suitable pattern under various other methods are also presented in Table 3.

**Table 3:** Results obtained under various methods for the most suitable pattern

Distance measures	Distances between patterns			Most suitable pattern
	$\left( {P_{1} ,Q} \right)$	$\left( {P_{2} ,Q} \right)$	$\left( {P_{3} ,Q} \right)$	
${\mathbf{\mathcal{D}}}_{H}$	1.1000	0.3000	1.4000	$P_{2}$
${\mathbf{\mathcal{D}}}_{E}$	0.5657	0.1732	0.8718	$P_{2}$
${\mathbf{\mathcal{D}}}_{nH}$	0.3667	0.1000	0.4667	$P_{2}$
${\mathbf{\mathcal{D}}}_{{{\varvec{nE}}}}$	0.3266	0.1000	0.5033	$P_{2}$
${\mathbf{\mathcal{D}}}_{{{\text{BA}}}}^{1}$	0.0803	0.0241	0.1983	$P_{2}$
${\mathbf{\mathcal{D}}}_{{{\text{BA}}}}^{2}$	0.2754	0.1000	0.3316	$P_{2}$
${\mathbf{\mathcal{D}}}_{G}$	0.3667	0.1000	0.4667	$P_{2}$
${\mathbf{\mathcal{D}}}_{{{\text{YF}}}}$	0.3667	0.1000	0.4667	$P_{2}$
${\mathbf{\mathcal{D}}}_{{{\text{WX}}}}$	0.3250	0.1000	0.4167	$P_{2}$
${\mathbf{\mathcal{D}}}_{{{\text{JHP}}}}$	0.3667	0.1000	0.4667	$P_{2}$
${\mathbf{\mathcal{D}}}_{{{\text{SF}}}}$	0.1112	0.0229	0.2005	$P_{2}$
${\mathbf{\mathcal{D}}}_{{{\text{RX}}}}$	0.4823	0.1439	0.5393	$P_{2}$
${\mathbf{\mathcal{D}}}_{{{\text{PY}}}}$	0.4967	0.1500	0.5467	$P_{2}$
${\mathbf{\mathcal{D}}}_{{{\text{EA}}}}$	0.0903	0.0687	0.1146	$P_{2}$
${\mathbf{\mathcal{D}}}_{{{\text{SB}}}}$	0.4889	0.1856	0.4933	$P_{2}$
${\mathbf{\mathcal{D}}}_{{{\text{MP}}}}$	0.5375	0.1551	0.5995	$P_{2}$
${\mathbf{\mathcal{D}}}_{{{\text{Chd}},1}}$	0.5101	0.2469	0.5172	$P_{2}$
${\mathbf{\mathcal{D}}}_{{{\text{Chd}},2}}$	0.8527	0.5334	0.8649	$P_{2}$
${\mathbf{\mathcal{D}}}_{{{\text{nAChd}},1}}^{0.2}$	0.4343	0.2302	0.5023	$P_{2}$
${\mathbf{\mathcal{D}}}_{{{\text{nAChd}},1}}^{0.6}$	0.1624	0.0667	0.1703	$P_{2}$
${\mathbf{\mathcal{D}}}_{{{\text{nAChd}},2}}^{0.2}$	0.5001	0.3426	0.5324	$P_{2}$
${\mathbf{\mathcal{D}}}_{{{\text{nAChd}},2}}^{0.6}$	0.1628	0.1042	0.1718	$P_{2}$

**Fig. 11:**
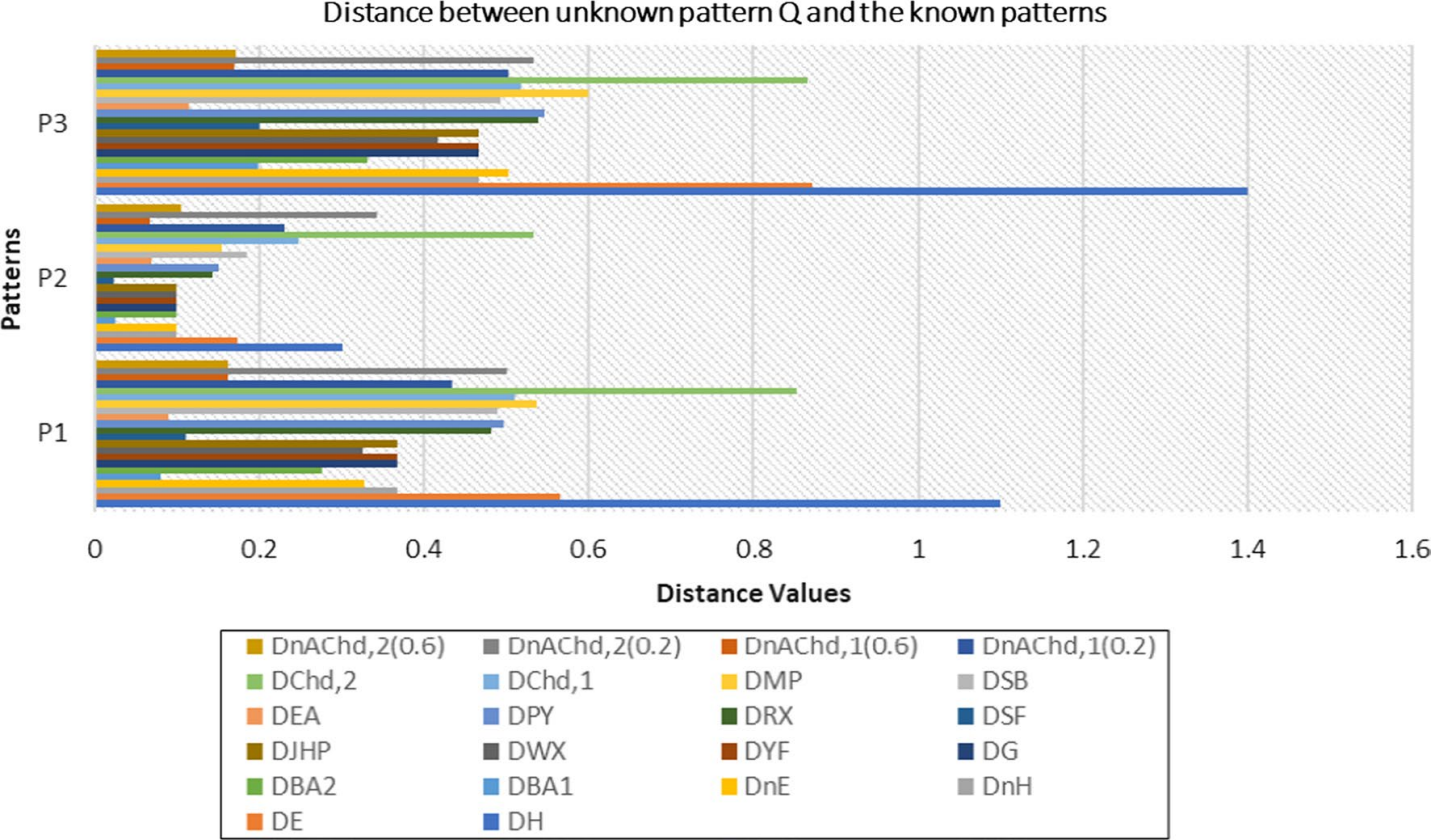
Distance measure values between the unknown and the known patterns

Moreover, a graphical representation of distance measure values and the most suitable pattern being $P_{2}$ is evident from Fig. 11, as shown below.

### Medical diagnosis

#### Description

Medical diagnosis is referred to as the process of determining the disease or medical condition, which is causing the patient’s illness or sickness. The process of diagnosis is achieved via physical examination of the patient needing medical attention and also, acquiring knowledge about the background history of diseases. Very seldom does it happen that the diagnosis process is very effortless or simple, but in most situations it is very much challenging as the information we collect or refer to, is too much unclear or vague. In this context, the application of fuzzy sets and their ability to represent uncertain information helps us attain convincing results, and hence, we impose PFSs with the motive to effectively conduct the medical decision-making process. Precisely, the medical decision-making process comprises a set of diagnoses and a set of symptoms. When we represent the information about a patient with respect to the set of all possible symptoms by an ordered set, then based on the maximum similarity or least dissimilarity between the patient and the set of diagnoses, we determine the disease causing the patient’s medical condition. Hereby, a numerical illustration is presented below for enhanced understanding.

#### Assumptions

Let us consider a set which represents the set of diagnoses,

$P = \left\{ {P_{1} \left( {{\text{Malaria}}} \right),P_{2} \left( {{\text{Kidney}}\;{\text{Stone}}} \right),P_{3} \left( {{\text{Typhoid}}} \right),P_{4} \left( {{\text{Dengue}}} \right),P_{5} \left( {{\text{Viral}}\;{\text{Fever}}} \right)} \right\}$, and equivalently we consider a set of symptoms,

$$S = \left\{ {s_{1} \left( {{\text{Headache}}} \right),s_{2} \left( {{\text{Cough}}} \right),s_{3} \left( {{\text{Chest}}\;{\text{Pain}}} \right),s_{4} \left( {{\text{Temperature}}} \right),s_{5} \left( {{\text{Stomach}}\;{\text{Pain}}} \right)} \right\}.$$

We have a patient $Q$, which we represent by a PFS so that the patient with respect to the given set of symptoms has the following form,

$$Q\left( {{\text{Patient}}} \right) = \left\{ {\left( {s_{1} ,0.8,0.2} \right),\left( {s_{2} ,0.4,0.4} \right),\left( {s_{3} ,0.1,0.8} \right),\left( {s_{4} ,0.5,0.4} \right),\left( {s_{5} ,0.2,0.6} \right)} \right\}.$$

Moreover, the set of diagnoses $P_{i} \,\left( {i = 1,2,3,4,5} \right)$ can also have the following representations in the form of PFSs, with respect to all possible symptoms.

$$\begin{aligned} P_{1} \left( {{\text{Malaria}}} \right) & = \left\{ {\left( {s_{1} ,0.7,0.1} \right),\left( {s_{2} ,0.5,0.5} \right),\left( {s_{3} ,0.2,0.8} \right),\left( {s_{4} ,0.5,0.3} \right),\left( {s_{5} ,0.3,0.7} \right)} \right\} \\ P_{2} \left( {{\text{Kidney}}\,{\text{Stone}}} \right) & = \left\{ {\left( {s_{1} ,0.1,0.4} \right),\left( {s_{2} ,0.9,0.0} \right),\left( {s_{3} ,0.5,0.2} \right),\left( {s_{4} ,0.7,0.1} \right),\left( {s_{5} ,0.8,0.1} \right)} \right\} \\ P_{3} \left( {{\text{Typhoid}}} \right) & = \left\{ {\left( {s_{1} ,0.2,0.7} \right),\left( {s_{2} ,1.0,0.0} \right),\left( {s_{3} ,0.6,0.3} \right),\left( {s_{4} ,0.1,0.9} \right),\left( {s_{5} ,0.3,0.4} \right)} \right\} \\ P_{4} \left( {{\text{Dengue}}} \right) & = \left\{ {\left( {s_{1} ,0.6,0.1} \right),\left( {s_{2} ,0.7,0.2} \right),\left( {s_{3} ,0.5,0.4} \right),\left( {s_{4} ,0.2,0.3} \right),\left( {s_{5} ,0.7,0.3} \right)} \right\} \\ P_{5} \left( {{\text{Viral}}\,{\text{Fever}}} \right) & = \left\{ {\left( {s_{1} ,0.9,0.0} \right),\left( {s_{2} ,0.1,0.2} \right),\left( {s_{3} ,0.4,0.4} \right),\left( {s_{4} ,0.1,0.2} \right),\left( {s_{5} ,0.8,0.2} \right)} \right\} \\ \end{aligned}$$

#### Objective

Our target is to classify the patient $Q$, into one of the classes (diagnoses) $P_{1}$, $P_{2}$, $P_{3}$, $P_{4}$ or $P_{5}$. Depending on the least value of distance obtained between the patient $Q$ and any of $P_{1}$, $P_{2}$, $P_{3}$, $P_{4}$ or $P_{5}$, we can identify the exact disease from which the patient is suffering.

#### Results

The evaluated distance values with respect to both our proposed measures are given below.

By generalized chordal distance,

$$\begin{aligned} D_{{{\text{Chd}},1}} \left( {P_{1} ,Q} \right) = 0.0912;\;D_{Chd,1} \left( {P_{2} ,Q} \right) = 0.5321;\;D_{Chd,1} \left( {P_{3} ,Q} \right) = 0.6036;\;D_{{{\text{Chd}},1}} \left( {P_{4} ,Q} \right) & = 0.3418; \\ D_{{{\text{Chd}},1}} \left( {P_{5} ,Q} \right) & = 0.3477 \\ \end{aligned}$$

$$\begin{aligned} D_{{{\text{Chd}},2}} \left( {P_{1} ,Q} \right) = 0.2924;\;D_{{{\text{Chd}},2}} \left( {P_{2} ,Q} \right) = 0.7636;\;D_{{{\text{Chd}},2}} \left( {P_{3} ,Q} \right) = 0.8522;\;D_{{{\text{Chd}},2}} \left( {P_{4} ,Q} \right) & = 0.5426; \\ D_{{{\text{Chd}},2}} \left( {P_{5} ,Q} \right) & = 0.6009 \\ \end{aligned}$$

By non-Archimedean chordal distance,

$$\begin{aligned} D_{{{\text{nAChd}},1}}^{0.5} \left( {P_{1} ,Q} \right) = 0.0323;\;D_{{{\text{nAChd}},1}}^{0.5} \left( {P_{2} ,Q} \right) = 0.2221;\;D_{{{\text{nAChd,}}1}}^{0.5} \left( {P_{3} ,Q} \right) = 0.2518;\;D_{{{\text{nAChd}},1}}^{0.5} \left( {P_{4} ,Q} \right) & = 0.1430; \\ D_{{{\text{nAChd}},1}}^{0.5} \left( {P_{5} ,Q} \right) & = 0.1564 \\ \end{aligned}$$

$$\begin{aligned} D_{{{\text{nAChd}},1}}^{0.7} \left( {P_{1} ,Q} \right) = 0.0201;\;D_{{{\text{nAChd}},1}}^{0.7} \left( {P_{2} ,Q} \right) = 0.1263;\;D_{{{\text{nAChd}},1}}^{0.7} \left( {P_{3} ,Q} \right) = 0.1456;\;D_{{{\text{nAChd}},1}}^{0.7} \left( {P_{4} ,Q} \right) & = 0.0843; \\ D_{{{\text{nAChd}},1}}^{0.7} \left( {P_{5} ,Q} \right) & = 0.0854 \\ \end{aligned}$$

$$\begin{aligned} D_{{{\text{nAChd,}}2}}^{0.5} \left( {P_{1} ,Q} \right) = 0.0506;D_{{{\text{nAChd}},2}}^{0.5} \left( {P_{2} ,Q} \right) = 0.2127;\;D_{{{\text{nAChd}},2}}^{0.5} \left( {P_{3} ,Q} \right) = 0.2436;\;D_{{{\text{nAChd}},2}}^{0.5} \left( {P_{4} ,Q} \right) & = 0.1551; \\ D_{{{\text{nAChd}},2}}^{0.5} \left( {P_{5} ,Q} \right) & = 0.1668 \\ \end{aligned}$$

$$\begin{aligned} D_{{{\text{nAChd}},2}}^{0.7} \left( {P_{1} ,Q} \right) = 0.0260\,;\,D_{{{\text{nAChd}},2}}^{0.7} \left( {P_{2} ,Q} \right) = 0.1235\,;\,D_{{{\text{nAChd}},2}}^{0.7} \left( {P_{3} ,Q} \right) = 0.1462;\;D_{{{\text{nAChd}},2}}^{0.7} \left( {P_{4} ,Q} \right) & = 0.0721; \\ D_{{{\text{nAChd}},2}}^{0.7} \left( {P_{5} ,Q} \right) & = 0.0824 \\ \end{aligned}$$

#### Discussion

From the distance values obtained, the least distance is found for the pair $\left( {P_{1} ,Q} \right)$. Hence, the patient $Q$ is more likely to suffer from illness caused by $P_{1}$ (Malaria).

Further, some of the comparison results under different distance measures are depicted in Table 4.

**Table 4:** Determination of the patient’s suffering illness under various methods

Distance measures	Distance values between					Patient $Q$ suffering from disease
	$\left( {P_{1} ,Q} \right)$	$\left( {P_{2} ,Q} \right)$	$\left( {P_{3} ,Q} \right)$	$\left( {P_{4} ,Q} \right)$	$\left( {P_{5} ,Q} \right)$	
${\mathbf{\mathcal{D}}}_{H}$	0.8000	2.7000	2.4000	1.9000	2.3000	$P_{1}$ (Malaria)
${\mathbf{\mathcal{D}}}_{E}$	0.3317	1.1225	1.0392	0.7874	0.9539	$P_{1}$ (Malaria)
${\mathbf{\mathcal{D}}}_{{{\text{nH}}}}$	0.1600	0.5400	0.4800	0.3800	0.4600	$P_{1}$ (Malaria)
${\mathbf{\mathcal{D}}}_{{{\text{nE}}}}$	0.1483	0.5020	0.4648	0.3521	0.4266	$P_{1}$ (Malaria)
${\mathbf{\mathcal{D}}}_{{{\text{BA}}}}^{1}$	0.0070	0.1748	0.1500	0.0607	0.1000	$P_{1}$ (Malaria)
${\mathbf{\mathcal{D}}}_{{{\text{BA}}}}^{2}$	0.0700	0.4304	0.4276	0.2747	0.3056	$P_{1}$ (Malaria)
${\mathbf{\mathcal{D}}}_{G}$	0.1000	0.5400	0.4800	0.3400	0.3800	$P_{1}$ (Malaria)
${\mathbf{\mathcal{D}}}_{{{\text{YF}}}}$	0.1600	0.5400	0.4800	0.3800	0.4600	$P_{1}$ (Malaria)
${\mathbf{\mathcal{D}}}_{{{\text{WX}}}}$	0.0900	0.4900	0.4550	0.3100	0.3450	$P_{1}$ (Malaria)
${\mathbf{\mathcal{D}}}_{{{\text{JHP}}}}$	0.1600	0.5400	0.4800	0.3800	0.4600	$P_{1}$ (Malaria)
${\mathbf{\mathcal{D}}}_{{{\text{SF}}}}$	0.0299	0.1747	0.1642	0.0784	0.1097	$P_{1}$ (Malaria)
${\mathbf{\mathcal{D}}}_{{{\text{RX}}}}$	0.1295	0.5040	0.5438	0.3352	0.3552	$P_{1}$ (Malaria)
${\mathbf{\mathcal{D}}}_{{{\text{PY}}}}$	0.1280	0.5440	0.5680	0.3700	0.3760	$P_{1}$ (Malaria)
${\mathbf{\mathcal{D}}}_{{{\text{EA}}}}$	0.0476	0.0780	0.0688	0.1316	0.0960	$P_{1}$ (Malaria)
${\mathbf{\mathcal{D}}}_{{{\text{SB}}}}$	0.1440	0.5280	0.5500	0.3460	0.3400	$P_{1}$ (Malaria)
${\mathbf{\mathcal{D}}}_{{{\text{MP}}}}$	0.1343	0.7652	0.7103	0.5514	0.6309	$P_{1}$ (Malaria)
${\mathbf{\mathcal{D}}}_{{{\text{Chd}},1}}$	0.0912	0.5321	0.6036	0.3418	0.3477	$P_{1}$ (Malaria)
${\mathbf{\mathcal{D}}}_{{{\text{Chd}},2}}$	0.2924	0.7636	0.8522	0.5426	0.6009	$P_{1}$ (Malaria)
${\mathbf{\mathcal{D}}}_{{{\text{nAChd,}}1}}^{0.5}$	0.0323	0.2221	0.2518	0.1430	0.1564	$P_{1}$ (Malaria)
${\mathbf{\mathcal{D}}}_{{{\text{nAChd}},1}}^{0.7}$	0.0201	0.1263	0.1456	0.0843	0.0854	$P_{1}$ (Malaria)
${\mathbf{\mathcal{D}}}_{{{\text{nAChd}},2}}^{0.5}$	0.0506	0.2127	0.2436	0.1551	0.1668	$P_{1}$ (Malaria)
${\mathbf{\mathcal{D}}}_{{{\text{nAChd}},2}}^{0.7}$	0.0260	0.1235	0.1462	0.0721	0.0824	$P_{1}$ (Malaria)

**Fig. 12:**
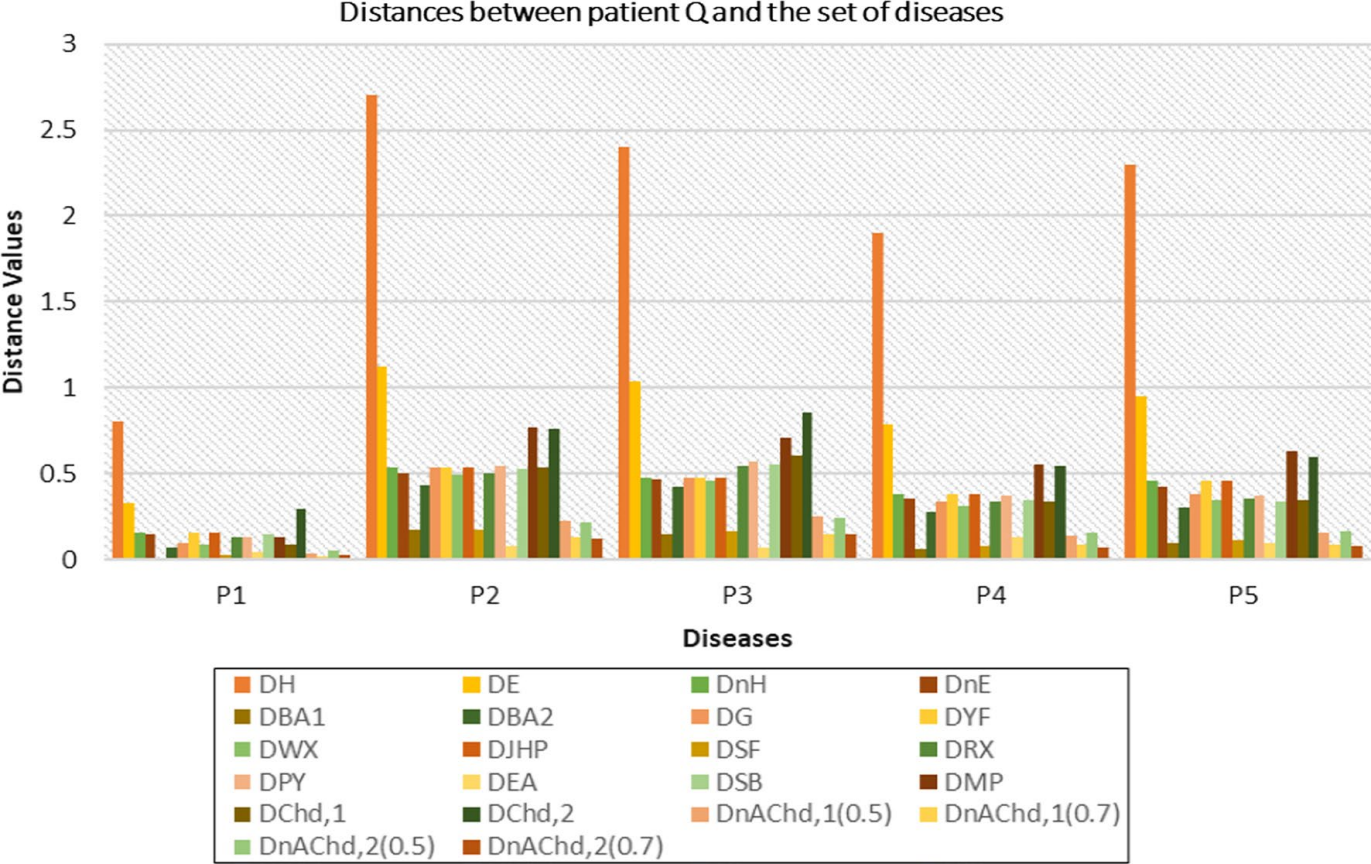
Distance measure values between the patient and the set of diseases

A graphical representation of distance measure values is depicted in Fig. 12, as shown below. From the figure, we can clearly observe that the causal disease of the patient $Q$ is $P_{1}$.

### Effective COVID-19 medicine selection

#### Description

The COVID-19 is a novel strain, and it originates from a family of viruses known as ‘coronaviruses’. COVID-19 virus was first identified within the people of Wuhan city, China, when they seemed to suffer from pneumonia of unknown cause. The virus has spikes all over its surface and that is why it is named after ‘corona’ referring to a crown-like structure [42]. COVID-19 virus is more susceptible to humans and some other animals, and its parent family was also responsible for some highly contagious and fatal diseases in the past, like that of MERS (Middle East Respiratory Syndrome) and SARS (Severe Acute Respiratory Syndrome). The virus has an alarmingly high infectious rate, and it transmits mainly through physical contact with an infected person and also via air transmissions. While considering how infective this virus is and the number of fatalities worldwide due to it, WHO (World Health Organization) declared it a ‘pandemic’ status on March 2020 [43].

COVID-19 active patients often experience symptoms like shortness of breath, fatigue, loss of appetite, loss of taste, loss of smell, cough, fever, nausea, decreased neural response, etc. [44]. Depending upon the symptoms manifested by the COVID-19 active patients, they can be broadly classified into mild, moderate, and critical cases. In some severe cases, reduced ability to move, decreased neural response, and myalgia (or muscle pain) is often observed in patients. The current need of the hour is to provide immediate treatment to these patients so that they can recover as soon as possible. But unfortunately, in absence of strong medical evidence, only a few medical drugs or medicines are authorized for usage to COVID-19 virus-infected patients. Also, some therapeutic techniques are available that can suppress the symptoms shown by the virus and eventually cure the patient.

#### Assumptions

In our present study, we will be considering certain medicinal drugs, which are approved by Food and Drug Administration (FDA), and they can be administered to patients depending upon the past medical history, body’s immune condition, and the nature of viral symptoms exhibited by the patients. Here, we prefer a total of six medicines to treat the COVID-19 patients, namely *Favipiravir* ($M_{1}$), *Hydroxychloroquine* ($M_{2}$), *Remdesivir* ($M_{3}$), *Lopinavir/Ritonavir with Interferon-beta* ($M_{4}$), *Tocilizumab* ($M_{5}$), and *Convalescent Plasma* ($M_{6}$).

*Favipiravir* ($M_{1}$), an antiviral drug developed in Japan, can extract the genetic material and kill the virus responsible for causing influenza in people. It has been tested to show good results in patients showing mild symptoms of the COVID-19 virus. *Hydroxychloroquine* ($M_{2}$), popularly known as an antimalarial drug, can significantly reduce the intensity of COVID-19 symptoms. *Remdesivir* ($M_{3}$) is a clinically tested drug, that can be recommended to mild cases of COVID-19 and patients who are under ventilation. *Lopinavir/Ritonavir with Interferon-beta* ($M_{4}$), although being a drug used primarily for the treatment of HIV(Human Immunodeficiency Virus)/AIDS (Acquired Immune Deficiency Syndrome), are also useful in curing the immune-suppressed cases of COVID-19 infected patients. This drug has the ability to work smoothly without causing further complications to the already existing medical conditions in the patient’s body. Similarly, the drug *Tocilizumab* ($M_{5}$) can be given to patients showing intermediate symptoms of COVID-19, and those who are under constant ventilation. However, severe cases of patients who are constantly under oxygen supply and intaking steroids to suppress the COVID-19 symptoms can be given the therapy of *Convalescent Plasma* ($M_{6}$). This treatment procedure is clinically tested to be effective.

The medicines which are mentioned have their own way of operating and might induce some side effects occasionally. Therefore, certain performance evaluation factors are necessary to assess these medicines. We consider eight such parameters as criteria, namely *Fever* ($C_{1}$), *Shortness of breath* ($C_{2}$), *Cough* ($C_{3}$), *Myalgia* ($C_{4}$), *Anorexia (or loss of appetite)* ($C_{5}$), *Ease breathing* ($C_{6}$), *Antiviral activity* ($C_{7}$), *Coolify* ($C_{8}$).

The assessment matrix depicting the set of medicines with respect to the set of performance evaluation factors is formulated with the help of PFSs and is shown in Table 5.

**Table 5:** The assessment matrix of six medicines with respect to their performance evaluation factors in terms of PFSs

Performance Index $\to$								
Medicines $\downarrow$	$C_{1}$	$C_{2}$	$C_{3}$	$C_{4}$	$C_{5}$	$C_{6}$	$C_{7}$	$C_{8}$
$M_{1}$	$\left( {0.2,0.7} \right)$	$\left( {0.2,0.6} \right)$	$\left( {0.4,0.7} \right)$	$\left( {0.5,0.8} \right)$	$\left( {0.5,0.6} \right)$	$\left( {0.5,0.7} \right)$	$\left( {0.1,0.7} \right)$	$\left( {0.5,0.8} \right)$
$M_{2}$	$\left( {0.4,0.7} \right)$	$\left( {0.3,0.9} \right)$	$\left( {0.1,0.8} \right)$	$\left( {0.4,0.7} \right)$	$\left( {0.7,0.7} \right)$	$\left( {0.4,0.6} \right)$	$\left( {0.4,0.8} \right)$	$\left( {0.3,0.6} \right)$
$M_{3}$	$\left( {0.2,0.9} \right)$	$\left( {0.1,0.8} \right)$	$\left( {0.2,0.7} \right)$	$\left( {0.4,0.4} \right)$	$\left( {0.6,0.7} \right)$	$\left( {0.3,0.8} \right)$	$\left( {0.2,0.9} \right)$	$\left( {0.4,0.7} \right)$
$M_{4}$	$\left( {0.1,0.7} \right)$	$\left( {0.4,0.7} \right)$	$\left( {0.4,0.8} \right)$	$\left( {0.2,0.5} \right)$	$\left( {0.3,0.7} \right)$	$\left( {0.2,0.8} \right)$	$\left( {0.3,0.7} \right)$	$\left( {0.5,0.5} \right)$
$M_{5}$	$\left( {0.1,0.6} \right)$	$\left( {0.2,0.9} \right)$	$\left( {0.3,0.8} \right)$	$\left( {0.4,0.6} \right)$	$\left( {0.1,0.8} \right)$	$\left( {0.9,0.2} \right)$	$\left( {0.8,0.3} \right)$	$\left( {0.8,0.4} \right)$
$M_{6}$	$\left( {0.3,0.8} \right)$	$\left( {0.3,0.6} \right)$	$\left( {0.4,0.9} \right)$	$\left( {0.1,0.8} \right)$	$\left( {0.4,0.7} \right)$	$\left( {0.1,0.9} \right)$	$\left( {0.2,0.8} \right)$	$\left( {0.6,0.7} \right)$

From the set of criteria (or performance evaluation factors) under consideration, we can observe that $C_{1}$, $C_{2}$, $C_{3}$, $C_{4}$, $C_{5}$ are cost-criteria (criteria, which needs to be minimized) and consequently, $C_{6}$, $C_{7}$, $C_{8}$ are benefit-criteria (criteria, which needs to be maximized).

#### Objective

Our objective is to determine the optimum medicine for the treatment of COVID-19 infected patients among $M_{1}$, $M_{2}$, $M_{3}$, $M_{4}$, $M_{5}$, and $M_{6}$. For that purpose, we need to construct an ideal medicine $M^{*}$, so that depending upon the distance measure value between $M^{*}$ and the available set of medicines, the best medicine can be evaluated. Thereby, the smallest distance measure value will correspond to the optimum medicinal choice for the treatment of COVID-19 infected patients.

The formulae for constructing an ideal medicine $M^{*}$ with respect to nature of the criteria is,

$M^{*} = \left\langle {M_{C1}^{*} ,M_{C2}^{*} ,M_{C3}^{*} ,M_{C4}^{*} ,M_{C5}^{*} ,M_{C6}^{*} ,M_{C7}^{*} ,M_{C8}^{*} } \right\rangle$,where, $M_{Cj}^{*} = \left( {\mathop {\max }\limits_{i} \mu_{ij} ,\mathop {\min }\limits_{i} \nu_{ij} } \right)$, for benefit-type criteria,

$M_{Cj}^{*} = \left( {\mathop {\min }\limits_{i} \mu_{ij} ,\mathop {\max }\limits_{i} \nu_{ij} } \right)$, for cost-type criteria, and $i = 1,2,3,4,5,6\,;\,j = 1,2,3,4,5,6,7,8$.

#### Results

The distance measure values obtained with our proposed measures are as follows.

By generalized chordal distance,

$$\begin{aligned} D_{{{\text{Chd}},1}} \left( {M_{1} ,M^{*} } \right) & = 0.3062;D_{{{\text{Chd,}}1}} \left( {M_{2} ,M^{*} } \right) = 0.2912;D_{{{\text{Chd}},1}} \left( {M_{3} ,M^{*} } \right) = 0.2952; \\ D_{{{\text{Chd}},1}} \left( {M_{4} ,M^{*} } \right) & = 0.2683;D_{{{\text{Chd}},1}} \left( {M_{5} ,M^{*} } \right) = 0.0811;D_{{{\text{Chd,}}1}} \left( {M_{6} ,M^{*} } \right) = 0.2559 \\ \end{aligned}$$

$$\begin{aligned} D_{{{\text{Chd}},2}} \left( {M_{1} ,M^{*} } \right) & = 0.3280;\;D_{{{\text{Chd}},2}} \left( {M_{2} ,M^{*} } \right) = 0.3049;D_{{{\text{Chd}},2}} \left( {M_{3} ,M^{*} } \right) = 0.3089; \\ D_{{{\text{Chd}},2}} \left( {M_{4} ,M^{*} } \right) & = 0.2985;\;D_{{{\text{Chd}},2}} \left( {M_{5} ,M^{*} } \right) = 0.1246;D_{{{\text{Chd}},2}} \left( {M_{6} ,M^{*} } \right) = 0.2757 \\ \end{aligned}$$

By non-Archimedean chordal distance,

$$\begin{aligned} D_{{{\text{nAChd}},1}}^{0.3} \left( {M_{1} ,M^{*} } \right) & = 0.8572;\;D_{{n{\text{AChd}},1}}^{0.3} \left( {M_{2} ,M^{*} } \right) = 0.8028;\;D_{{{\text{nAChd}},1}}^{0.3} \left( {M_{3} ,M^{*} } \right) = 0.8544; \\ D_{{{\text{nAChd}},1}}^{0.3} \left( {M_{4} ,M^{*} } \right) & = 0.8002;\;D_{{{\text{nAChd}},1}}^{0.3} \left( {M_{5} ,M^{*} } \right) = 0.2824;\;D_{{{\text{nAChd}},1}}^{0.3} \left( {M_{6} ,M^{*} } \right) = 0.7647 \\ \end{aligned}$$

$$\begin{aligned} D_{{{\text{nAChd}},1}}^{0.8} \left( {M_{1} ,M^{*} } \right) & = 0.2942;\;D_{{{\text{nAChd}},1}}^{0.8} \left( {M_{2} ,M^{*} } \right) = 0.2747;\;D_{{{\text{nAChd}},1}}^{0.8} \left( {M_{3} ,M^{*} } \right) = 0.2902; \\ D_{{{\text{nAChd}},1}}^{0.8} \left( {M_{4} ,M^{*} } \right) & = 0.2624;\;D_{{{\text{nAChd}},1}}^{0.8} \left( {M_{5} ,M^{*} } \right) = 0.0748;\;D_{{{\text{nAChd}},1}}^{0.8} \left( {M_{6} ,M^{*} } \right) = 0.2326 \\ \end{aligned}$$

$$\begin{aligned} D_{{{\text{nAChd}},2}}^{0.3} \left( {M_{1} ,M^{*} } \right) & = 0.6470;\;D_{{{\text{nAChd}},2}}^{0.3} \left( {M_{2} ,M^{*} } \right) = 0.6123;\;D_{{{\text{nAChd}},2}}^{0.3} \left( {M_{3} ,M^{*} } \right) = 0.6379; \\ D_{{{\text{nAChd}},2}}^{0.3} \left( {M_{4} ,M^{*} } \right) & = 0.5931;\;D_{{{\text{nAChd}},2}}^{0.3} \left( {M_{5} ,M^{*} } \right) = 0.3246;\;D_{{{\text{nAChd}},2}}^{0.3} \left( {M_{6} ,M^{*} } \right) = 0.5775 \\ \end{aligned}$$

$$\begin{aligned} D_{{{\text{nAChd}},2}}^{0.8} \left( {M_{1} ,M^{*} } \right) & = 0.4128;\;D_{{{\text{nAChd}},2}}^{0.8} \left( {M_{2} ,M^{*} } \right) = 0.3978;\;D_{{{\text{nAChd}},2}}^{0.8} \left( {M_{3} ,M^{*} } \right) = 0.4015; \\ D_{{{\text{nAChd}},2}}^{0.8} \left( {M_{4} ,M^{*} } \right) & = 0.3942;\;D_{{{\text{nAChd}},2}}^{0.8} \left( {M_{5} ,M^{*} } \right) = 0.1671;\;D_{{{\text{nAChd}},2}}^{0.8} \left( {M_{6} ,M^{*} } \right) = 0.3749 \\ \end{aligned}$$

#### Discussion

Here, we observe that the smallest distance value is obtained for the pair $\left( {M_{5} ,M^{*} } \right)$, which implies that the medicine $M_{5}$ (Tocilizumab) is suited best for treatment of COVID-19. The recovery rate of Tocilizumab is relatively better than the others if administered at the same time.

The distance measure values calculated under different distance methods are presented in Table 6.

**Table 6:** Determination of the best medicine for the treatment of COVID-19

Distance measures	Distance values between						Best medicine
	$\left( {M_{1} ,M^{*} } \right)$	$\left( {M_{2} ,M^{*} } \right)$	$\left( {M_{3} ,M^{*} } \right)$	$\left( {M_{4} ,M^{*} } \right)$	$\left( {M_{5} ,M^{*} } \right)$	$\left( {M_{6} ,M^{*} } \right)$	
${\mathcal{D}}_{H}$	3.1986	2.9826	3.1060	2.8737	0.9014	2.7033	$M_{5}$ (Tocilizumab)
${\mathbf{\mathcal{D}}}_{E}$	1.0726	1.0525	1.1224	1.0175	0.4675	1.1000	$M_{5}$ (Tocilizumab)
${\mathbf{\mathcal{D}}}_{{{\text{nH}}}}$	0.3998	0.3728	0.3884	0.3592	0.1127	0.3379	$M_{5}$ (Tocilizumab)
${\mathbf{\mathcal{D}}}_{{{\text{nE}}}}$	0.3792	0.3721	0.3968	0.3597	0.1653	0.3889	$M_{5}$ (Tocilizumab)
${\mathbf{\mathcal{D}}}_{{{\text{BA}}}}^{1}$	0.0776	0.0677	0.0790	0.0646	0.0136	0.0857	$M_{5}$ (Tocilizumab)
${\mathbf{\mathcal{D}}}_{{{\text{BA}}}}^{2}$	0.2884	0.2546	0.2942	0.2626	0.0616	0.2750	$M_{5}$ (Tocilizumab)
${\mathbf{\mathcal{D}}}_{G}$	0.4000	0.3750	0.3625	0.3500	0.1125	0.3500	$M_{5}$ (Tocilizumab)
${\mathbf{\mathcal{D}}}_{{{\text{YF}}}}$	0.4139	0.3834	0.3786	0.3603	0.1212	0.3522	$M_{5}$ (Tocilizumab)
${\mathbf{\mathcal{D}}}_{{{\text{WX}}}}$	0.3531	0.3250	0.3344	0.3125	0.0938	0.3188	$M_{5}$ (Tocilizumab)
${\mathbf{\mathcal{D}}}_{{{\text{JHP}}}}$	0.4069	0.3781	0.3834	0.3598	0.1169	0.3450	$M_{5}$ (Tocilizumab)
${\mathbf{\mathcal{D}}}_{{{\text{SF}}}}$	0.1092	0.1135	0.0962	0.1178	0.0910	0.1635	$M_{5}$ (Tocilizumab)
${\mathbf{\mathcal{D}}}_{{{\text{RX}}}}$	0.3955	0.3765	0.4287	0.3908	0.1884	0.4006	$M_{5}$ (Tocilizumab)
${\mathbf{\mathcal{D}}}_{{{\text{PY}}}}$	0.4100	0.3687	0.4087	0.3825	0.1162	0.3312	$M_{5}$ (Tocilizumab)
${\mathbf{\mathcal{D}}}_{{{\text{EA}}}}$	0.0241	0.0210	0.0218	0.0108	0.0005	0.0116	$M_{5}$ (Tocilizumab)
${\mathbf{\mathcal{D}}}_{{{\text{SB}}}}$	0.4067	0.3721	0.4179	0.4025	0.1379	0.3467	$M_{5}$ (Tocilizumab)
${\mathbf{\mathcal{D}}}_{{{\text{MP}}}}$	0.4410	0.3850	0.4506	0.4229	0.1156	0.3773	$M_{5}$ (Tocilizumab)
${\mathbf{\mathcal{D}}}_{{{\text{Chd}},1}}$	0.3062	0.2912	0.2952	0.2683	0.0811	0.2559	$M_{5}$ (Tocilizumab)
${\mathbf{\mathcal{D}}}_{{{\text{Chd}},2}}$	0.3280	0.3049	0.3089	0.2985	0.1246	0.2757	$M_{5}$ (Tocilizumab)
${\mathbf{\mathcal{D}}}_{{{\text{nAChd}},1}}^{0.3}$	0.8572	0.8028	0.8544	0.8002	0.2824	0.7647	$M_{5}$ (Tocilizumab)
${\mathbf{\mathcal{D}}}_{{{\text{nAChd}},1}}^{0.8}$	0.2942	0.2747	0.2902	0.2624	0.0748	0.2326	$M_{5}$ (Tocilizumab)
${\mathbf{\mathcal{D}}}_{{{\text{nAChd}},2}}^{0.3}$	0.6470	0.6123	0.6379	0.5931	0.3246	0.5775	$M_{5}$ (Tocilizumab)
${\mathbf{\mathcal{D}}}_{{{\text{nAChd}},2}}^{0.8}$	0.4128	0.3978	0.4015	0.3942	0.1671	0.3749	$M_{5}$ (Tocilizumab)

**Fig. 13:**
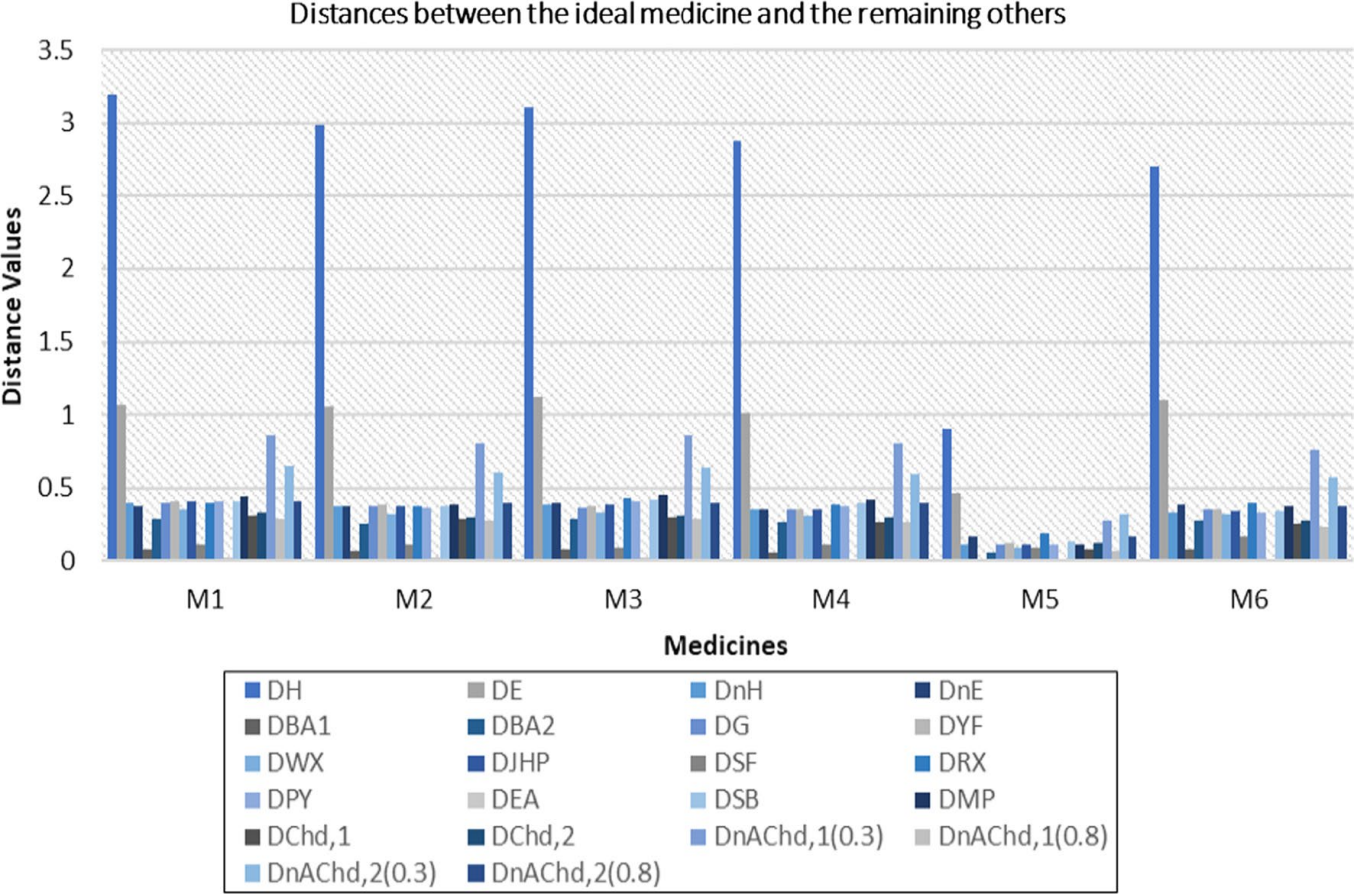
Distance measure values between the ideal medicine and the available set of medicines

Furthermore, a graphical representation of distance measure values and the most optimum medicine being $M_{5}$ is evident from Fig. 13, as shown below.

### Sensitivity analysis

The two newly proposed distances in this article, viz*.* generalized chordal distance and non-Archimedean chordal distance involve two input parameters ‘$p$’ and ‘$\lambda$’ in their expressions. And with respect to different values of these input parameters, Eqs. (23) and (32) undergo different transformations. Therefore, it is necessary to analyze the influence of these changing parameters $p$ and $\lambda$ on the final ranking yielded under different application scenarios. For different values of these input parameters, if the final ranking order remains unchanged, only then we can establish the validity of the results. Therefore, it becomes an integral component of any multicriteria decision-making problem to conduct a sensitivity analysis as such to get a clear idea of the sensitiveness of the proposed model towards the involved parameters.

In our present study, we have demonstrated three suitable applications of our proposed measures in problems of pattern recognition, medical diagnosis, and medicine selection for COVID-19, respectively. In each of these case studies, we have considered some particular values of these input parameters for our proposed definitions, such as, $p = 1,2\,\,;\,\lambda = 0.2,0.6$ (pattern recognition), $p = 1,2\,\,;\,\lambda = 0.5,0.7$ (medical diagnosis), and $p = 1,2\,\,;\,\lambda = 0.3,0.8$ (medicine selection). We have then evaluated the ranking orders of the available alternatives. However, in this section, in order to examine the effect of the parameter $\lambda$ on the final ranking order of the alternatives, we assume several values of $\lambda$ from 0 to 1. While on the other hand, there is no such restriction on the value of the parameter $p$.

In Tables 7, 8, and 9, as shown, we assign the values of $p$ as $p = 1,2,5,10,50$, the values of $\lambda$ as $\lambda = 0,0.4,0.6,0.8,1$, and obtain the preference ranking of the available alternatives. We can observe that the ranking order obtained for different values of the input parameters is coherent with the initial ranking order. It implies that the ranking results are reliable and that the changes in the input parameter values are not capable of changing the best option and the initial credible rank. Further, we also observe that, for a particular value of $p$, as the value of the parameter $\lambda$ increases, the distance measure value decreases. This implies that the randomness or farness decreases and equivalently the degree of likeliness increases, which may be referred to as an optimistic decision approach. While for smaller values of $\lambda$, we obtain a subsequently higher value of distance measure, which can be regarded as a decision of pessimism among the decision-makers. Thus, our approach provides extreme flexibility to the decision-makers to choose the values of the input parameters as per their requirement and practicality.

**Table 7:** Effect of the parameters $p$ and $\lambda$ on the ranking order for the pattern recognition problem

Values of $p$ and $\lambda$	Generalized chordal distance						Non-Archimedean chordal distance
	$\left( {P_{1} ,Q} \right)$	$\left( {P_{2} ,Q} \right)$	$\left( {P_{3} ,Q} \right)$	$\left( {P_{1} ,Q} \right)$	$\left( {P_{2} ,Q} \right)$	$\left( {P_{3} ,Q} \right)$	
*p* = 1	$\lambda = 0$	0.5101	0.2469	0.5172	0.5162	0.3976	0.5312
	$\lambda = 0.4$				0.3605	0.2141	0.3722
	$\lambda = 0.6$				0.1624	0.0667	0.1703
	$\lambda = 0.8$				0.1422	0.0472	0.1510
	$\lambda = 1$				0.1187	0.0266	0.1275
Ranking		$P_{2} < P_{1} < P_{3}$			$P_{2} < P_{1} < P_{3}$		
*p* = 2	$\lambda = 0$	0.8527	0.5334	0.8649	0.5374	0.4272	0.5431
	$\lambda = 0.4$				0.4642	0.2973	0.4723
	$\lambda = 0.6$				0.1628	0.1042	0.1718
	$\lambda = 0.8$				0.1478	0.0529	0.1619
	$\lambda = 1$				0.1214	0.0482	0.1362
Ranking		$P_{2} < P_{1} < P_{3}$			$P_{2} < P_{1} < P_{3}$		
*p* = 5	$\lambda = 0$	0.8755	0.5773	0.8799	0.5441	0.4363	0.5662
	$\lambda = 0.4$				0.4770	0.3009	0.4846
	$\lambda = 0.6$				0.1739	0.1124	0.1826
	$\lambda = 0.8$				0.1581	0.0606	0.1685
	$\lambda = 1$				0.1313	0.0584	0.1445
Ranking		$P_{2} < P_{1} < P_{3}$			$P_{2} < P_{1} < P_{3}$		
*p* = 10	$\lambda = 0$	0.8932	0.6077	0.9038	0.5526	0.4423	0.5692
	$\lambda = 0.4$				0.4876	0.3135	0.4962
	$\lambda = 0.6$				0.1861	0.1352	0.1947
	$\lambda = 0.8$				0.1621	0.0771	0.1742
	$\lambda = 1$				0.1444	0.0609	0.1589
Ranking		$P_{2} < P_{1} < P_{3}$			$P_{2} < P_{1} < P_{3}$		
*p* = 50	$\lambda = 0$	0.9210	0.6679	0.9442	0.5692	0.4649	0.5773
	$\lambda = 0.4$				0.4946	0.3215	0.5016
	$\lambda = 0.6$				0.1928	0.1497	0.2040
	$\lambda = 0.8$				0.1701	0.0808	0.1777
	$\lambda = 1$				0.1470	0.0737	0.1612
Ranking		$P_{2} < P_{1} < P_{3}$			$P_{2} < P_{1} < P_{3}$

**Table 8:** Effect of the parameters $p$ and $\lambda$ on the ranking order for the medical diagnosis problem

Values of $p$ and $\lambda$	Generalized chordal distance										Non-archimedean chordal distance
	$\left( {P_{1} ,Q} \right)$	$\left( {P_{2} ,Q} \right)$	$\left( {P_{3} ,Q} \right)$	$\left( {P_{4} ,Q} \right)$	$\left( {P_{5} ,Q} \right)$	$\left( {P_{1} ,Q} \right)$	$\left( {P_{2} ,Q} \right)$	$\left( {P_{3} ,Q} \right)$	$\left( {P_{4} ,Q} \right)$	$\left( {P_{5} ,Q} \right)$	
*p* = 1	$\lambda = 0$	0.0912	0.5321	0.6036	0.3418	0.3477	0.1950	0.6602	0.6821	0.4408	0.5606
	$\lambda = 0.4$						0.1502	0.6345	0.6404	0.4166	0.5105
	$\lambda = 0.6$						0.0921	0.3973	0.4051	0.2677	0.2954
	$\lambda = 0.8$						0.0601	0.2668	0.2724	0.1740	0.1904
	$\lambda = 1$						0.0371	0.1738	0.1801	0.1115	0.1238
Ranking		$P_{1} < P_{4} < P_{5} < P_{2} < P_{3}$					$P_{1} < P_{4} < P_{5} < P_{2} < P_{3}$				
*p* = 2	$\lambda = 0$	0.2924	0.7636	0.8522	0.5426	0.6009	0.2943	0.7256	0.7450	0.5002	0.6309
	$\lambda = 0.4$						0.2713	0.7030	0.7110	0.4812	0.5801
	$\lambda = 0.6$						0.1567	0.6404	0.6654	0.4463	0.4902
	$\lambda = 0.8$						0.1001	0.4325	0.4526	0.2846	0.3131
	$\lambda = 1$						0.0622	0.2813	0.3007	0.1821	0.2018
Ranking		$P_{1} < P_{4} < P_{5} < P_{2} < P_{3}$					$P_{1} < P_{4} < P_{5} < P_{2} < P_{3}$				
*p* = 5	$\lambda = 0$	0.3113	0.7785	0.8694	0.5649	0.6216	0.4601	0.9328	0.9855	0.7031	0.7530
	$\lambda = 0.4$						0.4244	0.8840	0.9003	0.7865	0.7308
	$\lambda = 0.6$						0.2175	0.6807	0.7215	0.5602	0.5976
	$\lambda = 0.8$						0.1404	0.6506	0.6955	0.4438	0.5052
	$\lambda = 1$						0.0901	0.4202	0.4618	0.2834	0.3228
Ranking		$P_{1} < P_{4} < P_{5} < P_{2} < P_{3}$					$P_{1} < P_{4} < P_{5} < P_{2} < P_{3}$				
*p* = 10	$\lambda = 0$	0.3258	0.7813	0.8698	0.5682	0.6289	0.5322	0.9416	0.9601	0.7842	0.8001
	$\lambda = 0.4$						0.5103	0.8621	0.9315	0.6501	0.7751
	$\lambda = 0.6$						0.2514	0.8409	0.9168	0.5952	0.6925
	$\lambda = 0.8$						0.1626	0.8024	0.8908	0.5620	0.6645
	$\lambda = 1$						0.1036	0.5137	0.5872	0.3601	0.4244
Ranking		$P_{1} < P_{4} < P_{5} < P_{2} < P_{3}$					$P_{1} < P_{4} < P_{5} < P_{2} < P_{3}$				
*p* = 50	$\lambda = 0$	0.3474	0.8085	0.8767	0.5727	0.6378	0.6205	0.8178	0.8520	0.6575	0.7063
	$\lambda = 0.4$						0.5974	0.7924	0.8367	0.6220	0.7605
	$\lambda = 0.6$						0.3109	0.7306	0.8134	0.5109	0.6025
	$\lambda = 0.8$						0.2041	0.6655	0.7804	0.4838	0.5853
	$\lambda = 1$						0.1308	0.6510	0.7673	0.4644	0.5578
Ranking		$P_{1} < P_{4} < P_{5} < P_{2} < P_{3}$					$P_{1} < P_{4} < P_{5} < P_{2} < P_{3}$

**Table 9:** Effect of the parameters $p$ and $\lambda$ on the ranking order for the medicine selection problem

Values of $p$ and $\lambda$		Generalized chordal distance						Non-Archimedean chordal distance					
		$\left( {M_{1} ,M^{*} } \right)$	$\left( {M_{2} ,M^{*} } \right)$	$\left( {M_{3} ,M^{*} } \right)$	$\left( {M_{4} ,M^{*} } \right)$	$\left( {M_{5} ,M^{*} } \right)$	$\left( {M_{6} ,M^{*} } \right)$	$\left( {M_{1} ,M^{*} } \right)$	$\left( {M_{2} ,M^{*} } \right)$	$\left( {M_{3} ,M^{*} } \right)$	$\left( {M_{4} ,M^{*} } \right)$	$\left( {M_{5} ,M^{*} } \right)$	$\left( {M_{6} ,M^{*} } \right)$
*p* = 1	$\lambda = 0$	0.3062	0.2912	0.2952	0.2683	0.0811	0.2559	0.9063	0.8209	0.8447	0.7830	0.6962	0.7254
	$\lambda = 0.4$							0.7062	0.6700	0.6998	0.6202	0.2142	0.6175
	$\lambda = 0.6$							0.4620	0.4342	0.4391	0.3999	0.1301	0.3726
	$\lambda = 0.8$							0.2942	0.2747	0.2902	0.2624	0.0748	0.2326
	$\lambda = 1$							0.1999	0.1864	0.1941	0.1796	0.0563	0.1690
Ranking		$M_{5} < M_{6} < M_{4} < M_{2} < M_{3} < M_{1}$						$M_{5} < M_{6} < M_{4} < M_{2} < M_{3} < M_{1}$					
*p* = 2	$\lambda = 0$	0.3280	0.3049	0.3089	0.2985	0.1246	0.2757	0.9113	0.8261	0.8516	0.7848	0.7278	0.7349
	$\lambda = 0.4$							0.7171	0.6836	0.7025	0.6219	0.4831	0.6197
	$\lambda = 0.6$							0.4798	0.4431	0.4479	0.4080	0.2695	0.3729
	$\lambda = 0.8$							0.4128	0.3978	0.4015	0.3942	0.1671	0.3749
	$\lambda = 1$							0.2006	0.1882	0.1950	0.1831	0.1169	0.1744
Ranking		$M_{5} < M_{6} < M_{4} < M_{2} < M_{3} < M_{1}$						$M_{5} < M_{6} < M_{4} < M_{2} < M_{3} < M_{1}$					
*p* = 5	$\lambda = 0$	0.3303	0.3180	0.3294	0.3099	0.1470	0.2958	0.9137	0.8338	0.8690	0.7936	0.7587	0.7609
	$\lambda = 0.4$							0.7256	0.6934	0.7104	0.6297	0.5643	0.6272
	$\lambda = 0.6$							0.4868	0.4455	0.4537	0.4087	0.2888	0.3838
	$\lambda = 0.8$							0.4198	0.4072	0.4145	0.4008	0.1989	0.3804
	$\lambda = 1$							0.2044	0.1936	0.1958	0.1839	0.1379	0.1799
Ranking		$M_{5} < M_{6} < M_{4} < M_{2} < M_{3} < M_{1}$						$M_{5} < M_{6} < M_{4} < M_{2} < M_{3} < M_{1}$					
*p* = 10	$\lambda = 0$	0.3522	0.3313	0.3368	0.3195	0.1630	0.3015	0.9236	0.8392	0.8772	0.8008	0.7735	0.7767
	$\lambda = 0.4$							0.7373	0.7056	0.7123	0.6393	0.5790	0.6325
	$\lambda = 0.6$							0.4877	0.4590	0.4690	0.4164	0.2921	0.3996
	$\lambda = 0.8$							0.4269	0.4152	0.4273	0.4100	0.2145	0.3989
	$\lambda = 1$							0.2120	0.1976	0.1963	0.1897	0.1672	0.1841
Ranking		$M_{5} < M_{6} < M_{4} < M_{2} < M_{3} < M_{1}$						$M_{5} < M_{6} < M_{4} < M_{2} < M_{3} < M_{1}$					
*p* = 50	$\lambda = 0$	0.3809	0.3539	0.3658	0.3339	0.1940	0.3222	0.9557	0.8989	0.9172	0.8567	0.8300	0.8448
	$\lambda = 0.4$							0.7519	0.7196	0.7456	0.6598	0.6198	0.6346
	$\lambda = 0.6$							0.4907	0.4629	0.4761	0.4331	0.3071	0.4123
	$\lambda = 0.8$							0.4305	0.4247	0.4370	0.4298	0.2489	0.4067
	$\lambda = 1$							0.2464	0.2323	0.2361	0.2256	0.1847	0.2198
Ranking		$M_{5} < M_{6} < M_{4} < M_{2} < M_{3} < M_{1}$						$M_{5} < M_{6} < M_{4} < M_{2} < M_{3} < M_{1}$					

## Concluding remarks

Distance measure may be employed as a quantitative tool to measure the dissimilarity in order to differentiate between two PFSs. PFSs are special type of sets with nonlinear characteristics. Therefore, a sincere attempt has been made to construct two nonlinear distances for PFSs, since it is a very much daunting task to construct the concept of a single “universal distance” for PFSs owing to their complex arithmetic operations over IFSs. To validate the veracity of the newly constructed distances, twelve different sets of IFNs are considered where the proficiency of our proposed measures are established by means of a meticulous comparative analysis. Moreover, to demonstrate the efficacy of our newly constructed distances, empirical applications from the field of pattern recognition, medical diagnosis, and optimum medicine selection have been considered. From the studies conducted it can be ascertained that the proposed measures have a valid structural formulation and are capable of achieving the targets of real-life decision-making problems.

The contributions and originality of our work can be pinned down to the points:

- Proposed two nonlinear distances to measure the distance between PFSs.
- Proposed measures are shown to be efficient over most of the established distance measures and hence their significance.
- Proposed measures are capable of handling application-oriented problems from the field of pattern recognition, medical diagnosis, COVID-19 led scenarios, etc.

The advantages of our article can be stated as:

- Proposed measures are capable of handling situations when classical set theory, fuzzy set theory, paraconsistent set theory, IFSs theory fails to be applied.
- Proposed measures are capable of handling both linear as well as nonlinear data or information.
- Proposed measures are capable of handling incomplete information and also indeterminate or inconsistent information as well.
- Proposed measures have more suitable engineering and scientific applications.
- Moreover, PFSs theory has not been previously explored in this direction, which is our motivation and hence the novelty of this work.

In the future direction, the concept of such nonlinear distances can be further extended to some other special datasets having applications in various other fields. Further, an attempt shall be made to construct some efficient aggregation operators for tackling various decision-making problems with multiple criteria.

## Data Availability

Not applicable.

## Abbreviations

PFS: Pythagorean fuzzy set
IFS: Intuitionistic fuzzy set
COVID-19: Corona virus December-2019
MCDM: Multicriteria decision making
TOPSIS: Technique for Order Preference by Similarity to an Ideal Solution
OWA: Ordered weighted averaging
MERS: Middle East respiratory syndrome
SARS: Severe acute respiratory syndrome
WHO: World Health Organization
FDA: Food and Drug Administration
HIV: Human immunodeficiency virus
AIDS: Acquired immune deficiency syndrome
TODIM: An acronym in Portuguese of interactive and multicriteria decision making
PFN: Pythagorean fuzzy numbers
